# Assessment of Four Theoretical Approaches to Predict Protein Flexibility in the Crystal Phase and Solution

**DOI:** 10.1021/acs.jctc.4c00754

**Published:** 2024-08-22

**Authors:** Ł. J. Dziadek, A. K. Sieradzan, C. Czaplewski, M. Zalewski, F. Banaś, M. Toczek, W. Nisterenko, S. Grudinin, A. Liwo, A. Giełdoń

**Affiliations:** † Faculty of Chemistry, University of Gdansk, ul. Wita-Stwosza 63, 80-308 Gdańsk, Poland; ‡ School of Computational Sciences, Korea Institute for Advanced Study, 85 Hoegiro, Dongdaemun-gu, Seoul 02455, Republic of Korea; § LJK, University Grenoble Alpes, CNRS, Grenoble INP, F-38000 Grenoble, France

## Abstract

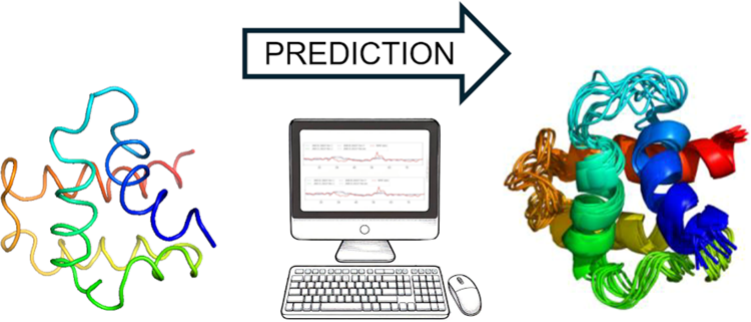

In this paper, we evaluated the ability of four coarse-grained methods to predict protein flexible regions with potential biological importance, UNRES-flex, UNRES-DSSP-flex (based on the united residue model of polypeptide chains without and with secondary structure restraints, respectively), CABS-flex (based on the C-α, C-β, and side chain model), and nonlinear rigid block normal mode analysis (NOLB) with a set of 100 protein structures determined by NMR spectroscopy or X-ray crystallography, with all secondary structure types. End regions with high fluctuations were excluded from analysis. The Pearson and Spearman correlation coefficients were used to quantify the conformity between the calculated and experimental fluctuation profiles, the latter determined from NMR ensembles and X-ray *B*-factors, respectively. For X-ray structures (corresponding to proteins in a crowded environment), NOLB resulted in the best agreement between the predicted and experimental fluctuation profiles, while for NMR structures (corresponding to proteins in solution), the ranking of performance is CABS-flex > UNRES-DSSP-flex > UNRES-flex > NOLB; however, CABS-flex sometimes exaggerated the extent of small fluctuations, as opposed to UNRES-DSSP-flex.

## 1 Introduction

The flexibility of proteins is crucial to fulfill their biological role.^1^ For example, contemporary descriptions of agonist and antagonist effects incorporate the dynamic interconversion between inactive and activated states of proteins. An agonist molecule facilitates the shift of a protein toward the activated conformation by selectively binding to it.^2^ As a consequence, the information on protein flexibility is essential in drug design.^3^ When the ligand is attached to the protein, it can induce a cascade of motions, resulting in a conformational change.^2^ However, if the ligand-binding pocket shows high structural fluctuations, this information should be included in the prediction of ligand–protein interactions.

Nuclear magnetic resonance (NMR) spectroscopy is one of the most powerful tools to study protein flexibility. NMR measurements give the information on the contact distances (typically 5 Å or less) between paramagnetic nuclei (mostly protons) based on the nuclear Overhauser effect (NOE) and local structure based on the chemical shifts thereof. Because the measured quantities are averaged over at least a millisecond time scale, NMR structure determination results in an ensemble, from which flexibility can be estimated right away.^4^ NMR structures are usually diffuse in regions with scarce NOE signals (e.g., flexible ends or loops), which indicates that most of the contacts are averaged over the period of mixing time and, consequently, not observed. Thus, qualitatively, NMR provides information on flexible regions. However, it must be borne in mind that with sparse experimental restraints, the ensemble diversity heavily depends on the force field.

Thus, qualitatively, NMR provides the information on flexible regions; however, it must be kept in mind that with sparse experimental restraints, the ensemble diversity heavily depends on the force field. Additionally, relaxation experiments can be performed, from which the *S*^2^ order parameters can be determined,^5^ which provide direct information on flexibility.

About 90% of the structures in the protein databank (PDB)^6^ were solved by X-ray crystallography, which continues to provide the highest resolution. As opposed to the NMR spectroscopy, which treats protein molecules in solution, the X-ray measurements are performed on crystals in which the atoms can only fluctuate about equilibrium positions. The extents of these fluctuations (and, thereby, flexibility) are related to the Debye–Waller factors (*B*-factors) of the respective atoms through a simple formula (see Section 2).^7^ Even though the *B*-factors do not capture the full flexibility of proteins in solutions,^4^ they are still good indicators of the regions with high flexibility (e.g., flexible loops). It should be noted, however, that the *B*-factors are influenced by the refinement procedure and crystal defects, diffraction decay, and other factors.^8,9^

The core of a structure is usually defined equally well regardless of whether it has been solved by X-ray or NMR; however, for the reasons pointed out above, the loops in crystal structures appear too rigid, while those in the NMR structures are too “floppy”.^10^

Four basic types of computational approaches can be used to estimate the protein flexibility: (i) molecular dynamics (MD),^11−13^ (ii) Monte Carlo (MC) methods,^14^ (iii) elastic^15^ and Gaussian^16^ network models, and (iv) normal mode analysis (NMA).^17^ Like NMR structure determination, MD and MC result in conformational ensembles, from which it is straightforward to quantify the flexibility of the respective regions. Both all-atom and coarse-grained models are used here. Compared to all-atom models, the coarse-grained approaches cover about 3 orders of magnitude wider time scale (due to averaging out the degrees of freedom absent from the model), which enables much more extensive conformational sampling.^19^ Moreover, coarse-grained models are computationally less expensive compared to all-atom simulations, which require substantial computational resources to carry out. The reduction of the complexity of the system by treating groups of atoms as a single entity simplifies the modeling process and can lead to a better understanding of the system’s behavior. All-atom simulations can suffer from insufficient “sampling” due to their high dimensionality. On the other hand, many coarse-grained models cover specific kinds of molecules (e.g., proteins), while all-atom models are more easily generalizable to other systems. In the elastic network models, atoms whose distances are smaller than a preassigned cutoff distance are linked with springs with equal force constants, while in the Gaussian network models, the force constants depend on distances. Usually, in both approaches, an amino acid residue is represented by the Cα atom; however, all-atom variants of both approaches are also used. Finally, the normal mode analysis uses the complete energy Hessian at the potential energy minimum (see Section 2.4).

As the disparity between the number of solved protein structures and that of known protein sequences continues to widen, computational tools for accurate prediction of protein flexibility solely from amino acid sequences would be the best solution. MEDUSA^20^ is one of the methods for predicting protein flexibility using the sequence information alone, which utilizes evolutionary insights from sequences of homologous proteins and the physicochemical properties of amino acids. cdsAF2 is another method, based on AlphaFold2,^21^ which integrates pairwise geometric features with multiple sequence alignments. These approaches facilitate the identification of potentially highly deformable protein regions and provide insights into the general dynamic properties of proteins. However, methods for flexibility prediction based on protein dynamics are still more accurate.

The purpose of this work was to evaluate the accuracy of protein flexibility predictions by using four coarse-grained methods. Two of those are based on the coarse-grained united residue (UNRES)^22^ model implemented (i) in the unrestrained mode (termed UNRES-flex) and (ii) with secondary structure restraints based on Dictionary of Protein Secondary Structure (DSSP)^23^ assignment (termed UNRES-DSSP-flex). The next approach is based on (iii) the C-α, C-β, and side chain (CABS)^24^ coarse-grained model (termed CABS-flex), and the last one (iv) is the nonlinear rigid block normal mode analysis (NOLB)^17^ approach. These approaches are based on canonical molecular dynamics (UNRES-flex and UNRES-DSSP-flex), Monte Carlo dynamics (CABS-flex), and normal mode analysis (NOLB), respectively. All these approaches are computationally fast and not resource-demanding. We show that these methods result in reliable flexibility prediction.

## 2 Methods

### 2.1 Test Set

The test set consisted of 100 proteins, which were already used to evaluate the prediction capability of UNRES.^25^ This set contains proteins with various structures (30 α-helical, 21 β-sheet, and 49 α + β) determined by NMR (50 structures) and X-ray (50 structures). In Table S1 of the Supporting Information, the benchmark proteins are grouped according to the secondary structure and structure determination method. The selected proteins are single-chain globular proteins. Most of them were taken from the benchmark set of 69 proteins with various structural types used to test the latest version of UNRES,^25^ which were selected to contain less than 200 residues, all secondary structure types (α, β, and α + β), and no missing coordinates in the structures.^25^ Because ab initio folding simulations were not carried out in this work, the benchmark set of ref (25) was extended by larger proteins. Finally, the set contained 79 proteins with chain length less than 100 residues, 11 from more than 100 and less than 200 residues, and 10 larger than 200 residues. The smallest and largest chain lengths were 20 and 532 residues, respectively. None of them was used in parametrizing the variant of UNRES applied in this work.^25^ Detailed information on the test-set proteins, including their chain lengths, can be found in Table S1 of the Supporting Information.

### 2.2 UNRES-FLEX and UNRES-DSSP-FLEX

The UNRES-flex and UNRES-DSSP-flex methods are based on the UNRES coarse-grained model of polypeptide chains, in which a polypeptide chain is represented by a sequence of α-carbon (Cα) atoms linked with virtual bonds, with peptide groups (p) located halfway between the consecutive Cαs and united side chains (SCs) attached to the Cαs with the Cα-SC virtual bonds. Only the united peptide groups and the united side chains are interaction sites, while the Cαs assist in the chain geometry definition. The effective energy function has been developed on a physical basis, by expressing the potential of mean force in terms of Kubo cluster cumulant functions,^26^ which are approximated analytically by Kubo cluster cumulants. The energy function is expressed by eq 1(25)
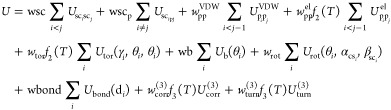
1where *U*_SC*_i_*SC*_j_*_ represents the mean free energy of the hydrophobic (hydrophilic) interactions between the side chains, *U*_SC*_i_*p*_j_*_ denotes the excluded volume potential of the side chain–peptide group interactions, *U*_p*_i_*p_*j*__ describes the peptide–peptide group interaction potential, *U*_bond_(*d*_*i*_), are simple harmonic potentials of the virtual bond where *d*_*i*_ is the length of *i*th virtual bond, *U*_tor_, *U*_tord_, *U*_b_, and *U*_rot_ are the virtual bond–dihedral angle torsion terms, and *U*_corr_^(3)^ and *U*_turn_^(3)^ account for the coupling between the backbone local and backbone–electrostatic interactions, respectively. The solvent is implicit in UNRES and protein–solvent interactions are contained in the effective energy terms of eq 1, mainly in *U*_SC*_i_*SC*_j_*_. The factors *f*_*n*_ account for the dependence of the effective energy function on temperature, this reflecting the fact that it corresponds to the potential of mean force (restricted free energy) and not potential energy. The factors are expressed by eq 2(27)
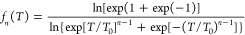
2where *T*_0_ = 300 K. The main conformational search engine used with UNRES is Langevin molecular dynamics, which was implemented in our earlier work.^28,29^ UNRES has been successful in protein structure prediction,^21^ studying protein folding dynamics and thermodynamics,^30^ and solving biological problems.^31^

In this work, short MD simulations were conducted. A total of 200,000 steps were performed with a time step of 4.89 fs, which gives about 1 ns trajectory length. However, as the UNRES time unit amounts to about 1000 laboratory time units, due to averaging over the degrees of freedom not included in the model,^22,32^ each simulation effectively corresponded to 1 μs laboratory time. The newest NEWCT-9P version of the UNRES force field parametrized by using the experimental conformational ensembles of nine proteins with various secondary structures^25^ was used. The temperature in Langevin dynamics simulations was set to 300 K, and the friction of water was scaled by the factor of 0.01 as in our previous work.^29^ It should be noted that Langevin dynamics provides thermostatting. We term UNRES-flex the method of predicting protein fluctuations based on canonical Langevin MD simulations with UNRES.

In part of the simulations, restraints were imposed on the selected Cα···Cα···Cα···Cα backbone virtual bond–dihedral angles to restrain the secondary structure,^23^ determined by DSSP,^23^ entirely based on the backbone hydrogen bonds, as defined by an electrostatic model.^33^ Flat-bottom quartic restraints with the force constant equal to 50 kcal/mol/rad^4^ were applied with a fourth-order flat-bottom range of about 50 ± 20° for α-helical and 180 ± 40° for β-sheet regions. For better probing of the conformational space, three independent MD simulations were performed. MD simulations were carried out with the same settings as the regular UNRES MD simulations. We term the above approach to protein flexibility prediction the UNRES-DSSP-flex method.

### 2.3 CABS-FLEX

CABS-flex is based on the CABS model of polypeptide chains, which is a medium-resolution coarse-grain model,^34^ in which the backbone is represented by consecutively linked Cα atoms, with virtual peptide group sites located in the centers of the Cα···Cα virtual bonds, and each side chain is represented by the Cβ atom and a united site that encompasses the respective side-chain atoms next to Cβ. The polypeptide chains are superposed on a high-resolution cubic lattice. This model utilizes Monte Carlo dynamics with the asymmetric Metropolis scheme, satisfying the requirements of microscopic reversibility.^14^ Owing to the possibility of precomputing most of the energy components, the lattice representation enables very fast sampling of the conformational space. The CABS model utilizes secondary structure data that are automatically determined by DSSP.^23^ The secondary structure data are simplified to helix/β/coil representation, the “coil” designation representing all secondary structures except for α-helix and β-sheet structures.^35^ The energy is expressed by eq 3 (ref (14))
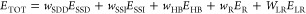
3where *E*_SSD_ (with weight *w*_SSD_ = 1.0) is the energy of short-range sequence-independent interactions, *E*_SSI_ (with weight *w*_SSI_ = 0.375) is the energy of short-range sequence-dependent interactions, *E*_HB_ (with weight *w*_HB_ = 1.0) is the hydrogen bond energy, *E*_R_ (with weight *w*_R_ = 1.0) is the energy of repulsive interactions, and *E*_LR_ (with weight of *w*_LR_ = 2.0) is the energy of long-range pairwise interactions, calculated after summing up all pairwise interactions. For details, see ref (14).

CABS was applied to simulate protein dynamics^24^ and has been used to study protein–protein interactions^35^ and conformational changes and to predict protein flexibility.^35,36,14^ It is an integral component of CABS-DOCK software, which also includes protein–peptide docking.^35^

Compared to sequence-based fluctuation predictors, CABS-flex can detect nonobvious, potentially biologically relevant, dynamic fluctuations in regions considered to be rigid, e.g., those corresponding to well-defined secondary structure elements.^37^ The obtained fluctuation profiles can be used to identify functionally important motions, the most mobile structural fragments, which are potential targets for molecular docking.^38^

In this work, simulations were carried out with CABS-flex (standalone version)^34^ using the default settings. Restraints were generated only for pairs of residues corresponding to a regular secondary structure (helical or sheet; the “ss2 mode”) and Cα..Cα distance between 3.8 and 8.0 Å (the ‘gap3′ option). Reduced temperature was set at 1.4, as recommended by the authors (a value of 1.0 is generally close to the temperature of the crystal, while a value of 2.0 typically causes the complete unfolding of unrestrained small protein chains).^36^ Three independent Monte Carlo simulations were performed for each system.

### 2.4 NOLB

NOLB is based on the normal mode analysis (NMA) technique.^17^ The harmonic anisotropic elastic network (AEN) model is used to express the potential energy, as given by eq 4
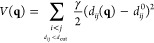
4where **q** is the vector of generalized coordinates, *d*_*ij*_(**q**) and *d*_*ij*_^0^ are the distance between the *i*th and *j*th atoms and the distance in the reference (energy-minimum) structure, respectively, and γ is the force constant. The normal modes are obtained by diagonalization of the Hessian matrix of the potential energy (given by eq 4). Coarse-graining protein structures into rigid blocks makes NOLB computationally efficient.^17^

Each mode defines a collective displacement (a sequence of rotations and translations) of the consecutive one.^17^ The displacements are scaled by the desired amplitude A. To handle large amplitudes, the total displacements can be optionally divided into several steps, which are applied iteratively.^17,18^ This procedure considerably reduces the valence geometry violations compared to Cartesian coordinate NMA.^17^ The NMA has been applied to various biomolecular systems, including proteins,^17^ RNA,^17^ and DNA,^17^ and to study protein–ligand binding,^39^ protein–protein interactions,^40^ and protein folding.^41^

### 2.5 Analysis of Simulation Results

To assess the quality of fluctuation prediction by each of the four methods considered in this work (UNRES-flex, UNRES-DSSP-flex, CABS-flex, and NOLB, respectively) in an objective manner, we used the root mean square fluctuation (RMSF)^42^ analysis. For residue with index *i*, RMSF is defined by eq 5 (ref (42))
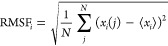
5where *x*_*i*_(*j*) is the position of the *i*th Cα atom of a given *j*th snapshot or *j*th NMR model and ⟨*x*_*i*_⟩ is the position of the *i*th Cα atom averaged over the respective simulation or NMR ensemble. The RMSF profile of a given protein shows its flexibility along the chain.^43^

The fluctuation profiles from NMR, UNRES-flex, UNRES-DSSP-flex, and NOLB ensembles were calculated as follows. First, the Cα traces of all structures were superposed on that of the first structure of the respective batch. Subsequently, the mean structure was calculated by averaging the Cα Cartesian coordinates and each structure was superposed on the mean structure and the mean structure was calculated again. There was no need to iterate the procedure further because the mean structures of the second iteration were already very close to those of the first one. The RMSF profiles were calculated taking the mean structures as references (eq 5). The RMSF profiles from CABS-flex were output directly by the CABS-flex program.^14^

For X-ray structures, RMSF is related to the *B*-factor, as approximately expressed by eq 6 (ref (44)).
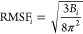
6where *B*_*i*_ is the *B*-factor of residue *i*. We shall refer to the RMSF values obtained by the respective simulation as “predicted” and to those calculated from NMR ensembles or *B*-factors as “experimental”. One can, in principle, obtain slightly better fits to the crystallographic *B*-factors if one accounts for rigid body crystallographic disorder by, e.g., introducing additional rigid body disorder parameters for each PDB structure and optimizing them mutually with a regression.^45^ However, since our main goal was a relative comparison of the four simulation techniques, we omitted this additional disorder correction in our computations.

We compared the RMSF profiles obtained with the respective methods with those calculated from NMR ensembles or *B*-factors. As measures of profile similarity, we used the Pearson product–moment correlation coefficient (*r*_p_)^46^ and the Spearman rank correlation coefficient (*r*_s_).^47^ These are expressed by eqs 7 and 8, respectively
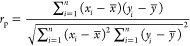
7where *x*_*i*_ and *y*_*i*_ are the predicted and experimental RMSF values for residue with index *i*, respectively, *x̅* and *y̅* are the RMSFs averaged over all residues, and *n* is the number of residues.
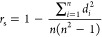
8where *d*_*i*_ is the difference between the ranks of the predicted and experimental RMSF for residue with index *i*. The rank is the position of residue *i* obtained when residues are sorted according to ascending RMSF values. The Pearson correlation coefficient is close to 1 (correlation) or −1 (anticorrelation) if the two profiles are linearly (homothetically) related to each other, whereas the Spearman correlation coefficient is close to 1 if they vary concurrently or to −1 if they vary countercurrently. A value close to 0 indicates no correlation/anticorrelation or concurrency/counterconcurrency.^48^

The fluctuations of N- and C-terminal sections of single-chain proteins are usually significantly higher than those of the remaining sections of the structure. This feature is most pronounced for NMR structures. The fluctuations of the terminal parts are nonspecific and could thus blur the fluctuations in loop regions, which usually contribute to functionally important motions. Thus, the analysis of the flexibility of a protein performed with the N- and C-terminus included may bias the correlation results. To compare the calculated and experimental fluctuation profiles, we, therefore, removed the terminal regions, by using the procedures described below for the X-ray and the NMR structures. However, the simulations (for UNRES-flex, UNRES-DSSP-flex, and CABS-flex) or normal mode calculations (for NOLB) were performed for complete structures.

For each X-ray structure, the RMSF profile was calculated from the *B*-factors (eq 6) over the whole protein. Subsequently, the average (over all residues) RMSF value () and its standard deviation () were calculated. Finally, the terminal segments were eliminated such that  for *i* = 1, 2, ···, lnt and *i* = *n*, *n* – 1, ···, *n* – lct + 1, where lnt and lct are the lengths of the eliminated N- and the C-terminal segments, respectively.

For each NMR ensemble, the mean structure and RMSF profile were determined over the whole structure as described earlier in this section. Subsequently, the average RMSF and its standard deviation were calculated and the terminal segments with  were eliminated. This procedure was repeated for the truncated chain; however, in most cases, further deletions were not required.

Because different methods result in different RMSF amplitudes, we also considered normalized RMSF profiles (the RMSFN profiles), defined by eq 9, in part of the analysis and for visualization purposes.
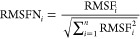
9

To determine the dependence of the *r*_p_ and *r*_s_ correlation coefficients on the method used (UNRES-flex, UNRES-DSSP-flex, CABS-flex, and NOLB) and on the type of secondary structures (α, β, and α + β), a two-way analysis of variance (ANOVA)^49^ was carried out at the 0.05 significance level, using the online server available at https://www.statskingdom.com/two-way-anova-calculator/.

## 3 Results

### 3.1 Impact of Terminal Sections on Fluctuation Profiles and Dependence on the Trajectory

**Figure 1:**
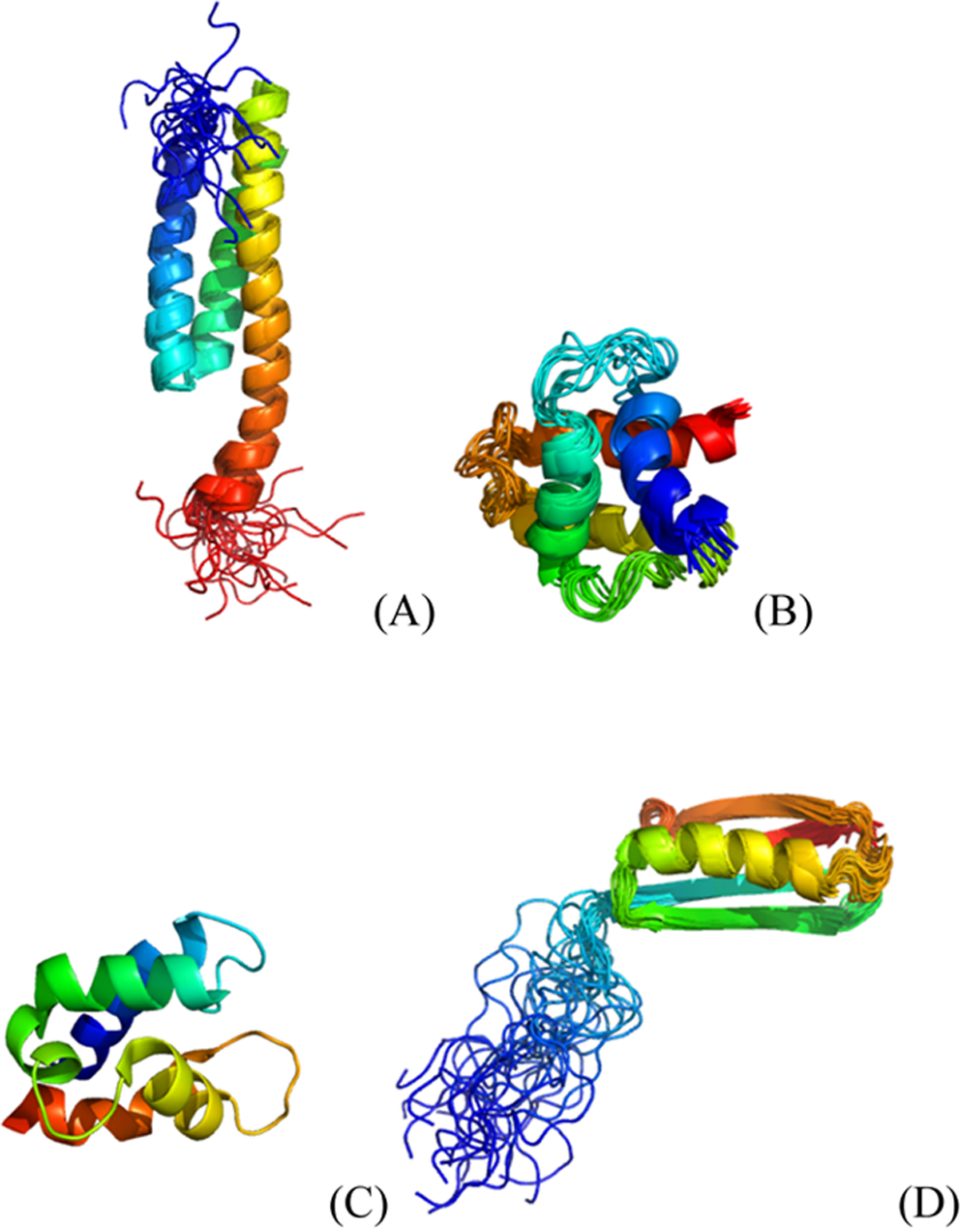
Cartoon representations of the structures of proteins selected for detailed discussion. (A) Nuclear receptor binding factor 2 from mice (PDB: 2CRB, all-α, NMR structure), (B) Gag polyprotein of the Rous sarcoma virus (PDB: 1A6S, all-α, NMR structure),^50^ (C) vitamin D-dependent calcium-binding protein from the bovine intestine (PDB: 3ICB, an all-α, the X-ray structure),^51^ and (D) third SH3 domain of the Cin85 adapter protein (PDB: 2K9G, all-β, NMR structure).

**Figure 2:**
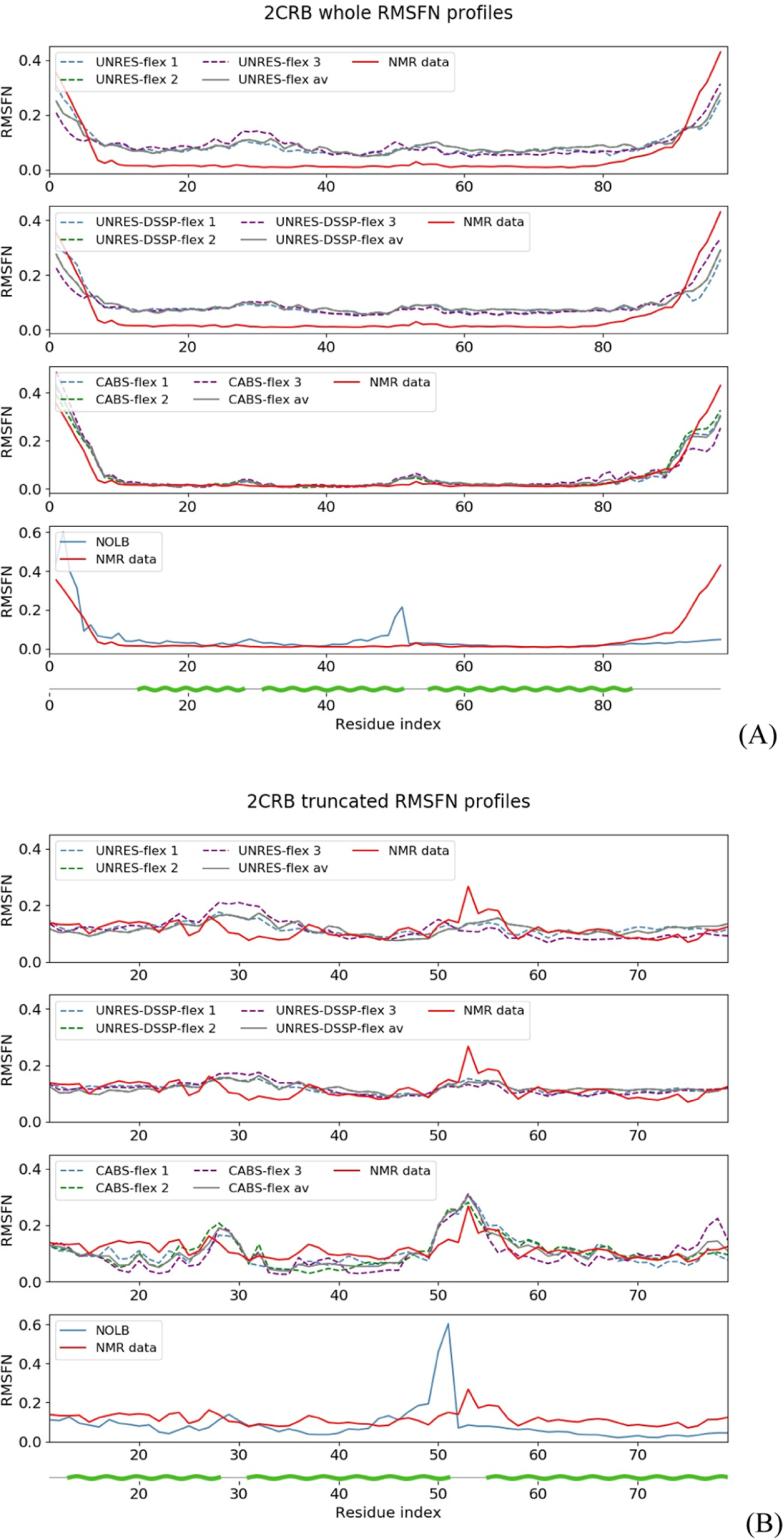
RMSFN profiles for 2CRB calculated with UNRES-flex, UNRES-DSSP-flex, CABS-flex (three simulations and average), and NOLB compared with that calculated from the NMR ensemble for (A) whole and (B) truncated (to remove the terminal segments with high fluctuations) structures. The profiles are distinguished with line styles and colors as described in the graphs. The green wave lines at the bottom of the graphs mark the α-helical segments. For the whole structures, the *r*_p_ (*r*_s_) values corresponding to the first, second, and third simulations and the mean *r*_p_ (*r*_s_) values were 0.93 (0.61), 0.94 (0.58), 0.85 (0.56), and 0.94 (0.58) for UNRES-flex, 0.89 (0.71), 0.95 (0.62), 0.95 (0.68), and 0.95 (0.62) for UNRES-DSSP-flex, 0.94 (0.87), 0.96 (0.78), 0.87 (0.75), and 0.94 (0.81) for CABS-flex, and 0.56 (0.53) for NOLB. For the truncated structures, the *r*_p_ (*r*_s_) values corresponding to the first, second, and third simulations and the mean *r*_p_ (*r*_s_) values were 0.43 (0.60), 0.34 (0.27), 0.35 (0.43), and 0.44 (0.56) for UNRES-flex, 0.47 (0.53), 0.27 (0.21), 0.52 (0.51), and 0.47 (0.52) for UNRES-DSSP-flex, 0.74 (0.68), 0.60 (0.43), 0.53 (0.33), and 0.66 (0.50) for CABS-flex, and 0.39 (0.49) for NOLB.

As stated in Section 2.5, the fluctuations of the N- and C-terminal regions are usually much higher than those of other protein fragments. To illustrate this observation, let us consider the nuclear receptor binding factor 2 from mice (PDB: 2CRB, an all-α protein), the NMR structure of which is shown in Figure 1A. For the whole protein, UNRES-flex, UNRES-DSSP-flex, and CABS-flex yield high *r*_p_ (from 0.94 to 0.96; Figure 2A). The correlation coefficients were computed from RMSFN profiles averaged over all three trajectories corresponding to a given method. However, it is clearly seen from Figure 2A that the good agreement between the experimental RMSFN profile and those predicted with UNRES-flex, UNRES-DSSP-flex, and CABS-flex arises from the fluctuations at the ends (which usually are not biologically relevant). After removing the terminal sections (which leaves residues 8–85), the agreement between the experimental and predicted RMSFN profiles is still good with *r*_p_ ranging from 0.49 to 0.81 (Figure 2B). Consequently, to avoid biasing the results, we analyze fluctuation profiles without the terminal sections.

To determine how the fluctuation profiles depend on the trajectory, we compared the RMSFN profiles obtained from each of the three individual MD or MC trajectories simulated with UNRES (UNRES-DSSP) or CABS. It should be noted that NOLB is a deterministic method, and thus, only one calculation per system was required. From Figure 2A, it can be seen that the differences between the RMSFN profiles calculated from individual trajectories are similar over the whole sequence. Therefore, at the ends, where the fluctuations are high, these differences are smaller compared to the extent of fluctuations. Consequently, there are only small differences between the Δ*r*_p_ (0.09 for UNRES-flex, 0.06 for UNRES-DSSP-flex, and 0.08 for CABS-flex) and Δ*r*_s_ (0.06 for UNRES-flex, 0.12 for UNRES-DSSP-flex, and 0.02 for CABS-flex) values corresponding to different trajectories. Consequently, based on the analysis of whole RMSFN profiles, it could be concluded that running just one trajectory was sufficient. However, it must be kept in mind that the complete RMSFN profiles are dominated by the fluctuations of end sections. When these sections are removed to keep only biologically relevant regions, the differences between RMSFN profiles become more noticeable (Figure 2B), which is reflected in bigger differences in the Δ*r*_p_ (0.38 for UNRES-flex, 0.22 for UNRES-DSSP-flex, and 0.19 for CABS-flex) and Δ*r*_s_ (0.05 for UNRES-flex, 0.22 for UNRES-DSSP-flex, and 0.03 for CABS-flex) values. This result clearly demonstrates that running multiple trajectories is necessary to get reliable RMSFN profiles. Consequently, in what follows, we discuss the RMSFN profiles and the quantities derived from those averaged over three trajectories.

It should also be noted that the *r*_p_ values for the experimental fluctuation profiles and those predicted with NOLB are lower, this feature being probably due to the fact that normal mode analysis is mostly relevant to cases with a well-defined reference structure, which is not the case of NMR ensembles.

### 3.2 Comparison of Predicted and Experimental RMSFN Profiles over the Benchmark Set

#### 3.2.1 Dependence on the Prediction Method, Method of Structure Determination, and Secondary Structure

The RMSFN plots for all 100 benchmark proteins are collected in Figures S1 (truncated structures) and S2 (full structures) of the Supporting Information. The correlation coefficients for each individual protein are shown in Tables S2 and S3 of the Supporting Information for the truncated and the full structures, respectively.

**Figure 3:**
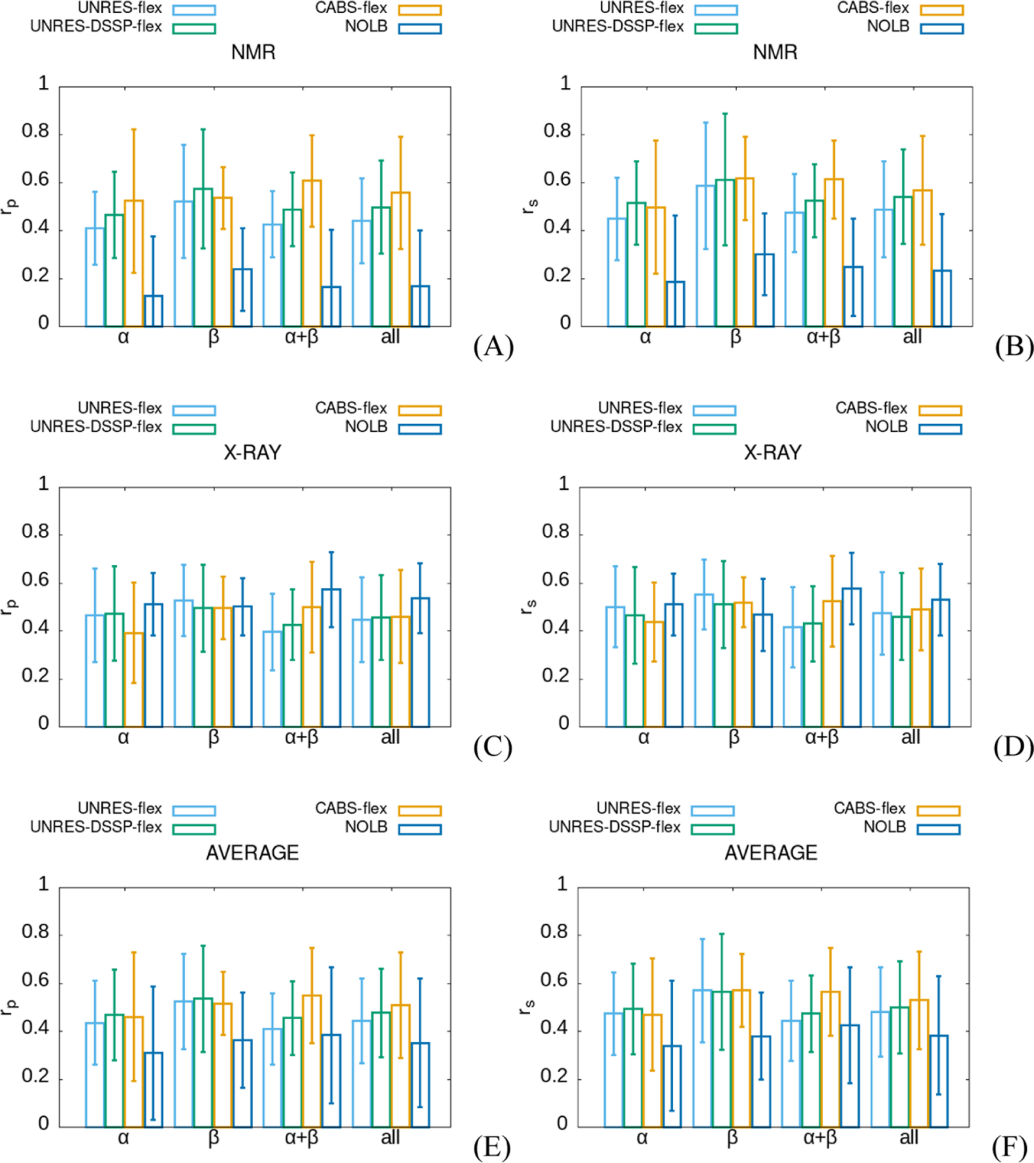
Whiskered bar plots (whiskers corresponding to standard deviations) of the mean Pearson (A, C, E) and Spearman (B, D, F) correlation coefficients between residue fluctuation profiles obtained by UNRES-flex (steel blue), UNRES-DSSP-flex (green), CABS-flex (orange) simulations, and NOLB (blue) for truncated structures, for NMR (A, B) and X-ray (C, D) structures and irrespective of the structure determination method (E, F). Analyses were performed for the α-, β- and α + β-proteins and irrespective of the secondary structure type (all), as indicated in the abscissae.

To determine which method considered in this work (UNRES-flex, UNRES-DSSP-flex, CABS-flex, or NOLB) best reproduces the fluctuation profiles, averages of the Pearson (*r*_p_) and Spearman (*r*_s_) coefficient were computed first for the truncated and full structures, respectively. In each instance, the averages were computed over the subsets corresponding to a given method of protein structure determination (NMR or X-ray) and a given type of secondary structure (α, β, or α + β) of the subset of the set of 100 proteins considered in this work. These average coefficients are summarized in Tables S4 and S5 of the Supporting Information for the truncated and full structures, respectively, and shown as whiskered bar plots (showing their mean values and their standard deviations) in Figure 3A–3D. For completeness, averages over the structure determination method, secondary structure type, and both are also shown in Tables S4 and S5 and in Figure 3E,3F. As can be seen from Figure 3 and Tables S4 and S5, the correlation coefficients (*r*_p_ and *r*_s_) seem to depend mostly on the type of the method for fluctuation prediction.

To verify the above qualitative observation, we used two-way ANOVA with the following two categories of variables: (i) methods for fluctuation prediction and (ii) type of secondary structures. Separate analyses were carried out depending on the method of structure determination. The reason for this separation was that for X-ray structures, the experimental fluctuation profiles are calculated from the *B*-factors (eq 6) and correspond to harmonic or quasi-harmonic vibrations around the energy minimum. Conversely, the experimental fluctuation profiles calculated from the NMR structures (eq 5) have the sense of ensemble variance around the mean structure. The respective significance levels are summarized in Table S6 of the Supporting Information. As can be seen, the dependence of the correlation coefficients on the method of fluctuation prediction is significant at least at the 0.05 significance level (except for the *r*_s_ and X-ray structures), while that on the secondary structure type is insignificant (except for *r*_s_ and NMR structures). The influence of both categories of variables (interaction) on the correlation coefficients is of little or no significance (Table S6). Thus, ANOVA confirms the dependence of *r*_s_ and *r*_p_ on the method of fluctuation prediction that could be seen from Figure 3.

To determine the specific differences between the qualities of the methods of fluctuation prediction when applied to proteins with a given type of secondary structure, we compared the respective sets of correlation coefficients (*r*_p_ or *r*_s_) by using Student’s test, separately for the X-ray and for the NMR structures. Detailed results are collected in Table S7 of the Supporting Information. As can be seen, CABS-flex, UNRES-flex, and UNRES-DSSP-flex perform better than NOLB for NMR structures, while NOLB performs better than UNRES-flex and UNRES-DSSP-flex for X-ray structures of α + β proteins (Table S7C,D). It should be noted that we only refer to the differences that have been assessed to be statistically significant. Irrespective of the secondary structure type, NOLB performs better than UNRES-flex but only in terms of the difference of the Pearson coefficient (Table S7C).

CABS-flex performs better, at the 5% or better statistical significance, than UNRES-flex for NMR structures of α + β proteins (Table S7A of the Supporting Information). For the X-ray structures of α + β proteins, CABS-flex performs better than UNRES-flex; however, the statistical significance of the differences between the correlation coefficients is worse than 5% (Table S7C of the Supporting Information). For the NMR structures of α + β proteins, the Pearson correlation coefficient corresponding to CABS-flex is greater than that for UNRES-DSSP-flex at about 5% significance level (Table S7A). On the other hand, for the benchmark proteins irrespective of the secondary structure type, there are no statistically significant differences between CABS-flex, UNRES-flex, and UNRES-DSSP-flex. Consequently, it can be stated that CABS-flex and UNRES-DSSP-flex predict fluctuations with a similar accuracy.

**Figure 4:**
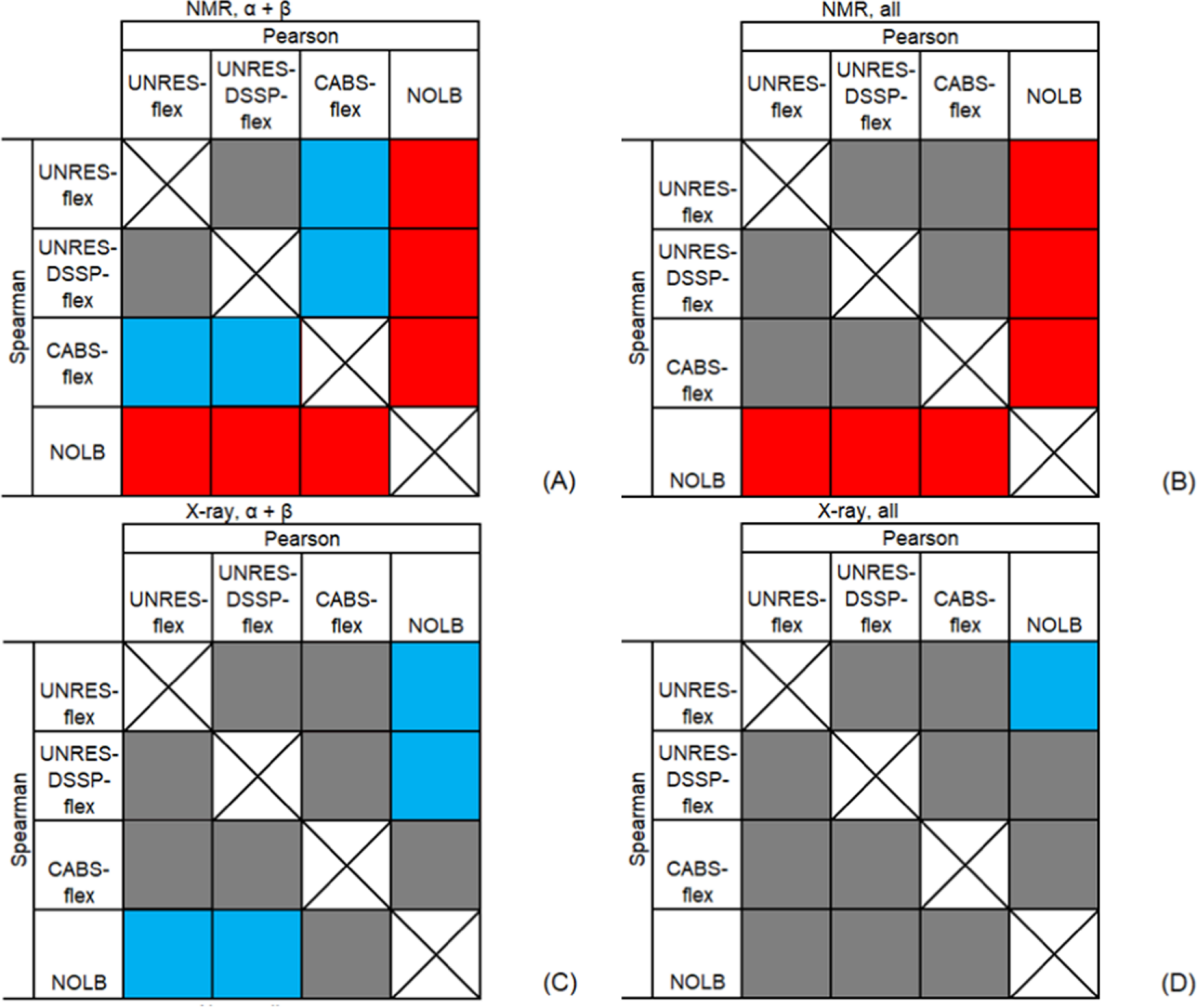
Visualization of the sign of and statistical significance of the *r*_p_ (above-diagonal) and *r*_s_ (below-diagonal) correlation coefficients corresponding to the four methods of fluctuation prediction evaluated in this work for NMR (A, B) and X-ray (C, D) structures and α + β (A, C) and all (B, D) proteins. For *r*_p_, the method corresponding to the respective column heading is compared with that corresponding to that of an above-diagonal row entry, while for *r*_s_, the method corresponding to the respective row heading is compared with that of a below-diagonal column entry. Red: the difference is negative at <0.05 significance level, blue: the difference is positive at <0.05 significance level, and gray: the difference is statistically insignificant.

The above observations are illustrated in Figure 4A–D, drawn for α + β (A and C) and all (B and D) secondary structure types and NMR (A and B) and X-ray (C and D) structures, in which we plotted arrays with fields corresponding to the *r*_p_ (above-diagonal) and *r*_s_ (below-diagonal), the colors of the respective fields indicating statistical significance and the sign of the difference.

#### 3.2.2 Distributions of Correlation Coefficients

The analysis described in Section 3.2.1 enabled us to evaluate the four prediction methods considered in this study with regard to their average performance, depending on the secondary structure type and fluctuation prediction method. However, the shape of the distribution of a correlation coefficient, in particular its modality and asymmetry, can provide additional information regarding the likelihood of very good or very poor predictions.

**Figure 5:**
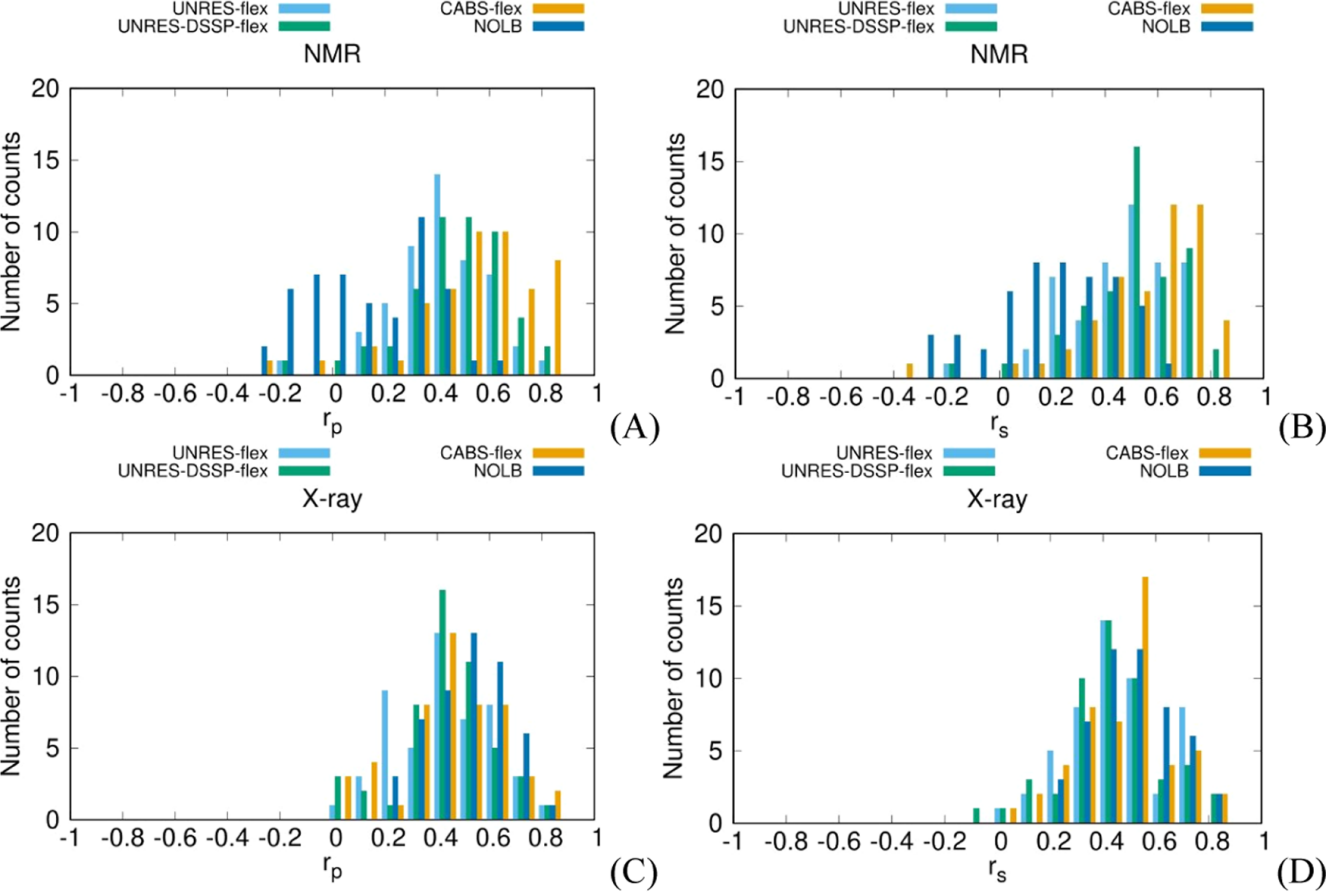
Distributions of Pearson’s (*r*_p_) or Spearman’s (*r*_s_) correlation coefficient values between the fluctuation profiles obtained from the NMR ensembles or X-ray B-factors and those predicted by using UNRES-flex (steel-blue column), UNRES-DSSP-flex (green column), CABS-flex (orange column), and NOLB (blue column) after removing the terminal protein sections.

To analyze the asymmetry of the distributions, we binned the Pearson and Spearman correlation coefficients (*r*_p_ or *r*_s_, respectively, 0.1 bin size), separately for NMR and X-ray structures, and plotted the numbers of counts against the respective correlation coefficients (Figure 5A–D). This analysis was performed for truncated structures only.

An apparent feature of the *r*_p_ distribution calculated with NOLB for NMR structures is its bimodality, with the first maximum at about 0 and the second one at about 0.3. The *r*_s_ distribution is effectively unimodal; however, it is very broad, which could be a result of merging two lobes. If only the second part of the *r*_p_ distribution is considered, the performance of NOLB (as assessed by *r*_p_) is similar to that of UNRES-flex, while the first part corresponds to poor predictions. We, therefore, examined the structures corresponding to the first part of the distribution (centered at *r*_p_ ≈ 0). This list is shown in Table S8 of the Supporting Information. However, the respective structures do not seem to possess any common feature such as exceptional noncompactness, a particular type of secondary structure, particularly long loops, etc. Therefore, it seems that the poorer performance of NOLB with NMR structures compared to that with X-ray structures could result from its Hamiltonian, which is based on interatomic distances exclusively.

The well-established elements of protein X-ray structures (e.g., α-helices) are usually both close to the other structural element of that protein, and if they are on the protein exterior, they are tightly packed against the other protein molecules. On the other hand, loops are both more distant from the rest of the protein and are not tightly packed against the other protein molecules. Therefore, the flexibility of a fragment in an X-ray structure primarily depends on the distance of its atoms from those of the other fragments, the strength of specific interactions being less important. This observation is supported by Figure 5C,D, in which the distributions of *r*_p_ and *r*_s_, respectively, from X-ray structures are shown. As can be seen, the distributions corresponding to NOLB are unimodal and slightly shifted to the right with respect to those from the other methods. The NMR structures selected for this study are those of monomeric proteins in solution and, consequently, the strength of specific interactions is more important. Consequently, NOLB could probably benefit from weighting the harmonic Hamiltonian elements by the contact energies between the respective residues taken, e.g., from the Miyazawa–Jernigan table.^52^

While the *r*_p_ and *r*_s_ distributions corresponding to NMR structures and UNRES-flex, UNRES-DSSP-flex, and CABS-flex do not exhibit apparent bimodality, they are all left-skewed, this indicating that poor predictions can occasionally happen. For quantitative comparison, we computed the skewnesses of each distribution, which is defined by eq 10
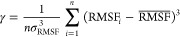
10where *n* is the number of structures analyzed. For *r*_p_, the values are γ_UNRES-flex_ = −0.50, γ_UNRES-DSSP-flex_ = −0.86, γ_CABS-flex_ = −1.16, and γ_NOLB_ = −0.01, while for *r*_s_, γ_UNRES-flex_ = −0.79, γ_UNRES-DSSP-flex_ = −1.09, γ_CABS-flex_ = −1.60, and γ_NOLB_ = −0.34. The skewness is the most negative for CABS-flex; this observation conforms to the high density of the significantly positive correlation coefficient and the presence of those with small values and even with negative values. The second negative skewness occurs for UNRES-DSSP-flex, the respective distributions having similar features. For UNRES-flex, the distribution is more symmetric because its center is shifted to the left compared to CABS-flex and UNRES-DSSP-flex. For NOLB, the skewness is negligible for *r*_p_ and still small for *r*_s_, which results from a similar weight of both lobes of *r*_p_ distribution and the nearly symmetric broad distribution of *r*_s_. From this analysis, it can be concluded that while CABS-flex and UNRES-DSSP-flex generally give good fluctuation predictions for NMR ensembles (and, thereby, protein ensembles in solution), they can occasionally result in poor predictions. The IDs of the proteins for which low correlation coefficients were obtained are collected in Table S8 of the Supporting Information. These structures do not seem to exhibit any particular features, and therefore, UNRES or CABS Hamiltonians could be responsible for poor performance. This observation is supported by the fact that some of these structures are common for all of the three methods.

For X-ray structures, the *r*_p_ and *r*_s_ distributions are not significantly skewed (Figure 5C,D). The skewness values are γ_UNRES-flex_ = −0.02, γ_UNRES-DSSP-flex_ = −0.46, γ_CABS-flex_ = −0.31, and γ_NOLB_ = −0.14 for *r*_p_ and γ_UNRES-flex_ = −0.06, γ_UNRES-DSSP-flex_ = −0.21, γ_CABS-flex_ = −0.21, and γ_NOLB_ = 0.13 for *r*_s_. It can also be noted that the maxima of the distributions for CABS-flex, UNRES-DSSP-flex, and UNRES-flex are shifted to the left compared to those corresponding to NMR structures (as opposed to the distributions from NOLB). This feature can result from predicting fluctuations for isolated protein molecules, while they are subjected to crystal packing in the crystal structures. As mentioned, NOLB has an advantage here because crystal packing could be, in part, accounted for by the harmonic Hamiltonian dependent on contact distances.

**Figure 6:**
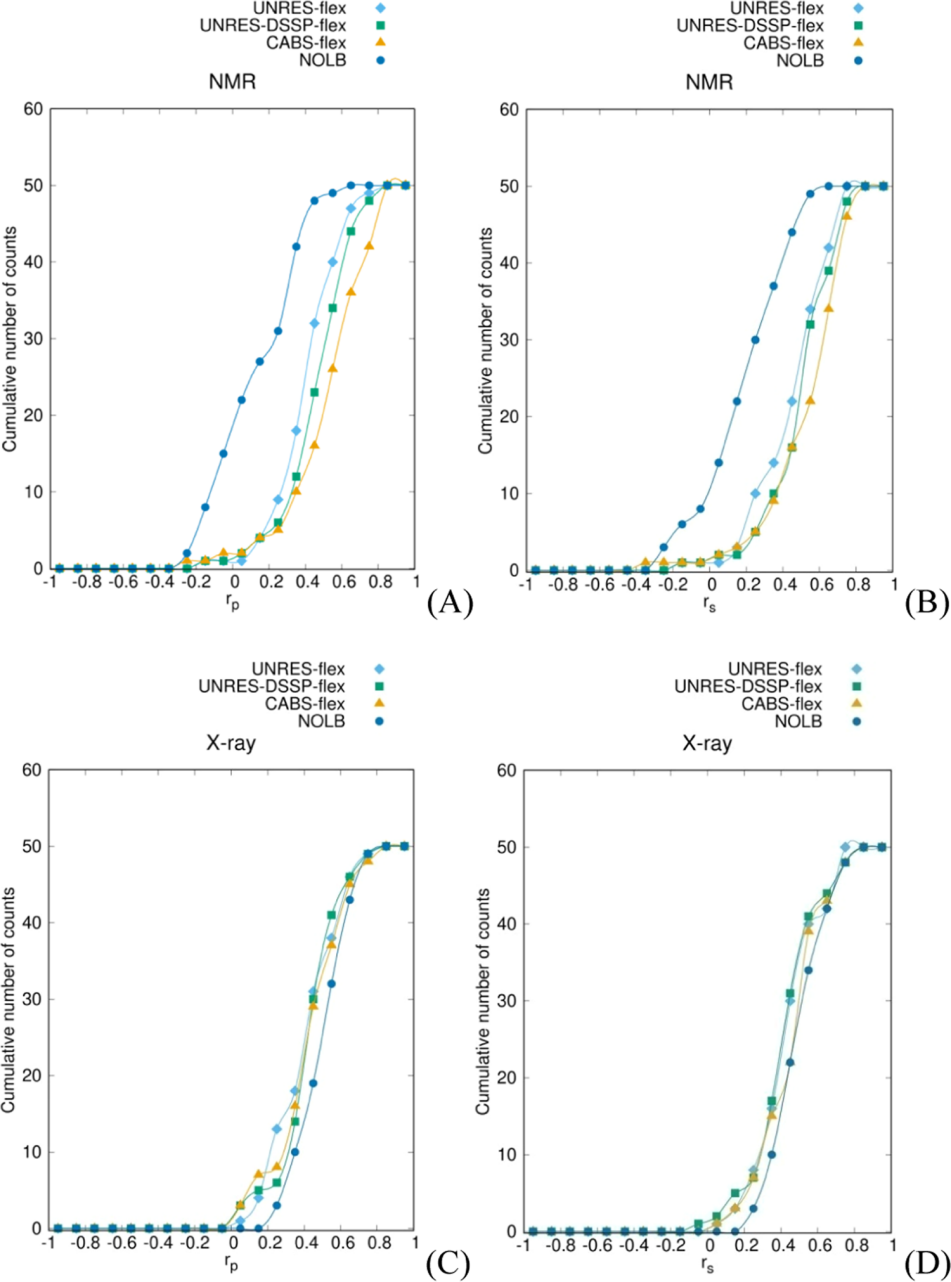
The cumulative distribution functions of the average Pearson’s (*r*_p_) or Spearman’s (*r*_s_) correlation coefficient values between the fluctuation profiles obtained from the NMR ensembles or X-ray B-factors and those predicted byUNRES-flex (steel-blue line and diamonds), UNRES-DSSP-flex (green line and squares), CABS-flex (orange line and triangles), and NOLB (blue line and dots) after removing the terminal protein sections.

Because the distributions of *r*_p_ and *r*_s_ are multimodal or skewed, the quality of prediction methods cannot be assessed based on the comparison of averages (carried out in Section 3.2.1) alone. Therefore, we constructed the cumulative distribution plots shown in Figure 6A–D. The value of the cumulative distribution at *x* is defined as the number of structures such that the respective correlation coefficient does not exceed *x*. As can be seen from Figure 6A,B, for NMR structures, the curves corresponding to NOLB are significantly shifted to the left from those corresponding to the other three methods, this indicating that NOLB is not the preferable method for predicting the fluctuations of NMR structures (and, thereby, single protein molecules in solution). This conclusion fully conforms with that drawn in Section 3.2.1. For the other three methods, the rank is UNRES-flex < UNRES-DSSP-flex < CABS-flex, suggesting that CABS-flex performs best (however, as assessed in Section 3.2.1, the difference is statistically significant only between UNRES-flex and CABS-flex; Figure 4). Thus, CABS-flex and UNRES-DSSP-flex seem to be preferable to predict the fluctuation profiles of proteins in solution.

For the X-ray structures, the curves corresponding to NOLB are shifted to the right with respect to those corresponding to the other three methods, the difference being, however, small. This observation conforms with the respective conclusion drawn in Section 3.2.1 because NOLB was found statistically better only for X-ray structures of α + β proteins (Figure 4C). On the other hand, it can be seen from Figure 6 that the lowest correlation coefficients from NOLB start from about 0.2 for X-ray structures, while they start from 0 for the other three methods. This observation suggests that NOLB should be the method of choice for X-ray structures. Further to this conclusion, NOLB is probably the best method to predict the fluctuation profiles of proteins in a crowded environment.

#### 3.2.3 Dependence of Correlation Coefficients on Protein Size

To check whether the quality of protein flexibility prediction depends on chain length, we plotted the average values of *r*_p_ and *r*_s_ in the number of residues in a chain for truncated structures for each of the four methods, separately for the NMR and the X-ray structures. These plots are shown in Figure S3 of the Supporting Information. The chain lengths ranged from 20 to 117 residues for the NMR and from 30 to 532 residues for X-ray structures (after truncation). As can be seen from the figure, no correlation is exhibited between chain length and *r*_p_ or *r*_s_. However, for all methods except UNRES-flex, the correlation coefficients are less dispersed and concentrated around 0.5 for chains exceeding 200 residues, this feature being the most pronounced for CABS-flex.

### 3.3 Detailed Analysis of RMSFN Profiles for Representative Proteins

**Figure 7:**
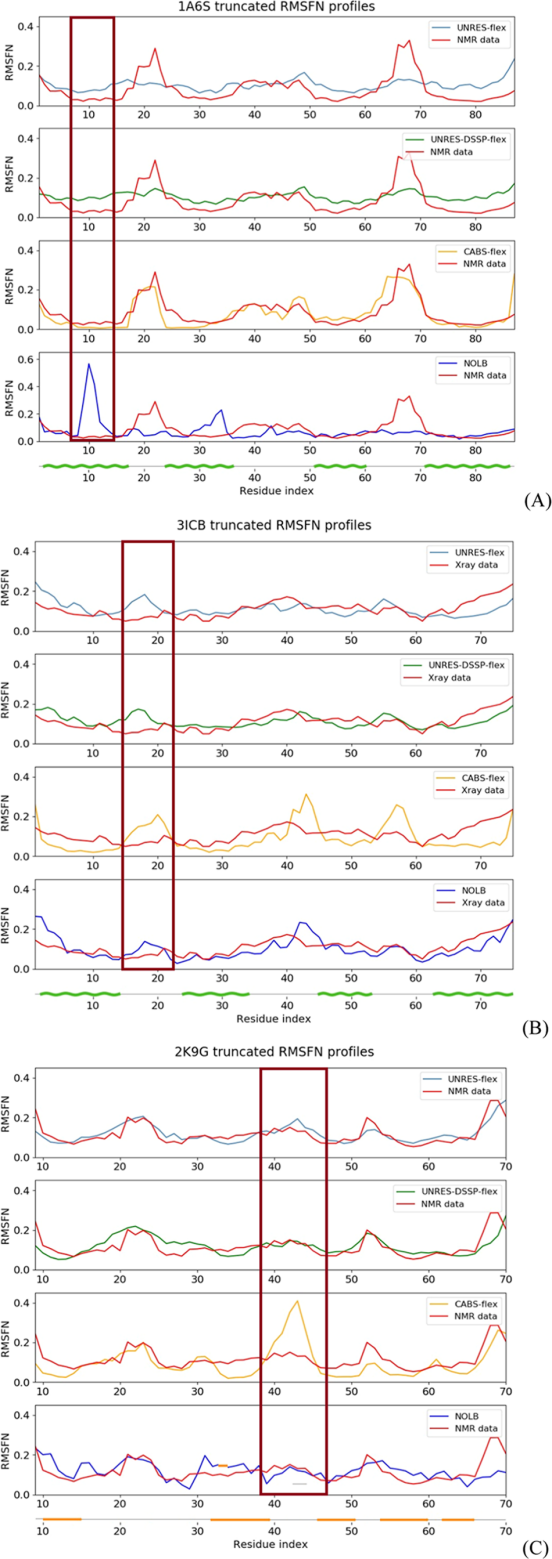
Experimental (X-ray or NMR; red lines) and calculated by UNRES-flex (light blue line), UNRES-DSSP-flex (green line), and CABS-FLEX (yellow line). NOLB (blue line) RMSFN profiles for the truncated structures of proteins with PDB IDs 1A6S, 3ICB, and 2K9G proteins. The profiles from UNRES-flex, UNRES-DSSP-flex, and CABS-flex averaged over three independent simulations. The secondary structure is indicated below the graphs corresponding to each of the proteins with a wave-shaped green line (α-helix) or a straight orange line (β-strand). Frames have been put around the regions in which different extents of fluctuations are predicted by different methods (see the text). The Pearson and Spearman coefficients for the respective proteins are as follows: 1A6S: *r*_p_ = 0.34 and *r*_s_ = 0.52 (UNRES-flex), *r*_p_ = 0.56 and *r*_s_ = 0.53 (UNRES-DSSP-flex), *r*_p_ = 0.81 and *r*_s_ = 0.75 (CABS-flex), *r*_p_ = −0.08 and *r*_s_ = 0.17 (NOLB); 3ICB: (UNRES-flex: *r*_p_ = 0.13 and *r*_s_ = 0.15, UNRES-DSSP-flex: *r*_p_ = 0.30 and *r*_s_ = 0.31, CABS-flex: *r*_p_ = 0.19 and *r*_s_ = 0.33 and NOLB: *r*_p_ = 0.56 and *r*_s_ = 0.68), and 2K9G: (UNRES-flex: *r*_p_ = 0.80 and *r*_s_ = 0.78, UNRES-DSSP-flex: *r*_p_ = 0.61 and *r*_s_ = 0.61, CABS-flex: *r*_p_ = 0.52 and *r*_s_ = 0.71 and NOLB: *r*_p_ = 0.31 and *r*_s_ = 0.39).

To illustrate the differences between the performance of the four methods for fluctuation prediction, we selected three representative cases: Gag polyprotein of the Rous sarcoma virus (PDB: 1A6S, an all-α, the NMR structure),^50^ vitamin D-dependent calcium-binding protein from the bovine intestine (PDB: 3ICB, an all-α, the X-ray structure),^51^ and the third SH3 domain of the Cin85 adapter protein (PDB: 2K9G, an all-β, the NMR structure). The structures of these three proteins are shown in Figure 1B–D, and their fluctuation profiles are shown in Figure 7A–C.

The first example (Figure 7A) is the NMR structure of an α-helical protein. Its RMSFN profile calculated with NOLB differs considerably from that determined from the NMR ensemble. The respective *r*_p_ and *r*_s_ correlation coefficients are low, which places this case among those of the left lobe in the NOLB *r*_p_ distribution of the left upper panel of Figure 5C,D. The reason for this is the presence of a RMSFN maximum in the N-terminal part of the NOLB profile, which is not present in that from the NMR ensemble. Conversely, the maximum present in the C-terminal section of the NMR RMSFMN profile is absent in the NOLB profile. The profiles from UNRES-flex, UNRES-DSSP-flex, and CABS-flex are confluent with that from NMR ensemble, the CABS-flex profile being in better agreement with the NMR ensemble profile owing to more pronounced differences between the maxima and the background.

The second example (Figure 7B) is the X-ray structure of an α-helical protein. In this case, the NOLB RMSFN profile conforms better with that calculated from the *B*-factors, which is reflected in the correlation coefficients. The reason for this is that UNRES-flex, UNRES-DSSP-flex, and CABS-flex predict increased fluctuations around residue 18, where the *B*-factors are low. Additionally, CABS-flex exaggerates the extent of fluctuations in the middle of the chain.

Generally, CABS-flex has a tendency to predict focused fluctuation regions, while these regions are predicted as diffuse by UNRES-flex and UNRES-DSSP-flex. In most cases, this feature of CABS-flex is beneficial, but it can also lead to poor predictions. An example is shown in Figure 7C, in which the RMSFN profiles of a β-protein, NMR structure, are shown. CABS-flex exaggerated the fluctuations in the middle of the chain, which has resulted in very poor correlation between the respective RMSFN profiles and those from the NMR ensemble, as opposed to the other methods.

## 4 Discussion and Conclusions

UNRES, CABS-flex, and NOLB are methods used for fluctuations prediction and analysis, but they differ in their approaches to predicting protein flexibility. UNRES^25^ is a physics-based method, and CABS-flex uses a knowledge-based coarse-grained force field, while NOLB is based on the elastic network concept. CABS-flex^34^ is designed to predict protein flexibility and understand their function. In contrast, NOLB^17^ is designed to predict the motions by normal modes corresponding to the biologically relevant motions and the most likely flexibility of a protein based on experimental data. In this work, we evaluated the ability of each of these four methods to predict protein fluctuations depending on the source of a structure (X-ray or NMR ensemble) and secondary structure class (α, β, or α + β).

Because we found that, particularly for NMR structures, the fluctuation profiles determined for the whole structures are dominated by outstandingly high fluctuations at the ends (as illustrated in Figure 2A), which are usually biologically irrelevant, the fluctuation profile analysis was carried out for the truncated structure, from which these terminal regions were removed. For X-ray structures, the experimental fluctuation profiles were calculated from the *B*-factors (eq 6), while for the NMR structures, the profiles were calculated from NMR ensembles deposited in the PDB (eq 5). Except for NOLB, which is an analytical method, the predicted fluctuation profiles were calculated as averages over three independent MC (CABS-flex) or MD (UNRES-flex and UNRES-DSSP-flex) simulations. Since the primary concern is the similarity of the predicted and experimental fluctuation profiles irrespective of the fluctuation magnitude, we selected the Pearson (*r*_p_; eq 7) and Spearman (*r*_s_; eq 8) correlation coefficients as descriptors.

For the X-ray structures, NOLB gives the best fluctuation predictions, this feature being clearly manifested in the respective cumulative distribution plots of the *r*_p_ and *r*_s_ coefficients in Figure 6C,D, in which the curves corresponding to NOLB are most shifted to the right. The difference in both correlation coefficients from NOLB is statistically significant with respect to those from UNRES-flex and UNRES-DSSP-flex for α + β proteins and in the Pearson coefficient from NOLB with respect to that from UNRES-flex irrespective of the secondary structure. This feature of NOLB probably results from its elastic network basis because the freedom of a protein molecule in a crystal is effectively confined to the neighborhood of a local energy minimum. Moreover, the simple elastic network Hamiltonian with the force constant dependent on distance is a good approximation to the energy surface around the structure because of tight crystal packing. The other three methods assume that a protein molecule (or oligomer) is in solution and, consequently, is not restricted in motion. This situation corresponds to the conditions of NMR experiments. It should also be noted that the variant of the UNRES force field used in this work was calibrated with the ensembles of protein structures determined by NMR.^25^

For NMR structures, the ranking of the magnitude of the correlation coefficients on average is CABS-flex > UNRES-DSSP-flex > UNRES-flex > NOLB, as seen from the respective cumulative distribution plots in Figure 6A,B, the difference between NOLB and the other three methods being statistically significant (Figure 4C,D). As mentioned, this difference probably results from the fact that proteins in solution are not confined and, consequently, the simple elastic network Hamiltonian that does not differentiate the character of interactions (which depend on residue hydrophobicity in the first place). The difference of the correlation coefficients from CABS-flex and those from UNRES-flex is statistically significant for α + β proteins but not for proteins irrespective of the structural class (Figure 4A). The difference between CABS-flex and UNRES-DSSP-flex is not statistically significant except for that of the *r*_p_ coefficient and α + β proteins, which exhibits weak statistical significance (Figure 4A). It should be noted that CABS-flex and UNRES-DSSP-flex implement restraints on the geometry of the elements with a well-defined secondary structure; consequently, it can be concluded that including such restraints is beneficial with regard to fluctuation prediction. The better performance of CABS-flex could result from less aggressive coarse graining of CABS (four centers) compared to UNRES (two centers). Even though only Cα atoms are considered in quantifying fluctuations, the presence of a greater number of centers indirectly influences the results. Moreover, the representation of interactions becomes more accurate (at the expense of increased computation cost) as more centers are included in a model. The smaller number of interaction sites in UNRES is compensated by a more refined representation of interactions in UNRES, which included nonspherical side chain–side chain potentials, more elaborate representation of local interactions, and the presence of more kinds of terms that couple backbone local and backbone hydrogen bonding interactions.^25^

In summary, the best agreement of NOLB fluctuation profiles with the X-ray *B*-factors suggests that it is the method of choice for predicting the fluctuation profiles of proteins in a crowded environment, both with regard to accuracy and to speed. Conversely, for proteins in solution, which are best represented by NMR ensembles, it is advisable to run both CABS-flex and UNRES-DSSP-flex. CABS-flex gives overall better agreement between the calculated and experimental fluctuation profiles but happens to predict high fluctuations in regions where they are low (see Figure 7C as an example). On the other hand, UNRES has been parallelized,^22^ including the application in the UNRES server that runs UNRES-DSSP-flex.^22,32^ In single-processor mode, the recently optimized UNRES code, which is implemented in the current version of the UNRES server,^31^ appears to be faster than CABS. For the 71-residue 1VIG protein, the computations with CABS-flex required 169 wall-clock seconds, as compared to 95 wall-clock seconds for UNRES-DSSP-flex (both programs were run on the same Intel i5-4570, 3.2 GHz node); with 4 cores, the UNRES-DSSP-flex required 45 wall-clock seconds. It should be noted that 200,000 conformations are generated by UNRES-flex and UNRES-DSSP-flex, as opposed to the 100,000 conformations for CABS-flex. Therefore, UNRES-DSSP-flex seems to be preferable for bigger proteins (for which the computations take full advantage of parallelization) because of speed. Details of the performance and scalability of the optimized parallel implementation of UNRES, in comparison with the coarse-grained (MARTINI^53^ implemented in GROMACS^55^) and all-atom approaches (AMBER^54^ and AMBER implemented in GROMACS^55^), can be found in our recent work.^22^

## Supporting Information Available

List of the structures of benchmark proteins, Pearson (*r*_p_) and Spearman (*r*_s_) correlation coefficients of the RMSF profiles over the truncated structures, the Pearson (*r*_p_) and Spearman (*r*_s_) correlation coefficients of the RMSF profiles over the full structures, average Pearson’s (*r*_p_) and Spearman’s correlation coefficients (*r*_s_) between fluctuation profiles calculated for truncated structures, average Pearson’s (*r*_p_) and Spearman’s correlation coefficients (*r*_s_) between fluctuation profiles calculated for full structures, ANOVA results of Pearson’s (*r*_p_) and Spearman’s (*r*_s_) correlation coefficients between the fluctuation profiles for truncated structures, significance of differences of Pearson’s (*r*_p_) and Spearman’s (*r*_s_) correlation coefficients of fluctuation profiles, corresponding to truncated structures by the two-sample *t* test, NMR structures with low similarity of the fluctuation profiles estimated from NMR ensembles, normalized fluctuation profiles (RMSFN, dimensionless) for truncated structures, normalized fluctuation profiles (RMSFN, dimensionless) for full structures, and scatter plots of the Pearson (*r*_p_) and Spearman (*r*_s_) coefficients between the experimental and predicted fluctuation profiles in chain length (PDF)
