# Supplementary material for: Assessment of Four Theoretical Approaches to Predict Protein Flexibility in the Crystal Phase and Solution
Source: J Chem Theory Comput. 2024 Aug 22;20(17):7667–81. doi: 10.1021/acs.jctc.4c00754 (PMC11391579; doi:10.1021/acs.jctc.4c00754)
Supplement: Supplementary file 1 — ct4c00754_si_001.pdf [file ct4c00754_si_001.pdf]

**Table S1.** A list of the structures of benchmark proteins selected for this study with the respective numbers of residues in the sequence (#res), the numbers of residues after truncation (#res.$_t$), and the ranges of residues after truncation (range$_t$). The entries are grouped according to secondary-structure class. PDB codes in boldface font correspond to NMR structures, those in regular font correspond to X-ray structures.

| **$\alpha$ proteins** | | | | **$\beta$ proteins** | | | | **$\alpha$+$\beta$ proteins** | | | |
|---|---|---|---|---|---|---|---|---|---|---|---|
| **PDB ID[a]** | **# res.** | **#res.$_t$** | **range$_t$** | **PDB ID[a]** | **# res.** | **#res.$_t$** | **range$_t$** | **PDB ID[a]** | **# res.** | **#res.$_t$** | **range$_t$** |
| **1A6S** | 87 | 87 | 1-87 | **1AH9** | 71 | 63 | 5-67 | 1A80 | 277 | 276 | 2-277 |
| **1ACP** | 77 | 71 | 3-73 | 1BK2 | 57 | 55 | 2-56 | 1ADG | 374 | 373 | 2-374 |
| 1ALA | 316 | 316 | 1-316 | 1CSP | 67 | 67 | 1-67 | 1AKY | 218 | 216 | 2-217 |
| 1AUM | 70 | 70 | 1-70 | **1ED7** | 45 | 45 | 1-45 | 1CFJ | 532 | 530 | 1-530 |
| **1BAL** | 51 | 33 | 16-48 | **1HRF** | 67 | 45 | 7-51 | **1CLB** | 75 | 72 | 2-73 |
| 1ENH | 54 | 48 | 4-51 | **1IYV** | 79 | 71 | 3-73 | 1CTF | 68 | 68 | 1-68 |
| **1EO0** | 77 | 73 | 2-73 | 1MJC | 69 | 69 | 1-69 | **1E0G** | 48 | 46 | 2-47 |
| **1FEX** | 59 | 54 | 5-58 | 1NOA | 113 | 113 | 1-113 | 1EM7 | 56 | 56 | 1-56 |
| **1GAB** | 53 | 44 | 9-52 | 1RUW | 69 | 69 | 1-69 | **1G6E** | 87 | 86 | 2-87 |

| | | | | | | | | | | | |
|---|---|---|---|---|---|---|---|---|---|---|---|
| **1HNS** | 47 | 39 | 6-44 | 1TEN | 89 | 89 | 1-89 | **1GHH** | 81 | 80 | 1-79 |
| 1HYP | 75 | 73 | 1-73 | **1TPN** | 50 | 43 | 5-47 | **1HYW** | 58 | 52 | 3-52 |
| **1IYR** | 83 | 72 | 4-75 | **1WIU** | 93 | 93 | 1-93 | 1IG5 | 75 | 75 | 1-75 |
| **1J7O** | 76 | 71 | 5-75 | **1WKT** | 88 | 85 | 4-88 | **1IQO** | 88 | 87 | 2-88 |
| 1K40 | 126 | 124 | 3-126 | 1XCD | 305 | 305 | 1-305 | **1K8B** | 52 | 51 | 1-51 |
| **1L2Y** | 20 | 18 | 2-19 | **2K9G** | 73 | 62 | 9-70 | **1LEB** | 72 | 66 | 4-69 |
| **1LQ7** | 67 | 65 | 3-67 | **2KYW** | 87 | 78 | 2-79 | 1OGQ | 313 | 310 | 3-312 |
| **1P68** | 102 | 98 | 4-101 | **2LGN** | 66 | 65 | 2-66 | 1OIX | 168 | 168 | 1-168 |
| **1POU** | 71 | 66 | 5-70 | **2LVC** | 90 | 87 | 4-90 | 1OPD | 85 | 81 | 1-81 |
| **1PRV** | 56 | 36 | 5-40 | 3PUC | 98 | 97 | 1-97 | 1PGA | 56 | 56 | 1-56 |
| **1RIJ** | 23 | 17 | 5-21 | 4F98 | 62 | 60 | 3-62 | 1PHT | 83 | 83 | 1-83 |
| 1ROP | 56 | 56 | 1-56 | 4M9O | 97 | 95 | 1-95 | 1PTF | 87 | 86 | 1-86 |

| | | | | | | | | | | | |
|---|---|---|---|---|---|---|---|---|---|---|---|
| 1YRF | 35 | 34 | 1-34 | | | | | 1QRE | 210 | 208 | 3-210 |
| 2BF9 | 36 | 34 | 1-34 | | | | | **1STU** | 68 | 66 | 1-66 |
| **2CRB** | 97 | 69 | 11-79 | | | | | 1THX | 108 | 104 | 3-106 |
| **2E7N** | 117 | 108 | 10-117 | | | | | 1TIG | 88 | 87 | 1-87 |
| **2HEP** | 42 | 23 | 8-30 | | | | | 1UBQ | 76 | 71 | 1-71 |
| **2HI3** | 73 | 47 | 9-55 | | | | | **1VIG** | 71 | 67 | 4-70 |
| 2YGS | 92 | 91 | 1-91 | | | | | 2ACY | 98 | 93 | 5-97 |
| 3ICB | 75 | 74 | 1-75 | | | | | **2BBY** | 69 | 62 | 5-66 |
| 3WFW | 138 | 134 | 1-134 | | | | | **2FMR** | 65 | 64 | 2-65 |
| | | | | | | | | **2L09** | 62 | 48 | 3-50 |
| | | | | | | | | 2LZM | 164 | 162 | 1-162 |
| | | | | | | | | **2M6Q** | 91 | 82 | 2-83 |

| | | | | | | | | | | | |
|---|---|---|---|---|---|---|---|---|---|---|---|
| | | | | | | | | **2MQ8** | 112 | 97 | 1-97 |
| | | | | | | | | **2N2U** | 77 | 63 | 3-65 |
| | | | | | | | | **2PTL** | 78 | 59 | 19-77 |
| | | | | | | | | **2RGF** | 97 | 97 | 1-97 |
| | | | | | | | | **3CI2** | 64 | 63 | 2-64 |
| | | | | | | | | 3E8Y | 30 | 29 | 1-29 |
| | | | | | | | | 3KYW | 54 | 53 | 1-53 |
| | | | | | | | | 3KYY | 54 | 53 | 1-53 |
| | | | | | | | | **3NCM** | 92 | 92 | 1-92 |
| | | | | | | | | 4N6T | 79 | 79 | 1-79 |
| | | | | | | | | 4OZU | 390 | 389 | 2-390 |
| | | | | | | | | 4QRL | 110 | 110 | 1-110 |

| | | | | | | | | | | | |
|---|---|---|---|---|---|---|---|---|---|---|---|
| | | | | | | | | 4R7E | 69 | 69 | 1-69 |
| | | | | | | | | 4RTE | 124 | 123 | 2-124 |
| | | | | | | | | 5D14 | 70 | 68 | 1-68 |
| | | | | | | | | 6DNB | 411 | 409 | 1-410 |

**Table S2.** The Pearson ($r_p$) and Spearman ($r_s$) correlation coefficients of the RMSF profiles over the truncated structures calculated from B-factors (X-ray structures) or NMR ensembles (NMR structures) and those predicted with UNRES-flex, UNRES-DSSP-flex, CABS-flex, and NOLB, respectively.

| # | PDB ID | UNRES-flex | | UNRES-DSSP-flex | | CABS-flex | | NOLB | |
|---|---|---|---|---|---|---|---|---|---|
| | | $r_s$ | $r_p$ | $r_s$ | $r_p$ | $r_s$ | $r_p$ | $r_s$ | $r_p$ |
| 1 | 1A6S | 0.52 | 0.34 | 0.59 | 0.56 | 0.75 | 0.81 | 0.17 | -0.08 |
| 2 | 1A80 | 0.42 | 0.40 | 0.49 | 0.44 | 0.59 | 0.50 | 0.67 | 0.66 |
| 3 | 1ACP | 0.22 | 0.20 | 0.46 | 0.35 | 0.42 | 0.38 | 0.22 | 0.22 |
| 4 | 1ADG | 0.31 | 0.30 | 0.36 | 0.31 | 0.45 | 0.41 | 0.45 | 0.40 |
| 5 | 1AH9 | 0.71 | 0.65 | 0.74 | 0.72 | 0.79 | 0.68 | 0.21 | 0.21 |
| 6 | 1AKY | 0.40 | 0.38 | 0.44 | 0.58 | 0.52 | 0.73 | 0.71 | 0.73 |
| 7 | 1ALA | 0.47 | 0.43 | 0.58 | 0.54 | 0.52 | 0.49 | 0.55 | 0.48 |
| 8 | 1AUM | 0.40 | 0.24 | 0.52 | 0.43 | 0.28 | 0.11 | 0.44 | 0.46 |

| | | | | | | | | | |
|---|---|---|---|---|---|---|---|---|---|
| 9 | 1BAL | 0.27 | 0.26 | 0.32 | 0.27 | 0.05 | -0.08 | 0.08 | -0.05 |
| 10 | 1BK2 | 0.36 | 0.22 | 0.14 | 0.05 | 0.37 | 0.22 | 0.47 | 0.54 |
| 11 | 1CFJ | 0.53 | 0.53 | 0.56 | 0.59 | 0.37 | 0.40 | 0.68 | 0.67 |
| 12 | 1CLB | 0.33 | 0.30 | 0.56 | 0.58 | 0.81 | 0.88 | 0.09 | -0.08 |
| 13 | 1CSP | 0.41 | 0.49 | 0.50 | 0.51 | 0.50 | 0.45 | 0.28 | 0.28 |
| 14 | 1CTF | 0.29 | 0.27 | 0.38 | 0.36 | 0.10 | 0.05 | 0.41 | 0.40 |
| 15 | 1E0G | 0.42 | 0.62 | 0.58 | 0.66 | 0.61 | 0.73 | 0.28 | 0.14 |
| 16 | 1ED7 | 0.49 | 0.45 | 0.59 | 0.53 | 0.33 | 0.22 | 0.00 | -0.02 |
| 17 | 1EM7 | 0.31 | 0.28 | 0.46 | 0.42 | 0.52 | 0.53 | 0.69 | 0.61 |
| 18 | 1ENH | 0.52 | 0.62 | 0.39 | 0.51 | 0.48 | 0.51 | 0.35 | 0.34 |
| 19 | 1EO0 | 0.29 | 0.36 | 0.33 | 0.41 | 0.31 | 0.37 | 0.30 | 0.34 |
| 20 | 1FEX | 0.50 | 0.39 | 0.64 | 0.46 | 0.54 | 0.40 | -0.13 | -0.21 |
| 21 | 1G6E | 0.59 | 0.43 | 0.69 | 0.53 | 0.46 | 0.41 | 0.48 | 0.33 |
| 22 | 1GAB | 0.20 | 0.16 | 0.22 | 0.18 | 0.39 | 0.38 | 0.57 | 0.46 |
| 23 | 1GHH | 0.28 | 0.35 | 0.40 | 0.52 | 0.21 | 0.36 | 0.07 | 0.09 |
| 24 | 1HNS | 0.53 | 0.49 | 0.67 | 0.66 | 0.81 | 0.81 | 0.16 | 0.16 |
| 25 | 1HRF | -0.18 | -0.12 | -0.18 | -0.12 | 0.25 | 0.43 | 0.45 | 0.45 |
| 26 | 1HYP | 0.72 | 0.62 | 0.62 | 0.54 | 0.69 | 0.54 | 0.60 | 0.57 |
| 27 | 1HYW | 0.60 | 0.57 | 0.58 | 0.56 | 0.64 | 0.60 | -0.03 | -0.02 |
| 28 | 1IG5 | 0.77 | 0.62 | 0.78 | 0.75 | 0.75 | 0.61 | 0.76 | 0.74 |
| 29 | 1IQO | 0.41 | 0.32 | 0.56 | 0.44 | 0.62 | 0.50 | 0.18 | 0.06 |

| | | | | | | | | | |
|---|---|---|---|---|---|---|---|---|---|
| 30 | 1IYR | 0.21 | 0.53 | 0.52 | 0.61 | 0.69 | 0.74 | 0.44 | 0.07 |
| 31 | 1IYV | 0.47 | 0.42 | 0.47 | 0.42 | 0.63 | 0.67 | 0.12 | 0.33 |
| 32 | 1J7O | 0.17 | 0.11 | 0.09 | 0.05 | -0.37 | -0.29 | 0.23 | 0.04 |
| 33 | 1K40 | 0.78 | 0.78 | 0.82 | 0.85 | 0.68 | 0.64 | 0.55 | 0.55 |
| 34 | 1K8B | 0.34 | 0.31 | 0.46 | 0.43 | 0.16 | 0.14 | 0.17 | -0.01 |
| 35 | 1L2Y | 0.56 | 0.57 | 0.72 | 0.62 | 0.70 | 0.54 | -0.23 | -0.14 |
| 36 | 1LEB | 0.22 | 0.23 | 0.35 | 0.32 | 0.86 | 0.82 | -0.05 | -0.12 |
| 37 | 1LQ7 | 0.66 | 0.66 | 0.67 | 0.60 | 0.69 | 0.81 | 0.67 | 0.49 |
| 38 | 1MJC | 0.76 | 0.80 | 0.63 | 0.69 | 0.58 | 0.67 | 0.60 | 0.65 |
| 39 | 1NOA | 0.45 | 0.43 | 0.36 | 0.38 | 0.42 | 0.40 | 0.65 | 0.66 |
| 40 | 1OGQ | 0.08 | 0.06 | 0.20 | 0.17 | 0.62 | 0.59 | 0.45 | 0.50 |
| 41 | 1OIX | 0.25 | 0.25 | 0.49 | 0.49 | 0.21 | 0.32 | 0.51 | 0.54 |
| 42 | 1OPD | 0.55 | 0.52 | 0.57 | 0.62 | 0.35 | 0.40 | 0.43 | 0.40 |
| 43 | 1P68 | 0.61 | 0.51 | 0.58 | 0.56 | 0.69 | 0.56 | 0.47 | 0.34 |
| 44 | 1PGA | 0.21 | 0.18 | 0.16 | 0.13 | 0.31 | 0.14 | 0.30 | 0.20 |
| 45 | 1PHT | 0.79 | 0.77 | 0.83 | 0.70 | 0.72 | 0.67 | 0.82 | 0.78 |
| 46 | 1POU | 0.32 | 0.24 | 0.54 | 0.47 | 0.49 | 0.65 | -0.22 | -0.15 |
| 47 | 1PRV | 0.63 | 0.53 | 0.69 | 0.54 | 0.49 | 0.60 | 0.52 | 0.60 |
| 48 | 1PTF | 0.29 | 0.29 | 0.41 | 0.42 | 0.51 | 0.39 | 0.52 | 0.60 |
| 49 | 1QRE | 0.77 | 0.71 | 0.74 | 0.71 | 0.71 | 0.76 | 0.80 | 0.83 |
| 50 | 1RIJ | 0.46 | 0.48 | 0.74 | 0.78 | 0.71 | 0.68 | -0.28 | -0.19 |

| | | | | | | | | | |
|---|---|---|---|---|---|---|---|---|---|
| 51 | 1ROP | 0.53 | 0.52 | 0.50 | 0.59 | 0.43 | 0.54 | 0.49 | 0.55 |
| 52 | 1RUW | 0.39 | 0.49 | 0.35 | 0.47 | 0.36 | 0.62 | 0.29 | 0.48 |
| 53 | 1STU | 0.46 | 0.48 | 0.40 | 0.48 | 0.55 | 0.88 | 0.38 | 0.17 |
| 54 | 1TEN | 0.48 | 0.48 | 0.44 | 0.46 | 0.56 | 0.61 | 0.29 | 0.35 |
| 55 | 1THX | 0.37 | 0.32 | 0.36 | 0.38 | 0.58 | 0.46 | 0.48 | 0.45 |
| 56 | 1TIG | 0.40 | 0.42 | 0.47 | 0.48 | 0.64 | 0.49 | 0.72 | 0.61 |
| 57 | 1TPN | 0.57 | 0.70 | 0.76 | 0.87 | 0.49 | 0.58 | 0.14 | 0.02 |
| 58 | 1UBQ | 0.49 | 0.49 | 0.47 | 0.40 | 0.52 | 0.50 | 0.65 | 0.67 |
| 59 | 1VIG | 0.79 | 0.66 | 0.27 | 0.33 | 0.85 | 0.86 | 0.53 | 0.48 |
| 60 | 1WIU | 0.70 | 0.48 | 0.77 | 0.66 | 0.67 | 0.47 | 0.59 | 0.45 |
| 61 | 1WKT | 0.66 | 0.47 | 0.80 | 0.59 | 0.72 | 0.55 | 0.48 | 0.37 |
| 62 | 1XCD | 0.71 | 0.64 | 0.74 | 0.70 | 0.58 | 0.49 | 0.31 | 0.41 |
| 63 | 1YRF | 0.58 | 0.69 | 0.35 | 0.60 | 0.58 | 0.62 | 0.32 | 0.45 |
| 64 | 2ACY | 0.33 | 0.27 | 0.27 | 0.29 | 0.26 | 0.33 | 0.44 | 0.47 |
| 65 | 2BBY | 0.55 | 0.62 | 0.75 | 0.82 | 0.51 | 0.76 | 0.38 | 0.48 |
| 66 | 2BF9 | 0.29 | 0.21 | -0.06 | 0.02 | 0.27 | 0.06 | 0.34 | 0.33 |
| 67 | 2CRB | 0.56 | 0.44 | 0.52 | 0.47 | 0.50 | 0.66 | 0.49 | 0.39 |
| 68 | 2E7N | 0.61 | 0.57 | 0.69 | 0.67 | 0.72 | 0.83 | 0.09 | 0.06 |
| 69 | 2FMR | 0.63 | 0.58 | 0.83 | 0.78 | 0.77 | 0.60 | 0.42 | 0.33 |
| 70 | 2HEP | 0.71 | 0.61 | 0.51 | 0.43 | 0.58 | 0.73 | 0.14 | 0.38 |
| 71 | 2HI3 | 0.63 | 0.45 | 0.34 | 0.19 | 0.64 | 0.75 | -0.14 | -0.14 |

| | | | | | | | | | |
|---|---|---|---|---|---|---|---|---|---|
| 72 | 2K9G | 0.78 | 0.80 | 0.61 | 0.61 | 0.71 | 0.52 | 0.39 | 0.31 |
| 73 | 2KYW | 0.75 | 0.63 | 0.78 | 0.69 | 0.72 | 0.67 | 0.19 | -0.02 |
| 74 | 2L09 | 0.43 | 0.36 | 0.29 | 0.20 | 0.44 | 0.68 | 0.00 | -0.18 |
| 75 | 2LGN | 0.72 | 0.53 | 0.56 | 0.57 | 0.77 | 0.61 | 0.37 | 0.36 |
| 76 | 2LVC | 0.79 | 0.73 | 0.85 | 0.77 | 0.71 | 0.49 | 0.36 | 0.16 |
| 77 | 2LZM | 0.37 | 0.46 | 0.23 | 0.33 | 0.46 | 0.49 | 0.49 | 0.55 |
| 78 | 2M6Q | 0.45 | 0.45 | 0.43 | 0.50 | 0.58 | 0.55 | 0.25 | 0.03 |
| 79 | 2MQ8 | 0.59 | 0.49 | 0.73 | 0.62 | 0.76 | 0.58 | 0.28 | 0.22 |
| 80 | 2N2U | 0.14 | 0.17 | 0.43 | 0.41 | 0.55 | 0.48 | 0.39 | 0.37 |
| 81 | 2PTL | 0.56 | 0.43 | 0.52 | 0.41 | 0.72 | 0.72 | 0.53 | 0.68 |
| 82 | 2RGF | 0.55 | 0.37 | 0.51 | 0.32 | 0.40 | 0.19 | 0.29 | 0.18 |
| 83 | 2YGS | 0.16 | 0.15 | 0.07 | 0.05 | 0.11 | 0.01 | 0.33 | 0.27 |
| 84 | 3CI2 | 0.32 | 0.23 | 0.52 | 0.40 | 0.65 | 0.62 | -0.16 | -0.24 |
| 85 | 3E8Y | 0.54 | 0.30 | 0.48 | 0.43 | 0.18 | 0.12 | 0.55 | 0.52 |
| 86 | 3ICB | 0.15 | 0.13 | 0.31 | 0.30 | 0.33 | 0.19 | 0.68 | 0.56 |
| 87 | 3KYW | 0.51 | 0.51 | 0.49 | 0.45 | 0.49 | 0.44 | 0.47 | 0.56 |
| 88 | 3KYY | 0.49 | 0.41 | 0.44 | 0.41 | 0.52 | 0.36 | 0.53 | 0.63 |
| 89 | 3NCM | 0.71 | 0.44 | 0.57 | 0.39 | 0.68 | 0.34 | 0.39 | 0.24 |
| 90 | 3PUC | 0.57 | 0.47 | 0.67 | 0.53 | 0.60 | 0.46 | 0.57 | 0.47 |
| 91 | 3WFW | 0.55 | 0.62 | 0.55 | 0.64 | 0.38 | 0.50 | 0.43 | 0.54 |
| 92 | 4F98 | 0.75 | 0.66 | 0.73 | 0.68 | 0.71 | 0.64 | 0.58 | 0.62 |

| | | | | | | | | | |
|---|---|---|---|---|---|---|---|---|---|
| 93 | 4M9O | 0.64 | 0.60 | 0.55 | 0.51 | 0.52 | 0.43 | 0.64 | 0.55 |
| 94 | 4N6T | 0.41 | 0.34 | 0.53 | 0.52 | 0.84 | 0.73 | 0.76 | 0.79 |
| 95 | 4OZU | 0.49 | 0.48 | 0.31 | 0.36 | 0.44 | 0.48 | 0.61 | 0.63 |
| 96 | 4QRL | 0.69 | 0.61 | 0.47 | 0.46 | 0.81 | 0.81 | 0.79 | 0.78 |
| 97 | 4R7E | 0.46 | 0.47 | 0.44 | 0.46 | 0.59 | 0.55 | 0.37 | 0.34 |
| 98 | 4RTE | 0.49 | 0.52 | 0.38 | 0.30 | 0.74 | 0.81 | 0.71 | 0.74 |
| 99 | 5D14 | 0.56 | 0.56 | 0.46 | 0.44 | 0.35 | 0.40 | 0.52 | 0.53 |
| 100 | 6DNB | 0.46 | 0.34 | 0.58 | 0.41 | 0.51 | 0.37 | 0.58 | 0.36 |
| Average: | | 0.48 | 0.44 | 0.44 | 0.48 | 0.53 | 0.51 | 0.38 | 0.35 |

**Table S3.** The Pearson ($r_p$) and Spearman ($r_s$) correlation coefficients of the RMSF profiles over the full structures calculated from B-factors (X-ray structures) or NMR ensembles (NMR structures) and those predicted with UNRES-flex, UNRES-DSSP-flex, CABS-flex, and NOLB, respectively.

| # | PDB ID | UNRES-flex | | UNRES-DSSP-flex | | CABS-flex | | NOLB | |
|---|---|---|---|---|---|---|---|---|---|
| | | $r_s$ | $r_p$ | $r_s$ | $r_p$ | $r_s$ | $r_p$ | $r_s$ | $r_p$ |
| 1 | 1A6S | 0.52 | 0.34 | 0.59 | 0.56 | 0.75 | 0.81 | 0.17 | -0.08 |
| 2 | 1A80 | 0.42 | 0.39 | 0.49 | 0.42 | 0.59 | 0.51 | 0.67 | 0.64 |
| 3 | 1ACP | 0.38 | 0.59 | 0.55 | 0.67 | 0.36 | 0.30 | 0.25 | 0.18 |
| 4 | 1ADG | 0.32 | 0.33 | 0.37 | 0.34 | 0.45 | 0.41 | 0.46 | 0.41 |
| 5 | 1AH9 | 0.77 | 0.84 | 0.79 | 0.82 | 0.83 | 0.79 | 0.19 | 0.31 |
| 6 | 1AKY | 0.42 | 0.42 | 0.45 | 0.62 | 0.53 | 0.76 | 0.71 | 0.72 |
| 7 | 1ALA | 0.47 | 0.43 | 0.58 | 0.54 | 0.52 | 0.49 | 0.55 | 0.48 |

| | | | | | | | | | |
|---|---|---|---|---|---|---|---|---|---|
| 8 | 1AUM | 0.40 | 0.24 | 0.52 | 0.43 | 0.28 | 0.11 | 0.44 | 0.46 |
| 9 | 1BAL | 0.71 | 0.94 | 0.77 | 0.96 | 0.47 | 0.68 | 0.73 | 0.81 |
| 10 | 1BK2 | 0.42 | 0.39 | 0.21 | 0.20 | 0.39 | 0.21 | 0.52 | 0.60 |
| 11 | 1CFJ | 0.54 | 0.56 | 0.56 | 0.61 | 0.38 | 0.41 | 0.69 | 0.69 |
| 12 | 1CLB | 0.40 | 0.46 | 0.60 | 0.64 | 0.82 | 0.87 | 0.12 | -0.07 |
| 13 | 1CSP | 0.41 | 0.49 | 0.50 | 0.51 | 0.50 | 0.45 | 0.28 | 0.28 |
| 14 | 1CTF | 0.29 | 0.27 | 0.38 | 0.36 | 0.10 | 0.05 | 0.41 | 0.40 |
| 15 | 1E0G | 0.49 | 0.78 | 0.63 | 0.77 | 0.65 | 0.84 | 0.33 | 0.25 |
| 16 | 1ED7 | 0.49 | 0.45 | 0.59 | 0.53 | 0.33 | 0.22 | 0.00 | -0.02 |
| 17 | 1EM7 | 0.31 | 0.28 | 0.46 | 0.42 | 0.52 | 0.53 | 0.69 | 0.61 |
| 18 | 1ENH | 0.66 | 0.91 | 0.57 | 0.89 | 0.62 | 0.83 | 0.54 | 0.76 |
| 19 | 1EO0 | 0.40 | 0.59 | 0.43 | 0.66 | 0.36 | 0.31 | 0.38 | 0.39 |
| 20 | 1FEX | 0.62 | 0.88 | 0.73 | 0.88 | 0.64 | 0.79 | 0.07 | 0.57 |
| 21 | 1G6E | 0.60 | 0.49 | 0.70 | 0.57 | 0.47 | 0.44 | 0.49 | 0.40 |
| 22 | 1GAB | 0.52 | 0.88 | 0.54 | 0.92 | 0.64 | 0.95 | 0.71 | 0.84 |
| 23 | 1GHH | 0.30 | 0.52 | 0.42 | 0.67 | 0.24 | 0.57 | 0.10 | 0.16 |
| 24 | 1HNS | 0.72 | 0.82 | 0.81 | 0.93 | 0.86 | 0.84 | 0.41 | 0.62 |
| 25 | 1HRF | 0.55 | 0.87 | 0.55 | 0.87 | 0.69 | 0.79 | 0.39 | 0.05 |
| 26 | 1HYP | 0.74 | 0.72 | 0.65 | 0.68 | 0.71 | 0.63 | 0.61 | 0.54 |
| 27 | 1HYW | 0.71 | 0.76 | 0.70 | 0.82 | 0.73 | 0.81 | 0.19 | 0.46 |
| 28 | 1IG5 | 0.77 | 0.62 | 0.78 | 0.75 | 0.75 | 0.61 | 0.76 | 0.74 |

| | | | | | | | | | |
|---|---|---|---|---|---|---|---|---|---|
| 29 | 1IQO | 0.43 | 0.41 | 0.57 | 0.50 | 0.63 | 0.53 | 0.21 | 0.29 |
| 30 | 1IYR | 0.47 | 0.79 | 0.68 | 0.91 | 0.79 | 0.91 | 0.53 | 0.22 |
| 31 | 1IYV | 0.55 | 0.80 | 0.55 | 0.80 | 0.65 | 0.86 | 0.24 | 0.07 |
| 32 | 1J7O | 0.33 | 0.79 | 0.25 | 0.83 | -0.15 | 0.52 | 0.37 | 0.67 |
| 33 | 1K40 | 0.79 | 0.82 | 0.82 | 0.85 | 0.68 | 0.59 | 0.57 | 0.55 |
| 34 | 1K8B | 0.37 | 0.41 | 0.49 | 0.65 | 0.20 | 0.37 | 0.19 | 0.08 |
| 35 | 1L2Y | 0.68 | 0.84 | 0.77 | 0.60 | 0.76 | 0.58 | -0.12 | -0.01 |
| 36 | 1LEB | 0.51 | 0.85 | 0.62 | 0.88 | 0.89 | 0.86 | 0.07 | 0.48 |
| 37 | 1LQ7 | 0.69 | 0.77 | 0.70 | 0.78 | 0.72 | 0.72 | 0.69 | 0.92 |
| 38 | 1MJC | 0.76 | 0.80 | 0.63 | 0.69 | 0.58 | 0.67 | 0.60 | 0.65 |
| 39 | 1NOA | 0.45 | 0.43 | 0.36 | 0.38 | 0.42 | 0.40 | 0.65 | 0.66 |
| 40 | 1OGQ | 0.08 | 0.06 | 0.19 | 0.16 | 0.63 | 0.62 | 0.46 | 0.56 |
| 41 | 1OIX | 0.25 | 0.25 | 0.49 | 0.49 | 0.21 | 0.32 | 0.51 | 0.54 |
| 42 | 1OPD | 0.60 | 0.60 | 0.62 | 0.70 | 0.35 | 0.32 | 0.45 | 0.33 |
| 43 | 1P68 | 0.65 | 0.67 | 0.63 | 0.72 | 0.72 | 0.64 | 0.48 | 0.42 |
| 44 | 1PGA | 0.21 | 0.18 | 0.16 | 0.13 | 0.31 | 0.14 | 0.30 | 0.20 |
| 45 | 1PHT | 0.79 | 0.77 | 0.83 | 0.70 | 0.72 | 0.67 | 0.82 | 0.78 |
| 46 | 1POU | 0.43 | 0.58 | 0.62 | 0.67 | 0.51 | 0.66 | -0.07 | 0.02 |
| 47 | 1PRV | 0.87 | 0.77 | 0.88 | 0.74 | 0.67 | 0.40 | -0.24 | -0.23 |
| 48 | 1PTF | 0.31 | 0.34 | 0.43 | 0.47 | 0.53 | 0.45 | 0.50 | 0.54 |
| 49 | 1QRE | 0.78 | 0.74 | 0.74 | 0.74 | 0.72 | 0.77 | 0.81 | 0.84 |

| | | | | | | | | | |
|---|---|---|---|---|---|---|---|---|---|
| 50 | 1RIJ | 0.78 | 0.90 | 0.85 | 0.86 | 0.58 | 0.43 | -0.02 | 0.03 |
| 51 | 1ROP | 0.53 | 0.52 | 0.50 | 0.59 | 0.43 | 0.54 | 0.49 | 0.55 |
| 52 | 1RUW | 0.39 | 0.49 | 0.35 | 0.47 | 0.36 | 0.62 | 0.29 | 0.48 |
| 53 | 1STU | 0.48 | 0.49 | 0.44 | 0.49 | 0.58 | 0.88 | 0.40 | 0.18 |
| 54 | 1TEN | 0.48 | 0.48 | 0.44 | 0.46 | 0.56 | 0.61 | 0.29 | 0.35 |
| 55 | 1THX | 0.43 | 0.49 | 0.42 | 0.59 | 0.62 | 0.60 | 0.52 | 0.54 |
| 56 | 1TIG | 0.42 | 0.53 | 0.48 | 0.59 | 0.65 | 0.60 | 0.73 | 0.66 |
| 57 | 1TPN | 0.72 | 0.91 | 0.85 | 0.92 | 0.65 | 0.70 | 0.36 | 0.62 |
| 58 | 1UBQ | 0.58 | 0.77 | 0.53 | 0.64 | 0.59 | 0.75 | 0.71 | 0.85 |
| 59 | 1VIG | 0.82 | 0.88 | 0.38 | 0.82 | 0.87 | 0.88 | 0.58 | 0.78 |
| 60 | 1WIU | 0.70 | 0.48 | 0.77 | 0.66 | 0.67 | 0.47 | 0.59 | 0.45 |
| 61 | 1WKT | 0.69 | 0.63 | 0.82 | 0.75 | 0.73 | 0.54 | 0.50 | 0.52 |
| 62 | 1XCD | 0.71 | 0.64 | 0.74 | 0.70 | 0.58 | 0.49 | 0.31 | 0.41 |
| 63 | 1YRF | 0.61 | 0.78 | 0.40 | 0.73 | 0.62 | 0.77 | 0.35 | 0.41 |
| 64 | 2ACY | 0.42 | 0.67 | 0.38 | 0.78 | 0.35 | 0.63 | 0.52 | 0.60 |
| 65 | 2BBY | 0.67 | 0.72 | 0.82 | 0.77 | 0.56 | 0.54 | 0.45 | 0.60 |
| 66 | 2BF9 | 0.40 | 0.67 | 0.11 | 0.65 | 0.38 | 0.39 | 0.42 | 0.57 |
| 67 | 2CRB | 0.84 | 0.91 | 0.82 | 0.95 | 0.81 | 0.94 | 0.76 | 0.67 |
| 68 | 2E7N | 0.69 | 0.81 | 0.75 | 0.88 | 0.78 | 0.93 | 0.17 | 0.72 |
| 69 | 2FMR | 0.64 | 0.61 | 0.84 | 0.80 | 0.78 | 0.63 | 0.44 | 0.40 |
| 70 | 2HEP | 0.94 | 0.96 | 0.91 | 0.97 | 0.48 | 0.76 | 0.05 | 0.31 |

| | | | | | | | | | |
|---|---|---|---|---|---|---|---|---|---|
| 71 | 2HI3 | 0.89 | 0.93 | 0.81 | 0.92 | 0.84 | 0.90 | 0.14 | 0.06 |
| 72 | 2K9G | 0.86 | 0.77 | 0.76 | 0.74 | 0.80 | 0.51 | 0.53 | 0.73 |
| 73 | 2KYW | 0.82 | 0.85 | 0.84 | 0.96 | 0.79 | 0.70 | 0.30 | 0.01 |
| 74 | 2L09 | 0.73 | 0.95 | 0.67 | 0.86 | 0.66 | 0.91 | 0.25 | 0.02 |
| 75 | 2LGN | 0.73 | 0.66 | 0.58 | 0.59 | 0.78 | 0.63 | 0.40 | 0.50 |
| 76 | 2LVC | 0.81 | 0.84 | 0.86 | 0.88 | 0.71 | 0.40 | 0.42 | 0.69 |
| 77 | 2LZM | 0.39 | 0.53 | 0.26 | 0.44 | 0.48 | 0.52 | 0.50 | 0.58 |
| 78 | 2M6Q | 0.58 | 0.86 | 0.57 | 0.80 | 0.66 | 0.68 | 0.34 | 0.10 |
| 79 | 2MQ8 | 0.73 | 0.98 | 0.83 | 0.99 | 0.84 | 0.96 | 0.37 | 0.20 |
| 80 | 2N2U | 0.51 | 0.96 | 0.65 | 0.98 | 0.67 | 0.90 | 0.41 | 0.03 |
| 81 | 2PTL | 0.80 | 0.95 | 0.78 | 0.94 | 0.85 | 0.85 | 0.74 | 0.81 |
| 82 | 2RGF | 0.55 | 0.37 | 0.51 | 0.32 | 0.40 | 0.19 | 0.29 | 0.18 |
| 83 | 2YGS | 0.18 | 0.21 | 0.10 | 0.18 | 0.14 | 0.12 | 0.35 | 0.30 |
| 84 | 3CI2 | 0.35 | 0.45 | 0.54 | 0.56 | 0.67 | 0.71 | -0.11 | 0.05 |
| 85 | 3E8Y | 0.58 | 0.59 | 0.53 | 0.70 | 0.26 | 0.38 | 0.59 | 0.70 |
| 86 | 3ICB | 0.18 | 0.18 | 0.34 | 0.38 | 0.35 | 0.25 | 0.69 | 0.61 |
| 87 | 3KYW | 0.51 | 0.51 | 0.49 | 0.45 | 0.49 | 0.44 | 0.47 | 0.56 |
| 88 | 3KYY | 0.49 | 0.41 | 0.44 | 0.41 | 0.52 | 0.36 | 0.53 | 0.63 |
| 89 | 3NCM | 0.71 | 0.44 | 0.57 | 0.39 | 0.68 | 0.34 | 0.39 | 0.24 |
| 90 | 3PUC | 0.58 | 0.46 | 0.68 | 0.52 | 0.61 | 0.46 | 0.58 | 0.50 |
| 91 | 3WFW | 0.59 | 0.61 | 0.59 | 0.70 | 0.35 | 0.39 | 0.44 | 0.45 |

| | | | | | | | | | |
|---|---|---|---|---|---|---|---|---|---|
| 92 | 4F98 | 0.77 | 0.67 | 0.76 | 0.73 | 0.73 | 0.76 | 0.61 | 0.63 |
| 93 | 4M9O | 0.66 | 0.67 | 0.58 | 0.59 | 0.55 | 0.47 | 0.66 | 0.60 |
| 94 | 4N6T | 0.41 | 0.34 | 0.53 | 0.52 | 0.84 | 0.73 | 0.76 | 0.79 |
| 95 | 4OZU | 0.50 | 0.49 | 0.32 | 0.38 | 0.45 | 0.51 | 0.61 | 0.62 |
| 96 | 4QRL | 0.69 | 0.61 | 0.47 | 0.46 | 0.81 | 0.81 | 0.79 | 0.78 |
| 97 | 4R7E | 0.46 | 0.47 | 0.44 | 0.46 | 0.59 | 0.55 | 0.37 | 0.34 |
| 98 | 4RTE | 0.51 | 0.56 | 0.39 | 0.38 | 0.74 | 0.82 | 0.71 | 0.73 |
| 99 | 5D14 | 0.60 | 0.78 | 0.51 | 0.70 | 0.38 | 0.45 | 0.56 | 0.59 |
| 100 | 6DNB | 0.47 | 0.41 | 0.59 | 0.47 | 0.52 | 0.42 | 0.59 | 0.41 |
| Average: | | 0.56 | 0.62 | 0.58 | 0.65 | 0.58 | 0.59 | 0.43 | 0.45 |

**Table S4.** The average Pearson's ($r_p$) and Spearman's correlation coefficients ($r_s$) between fluctuation profiles calculated for truncated structures with UNRES-flex, UNRES-DSSP-flex, CABS-flex, and NOLB.

## A: NMR structures

| | Secondary-structure type | | | | | | | |
|---|---|---|---|---|---|---|---|---|
| | **α** | | **β** | | **α +β** | | **all** | |
| | $r_p$ | $r_s$ | $r_p$ | $r_s$ | $r_p$ | $r_s$ | $r_p$ | $r_s$ |
| **UNRES-flex** | 0.410<br>0.153 | 0.449<br>0.172 | 0.522<br>0.236 | 0.586<br>0.264 | 0.427<br>0.138 | 0.474<br>0.163 | 0.441<br>0.176 | 0.489<br>0.200 |
| **UNRES-DSSP-flex** | 0.466<br>0.181 | 0.516<br>0.174 | 0.574<br>0.248 | 0.613<br>0.275 | 0.488<br>0.154 | 0.525<br>0.151 | 0.498<br>0.194 | 0.541<br>0.197 |
| **CABS-flex** | 0.524<br>0.300 | 0.498<br>0.279 | 0.536<br>0.129 | 0.618<br>0.173 | 0.608<br>0.191 | 0.614<br>0.163 | 0.558<br>0.235 | 0.568<br>0.226 |
| **NOLB** | 0.128<br>0.249 | 0.185<br>0.277 | 0.238<br>0.172 | 0.300<br>0.171 | 0.166<br>0.237 | 0.247<br>0.202 | 0.167<br>0.233 | 0.234<br>0.234 |

## B: X-ray structures

| | Secondary-structure type | | | | | | | |
|---|---|---|---|---|---|---|---|---|
| | **α** | | **Β** | | **α +β** | | **all** | |
| | **$r_p$** | **$r_s$** | **$r_p$** | **$r_s$** | **$r_p$** | **$r_s$** | **$r_p$** | **$r_s$** |
| **UNRES-flex** | 0.465<br>0.195 | 0.501<br>0.169 | 0.527<br>0.149 | 0.553<br>0.145 | 0.396<br>0.159 | 0.417<br>0.168 | 0.447<br>0.178 | 0.474<br>0.173 |
| **UNRES-DSSP-flex** | 0.473<br>0.198 | 0.467<br>0.202 | 0.496<br>0.181 | 0.512<br>0.182 | 0.426<br>0.148 | 0.431<br>0.157 | 0.457<br>0.176 | 0.460<br>0.182 |
| **CABS-flex** | 0.392<br>0.209 | 0.439<br>0.165 | 0.497<br>0.131 | 0.519<br>0.104 | 0.499<br>0.189 | 0.524<br>0.190 | 0.460<br>0.194 | 0.492<br>0.171 |
| **NOLB** | 0.512<br>0.130 | 0.512<br>0.129 | 0.502<br>0.120 | 0.468<br>0.150 | 0.573<br>0.158 | 0.578<br>0.150 | 0.537<br>0.145 | 0.532<br>0.149 |

## C: All structures

| | Secondary-structure type | | | | | | | |
|---|---|---|---|---|---|---|---|---|
| | **α** | | **Β** | | **α +β** | | **all** | |
| | **$r_p$** | **$r_s$** | **$r_p$** | **$r_s$** | **$r_p$** | **$r_s$** | **$r_p$** | **$r_s$** |
| **UNRES-flex** | 0.436<br>0.176 | 0.474<br>0.173 | 0.525<br>0.200 | 0.570<br>0.216 | 0.410<br>0.150 | 0.444<br>0.169 | 0.444<br>0.177 | 0.482<br>0.187 |
| **UNRES-DSSP-flex** | 0.469<br>0.189 | 0.493<br>0.190 | 0.537<br>0.222 | 0.565<br>0.241 | 0.455<br>0.154 | 0.474<br>0.161 | 0.477<br>0.186 | 0.500<br>0.194 |
| **CABS-flex** | 0.461<br>0.269 | 0.470<br>0.234 | 0.517<br>0.131 | 0.571<br>0.153 | 0.550<br>0.198 | 0.566<br>0.183 | 0.509<br>0.221 | 0.530<br>0.204 |
| **NOLB** | 0.310<br>0.278 | 0.340<br>0.273 | 0.364<br>0.199 | 0.380<br>0.182 | 0.384<br>0.284 | 0.425<br>0.242 | 0.352<br>0.268 | 0.383<br>0.246 |

**Table S5.** The average Pearson's ($r_p$) and Spearman's correlation coefficients ($r_s$) between fluctuation profiles calculated for full structures with UNRES-flex, UNRES-DSSP-flex, CABS-flex, and NOLB.

## A: NMR structures

| | Secondary-structure type | | | | | | | |
|---|---|---|---|---|---|---|---|---|
| | **α** | | **β** | | **α +β** | | **all** | |
| | **$r_p$** | **$r_s$** | **$r_p$** | **$r_s$** | **$r_p$** | **$r_s$** | **$r_p$** | **$r_s$** |
| **UNRES-flex** | 0.759<br>0.171 | 0.625<br>0.184 | 0.735<br>0.152 | 0.698<br>0.116 | 0.681<br>0.212 | 0.580<br>0.148 | 0.724<br>0.188 | 0.624<br>0.164 |
| **UNRES-DSSP-flex** | 0.803<br>0.130 | 0.680<br>0.163 | 0.774<br>0.131 | 0.723<br>0.122 | 0.715<br>0.192 | 0.623<br>0.131 | 0.763<br>0.161 | 0.668<br>0.148 |
| **CABS-flex** | 0.672<br>0.217 | 0.590<br>0.245 | 0.600<br>0.183 | 0.695<br>0.129 | 0.706<br>0.213 | 0.666<br>0.164 | 0.669<br>0.212 | 0.642<br>0.200 |
| **NOLB** | 0.361<br>0.340 | 0.282<br>0.293 | 0.358<br>0.272 | 0.356<br>0.159 | 0.292<br>0.242 | 0.318<br>0.192 | 0.334<br>0.293 | 0.312<br>0.234 |

B: X-ray structures

| | Secondary-structure type | | | | | | | |
|---|---|---|---|---|---|---|---|---|
| | **α** | | **β** | | **α +β** | | **all** | |
| | **$r_p$** | **$r_s$** | **$r_p$** | **$r_s$** | **$r_p$** | **$r_s$** | **$r_p$** | **$r_s$** |
| **UNRES-flex** | 0.560<br>0.210 | 0.529<br>0.166 | 0.552<br>0.126 | 0.563<br>0.142 | 0.453<br>0.182 | 0.434<br>0.170 | 0.512<br>0.190 | 0.494<br>0.172 |
| **UNRES-DSSP-flex** | 0.594<br>0.175 | 0.503<br>0.178 | 0.524<br>0.154 | 0.525<br>0.173 | 0.492<br>0.167 | 0.449<br>0.154 | 0.535<br>0.174 | 0.484<br>0.170 |
| **CABS-flex** | 0.464<br>0.196 | 0.464<br>0.160 | 0.513<br>0.149 | 0.528<br>0.106 | 0.541<br>0.197 | 0.537<br>0.186 | 0.508<br>0.191 | 0.509<br>0.167 |
| **NOLB** | 0.562<br>0.131 | 0.537<br>0.116 | 0.518<br>0.126 | 0.478<br>0.156 | 0.589<br>0.165 | 0.590<br>0.147 | 0.565<br>0.149 | 0.548<br>0.145 |

C: All structures

| Secondary-structure type | | | | | | | |
|---|---|---|---|---|---|---|---|
| **α** | | **β** | | **α +β** | | **all** | |
| **$r_p$** | **$r_s$** | **$r_p$** | **$r_s$** | **$r_p$** | **$r_s$** | **$r_p$** | **$r_s$** |

| | | | | | | | | |
|---|---|---|---|---|---|---|---|---|
| **UNRES-flex** | 0.665<br>0.215 | 0.580<br>0.182 | 0.648<br>0.167 | 0.634<br>0.146 | 0.559<br>0.227 | 0.502<br>0.176 | 0.618<br>0.217 | 0.559<br>0.180 |
| **UNRES-DSSP-flex** | 0.704<br>0.185 | 0.596<br>0.192 | 0.655<br>0.190 | 0.628<br>0.178 | 0.595<br>0.211 | 0.530<br>0.168 | 0.649<br>0.203 | 0.576<br>0.184 |
| **3CABS-flex** | 0.574<br>0.232 | 0.530<br>0.218 | 0.559<br>0.173 | 0.615<br>0.145 | 0.617<br>0.220 | 0.597<br>0.188 | 0.588<br>0.217 | 0.575<br>0.196 |
| **NOLB** | 0.456<br>0.281 | 0.403<br>0.260 | 0.434<br>0.230 | 0.415<br>0.169 | 0.451<br>0.252 | 0.464<br>0.217 | 0.449<br>0.259 | 0.430<br>0.228 |

**Table S6.** ANOVA results, shown as significance levels (expressed as p-values), of the Pearson's ($r_p$) and Spearman's ($r_s$) correlation coefficients between the fluctuation profiles calculated with UNRES-flex, UNRES-DSSP-flex, CABS-flex, and NOLB and the corresponding experimental profiles. The analysis has been carried out for truncated structures. The values in the 'Method' and 'Secondary structure' columns indicate the significance of the dependence of a correlation coefficient on fluctuation-profile-prediction method and on secondary-structure type (α, β, or α+β, respectively). The values in the 'Interaction column' indicate the significance of the simultaneous dependence of a correlation coefficient on the prediction method and secondary-structure type. The p-values lower than 0.05 (indicating statistical significance) are in red font and higher values (corresponding to weak or no statistical significance) are in green font.

| **Significance level** | $r_p$/ $r_s$ | **Method** | **Secondary structure** | **Interaction** |
|---|---|---|---|---|
| | | | **NMR structures** | |
| | $r_p$ | <0.001 | 0.082 | 0.82 |
| | $r_s$ | <0.001 | 0.016 | 0.94 |
| | | | **X-ray structures** | |
| | $r_p$ | 0.040 | 0.42 | 0.14 |
| | $r_s$ | 0.17 | 0.60 | 0.069 |
| | | | **All structures** | |
| | $r_p$ | <0.001 | 0.074 | 0.37 |
| | $r_s$ | <0.001 | 0.024 | 0.19 |

**Table S7.** Significance of differences, expressed as p-values, of the Pearson's ($r_p$) and Spearman's ($r_s$) correlation coefficients of fluctuation profiles, corresponding to truncated structures, calculated with UNRES-flex, UNRES-DSSP-flex, CABS-flex, and NOLB assessed by the two sample t-test depending on structure-determination method and secondary-structure class. The p-values with the "-" sign indicate that the respective correlation coefficients are greater for the method in the left column (Method 1), while the "+" sign means that the correlation coefficients are greater for the method in the right column (Method

2). The p-values lower than 0.05 (indicating statistical significance) are in red font and higher values (corresponding to weak or no statistical significance) are in green font.

A: NMR structures, Pearson coefficient ($r_p$)

| **Significance level** | **Method 1** | **Method 2** | **Secondary-structure type** | | | |
|---|---|---|---|---|---|---|
| | | | **α** | **β** | **α + β** | **all** |
| | **UNRES-flex** | **UNRES-DSSP-flex** | + 0.31 | + 0.64 | + 0.22 | + 0.42 |
| | | **CABS-flex** | + 0.15 | + 0.88 | + 0.0024 | + 0.35 |
| | | **NOLB** | - <0.001 | - 0.0060 | - <0.001 | - <0.001 |
| | **UNRES-DSSP-flex** | **CABS-flex** | + 0.47 | - 0.67 | + 0.046 | + 0.36 |
| | | **NOLB** | - <0.001 | - 0.0022 | - <0.001 | - <0.001 |
| | **CABS-flex** | **NOLB** | - <0.001 | - <0.001 | - <0.001 | - <0.001 |

B: NMR structures, Spearman coefficient ($r_s$)

| **Significance level** | **Method 1** | **Method 2** | **Secondary-structure type** | | | |
|---|---|---|---|---|---|---|
| | | | **α** | **B** | **α + β** | **all** |
| | **UNRES-flex** | **UNRES-DSSP-flex** | + 0.31 | + 0.64 | + 0.22 | + 0.42 |
| | | **CABS-flex** | + 0.15 | + 0.88 | + 0.0024 | + 0.35 |
| | | **NOLB** | - <0.001 | - 0.0060 | - <0.001 | - <0.001 |
| | **UNRES-DSSP-flex** | **CABS-flex** | + 0.47 | - 0.67 | + 0.046 | + 0.36 |
| | | **NOLB** | - <0.001 | - 0.0022 | - <0.001 | - <0.001 |
| | **CABS-flex** | **NOLB** | - <0.001 | - <0.001 | - <0.001 | - <0.001 |

C: X-ray structures, Pearson coefficient ($r_p$)

| **can ce** | **Method 1** | **Method 2** | **Secondary-structure type** | | | |
|---|---|---|---|---|---|---|
| | | | **α** | **B** | **α + β** | **all** |

| | Method 1 | Method 2 | α | β | α + β | all |
|---|---|---|---|---|---|---|
| | UNRES-flex | UNRES-DSSP-flex | + 0.91 | - 0.70 | + 0.52 | + 0.65 |
| | | CABS-flex | - 0.30 | - 0.66 | + 0.061 | + 0.24 |
| | | NOLB | + 0.41 | - 0.70 | + <0.001 | + 0.0073 |
| | UNRES-DSSP-flex | CABS-flex | - 0.25 | - 0.99 | + 0.17 | + 0.35 |
| | | NOLB | + 0.50 | - 0.94 | + 0.0035 | + 0.31 |
| | CABS-flex | NOLB | + 0.052 | + 0.94 | + 0.18 | + 0.30 |

D: X-ray structures, Spearman coefficient ($r_s$)

| Significance level | Method 1 | Method 2 | Secondary-structure type | | | |
|---|---|---|---|---|---|---|
| | | | α | B | α + β | all |
| | UNRES-flex | UNRES-DSSP-flex | - 0.60 | - 0.61 | + 0.78 | - 0.70 |
| | | CABS-flex | - 0.28 | - 0.58 | + 0.061 | + 0.22 |
| | | NOLB | + 0.84 | - 0.24 | + 0.0021 | + 0.25 |
| | UNRES-DSSP-flex | CABS-flex | - 0.66 | + 0.92 | + 0.092 | + 0.39 |
| | | NOLB | + 0.45 | - 0.58 | + 0.0033 | + 0.26 |
| | CABS-flex | NOLB | + 0.16 | - 0.41 | + 0.30 | + 0.29 |

E: All structures, Pearson coefficient ($r_p$)

| Significance level | Method 1 | Method 2 | Secondary-structure type | | | |
|---|---|---|---|---|---|---|
| | | | α | β | α + β | all |
| | UNRES-flex | UNRES-DSSP-flex | + 0.44 | + 0.86 | + 0.19 | + 0.41 |
| | | CABS-flex | +0.63 | - 0.90 | + <0.001 | + 0.38 |
| | | NOLB | - 0.023 | - 0.015 | - 0.61 | - 0.31 |
| | UNRES-DSSP-flex | CABS-flex | - 0.89 | - 0.74 | + 0.019 | + 0.43 |
| | | NOLB | - 0.0053 | - 0.013 | - 0.17 | - 0.10 |
| | CABS-flex | NOLB | - 0.020 | - 0.0063 | - 0.0034 | - 0.0090 |

F: All structures, Spearman coefficient ($r_s$)

| can ce | Method 1 | Method 2 | Secondary-structure type | | | |
|---|---|---|---|---|---|---|
| | | | α | β | α + β | all |

| | | | | | | |
|---|---|---|---|---|---|---|
| | **UNRES-flex** | **UNRES-DSSP-flex** | + 0.66 | - 0.94 | + 0.40 | + 0.59 |
| | | **CABS-flex** | - 0.94 | - 1.0 | + 0.0027 | + 0.49 |
| | | **NOLB** | - 0.014 | - 0.0045 | - 0.69 | - 0.34 |
| | **UNRES-DSSP-flex** | **CABS-flex** | - 0.65 | + 0.93 | + 0.021 | + 0.40 |
| | | **NOLB** | - 0.0067 | - 0.0091 | - 0.28 | - 0.14 |
| | **CABS-flex** | **NOLB** | - 0.031 | - <0.001 | - 0.0043 | - 0.012 |

**Table S8.** PDB codes of NMR structures with low similarity of the fluctuation profiles estimated from NMR ensembles and those calculated with a given method.

| **method** | **structure with $r_p \leq 0.2$** |
|---|---|
| **UNRES-flex** | 1ACP, 1BAL, 1GAB, 1HRF, 1J7O, 1LEB, 1POU, 2N2U, 3CI2 |
| **UNRES-DSSP-flex** | 1BAL, 1GAB, 1HRF, 1J7O, 2HI3, 2L09 |
| **CABS-flex** | 1BAL, 1ED7, 1J7O, 1K8B, 2RGF |
| **NOLB** | 1A6S, 1ACP, 1AH9, 1BAL, 1CLB, 1E0G, 1ED7, 1FEX, 1GHH, 1HNS, 1HYW, 1IQO, 1IYR, 1J7O, 1K8B, 1L2Y, 1LEB, 1POU, 1RIJ, 1STU, 1TPN, 2E7N, 2HI3, 2KYW, 2L09, 2LVC, 2M6Q, 2MQ8, 2RGF, 3CI2, 3NCM |

**Figure S1.** Normalized fluctuation profiles (RMSFN, dimensionless) calculated from NMR ensembles or X-ray B-factors (red line) and the corresponding profiled predicted by UNRES-flex (ligh-blue line), UNRES-DSSP-flex (green line), CABS-flex (yellow line), and NOLB (blue line). Except for NOLB, the fluctuation profiles are averaged over three simulations. The Pearson ($r_p$) and Spearman ($r_s$) coefficients for the correlation between the experimental and predicted profiles are shown in each panel. The solid orange and wave green lines below the abscissa of each panel indicate the β-sheet and α-helical structure, respectively. The analyses were carried out for truncated structures.

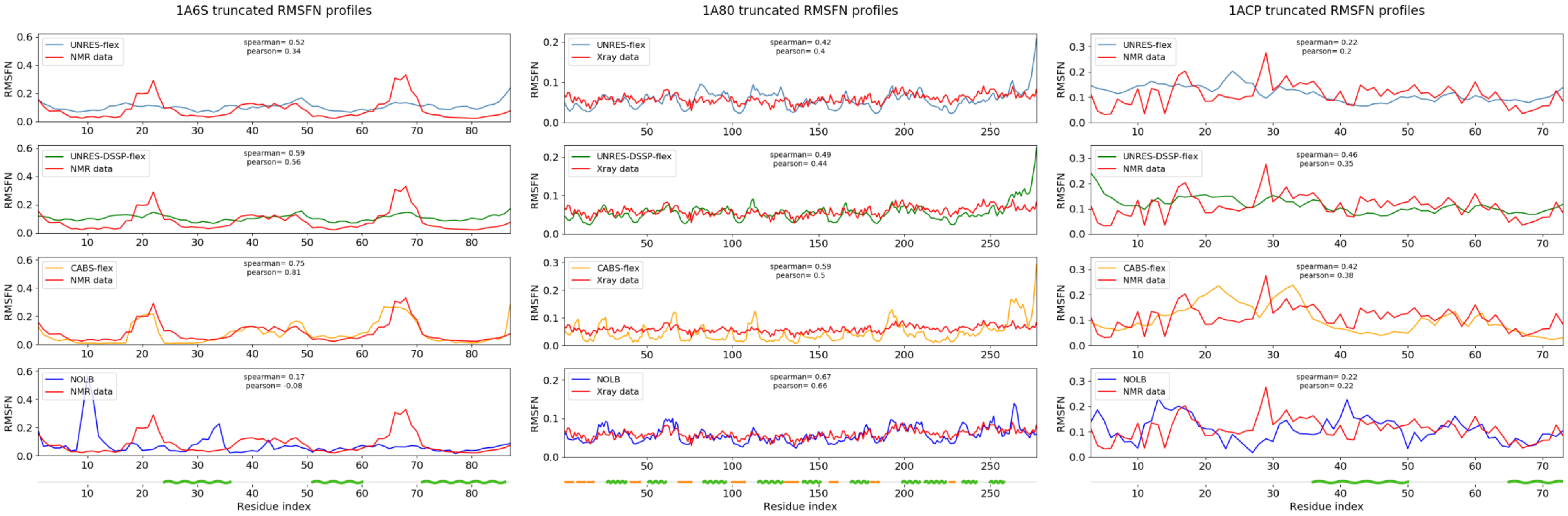
1A6S truncated RMSFN profiles
UNRES-flex
NMR data
spearman= 0.52
pearson= 0.34
UNRES-DSSP-flex
NMR data
spearman= 0.59
pearson= 0.56
CABS-flex
NMR data
spearman= 0.75
pearson= 0.81
NOLB
NMR data
spearman= 0.17
pearson= -0.08
RMSFN
Residue index
1A80 truncated RMSFN profiles
UNRES-flex
Xray data
spearman= 0.42
pearson= 0.4
UNRES-DSSP-flex
Xray data
spearman= 0.49
pearson= 0.44
CABS-flex
Xray data
spearman= 0.59
pearson= 0.5
NOLB
Xray data
spearman= 0.67
pearson= 0.66
RMSFN
Residue index
1ACP truncated RMSFN profiles
UNRES-flex
NMR data
spearman= 0.22
pearson= 0.2
UNRES-DSSP-flex
NMR data
spearman= 0.46
pearson= 0.35
CABS-flex
NMR data
spearman= 0.42
pearson= 0.38
NOLB
NMR data
spearman= 0.22
pearson= 0.22
RMSFN
Residue index

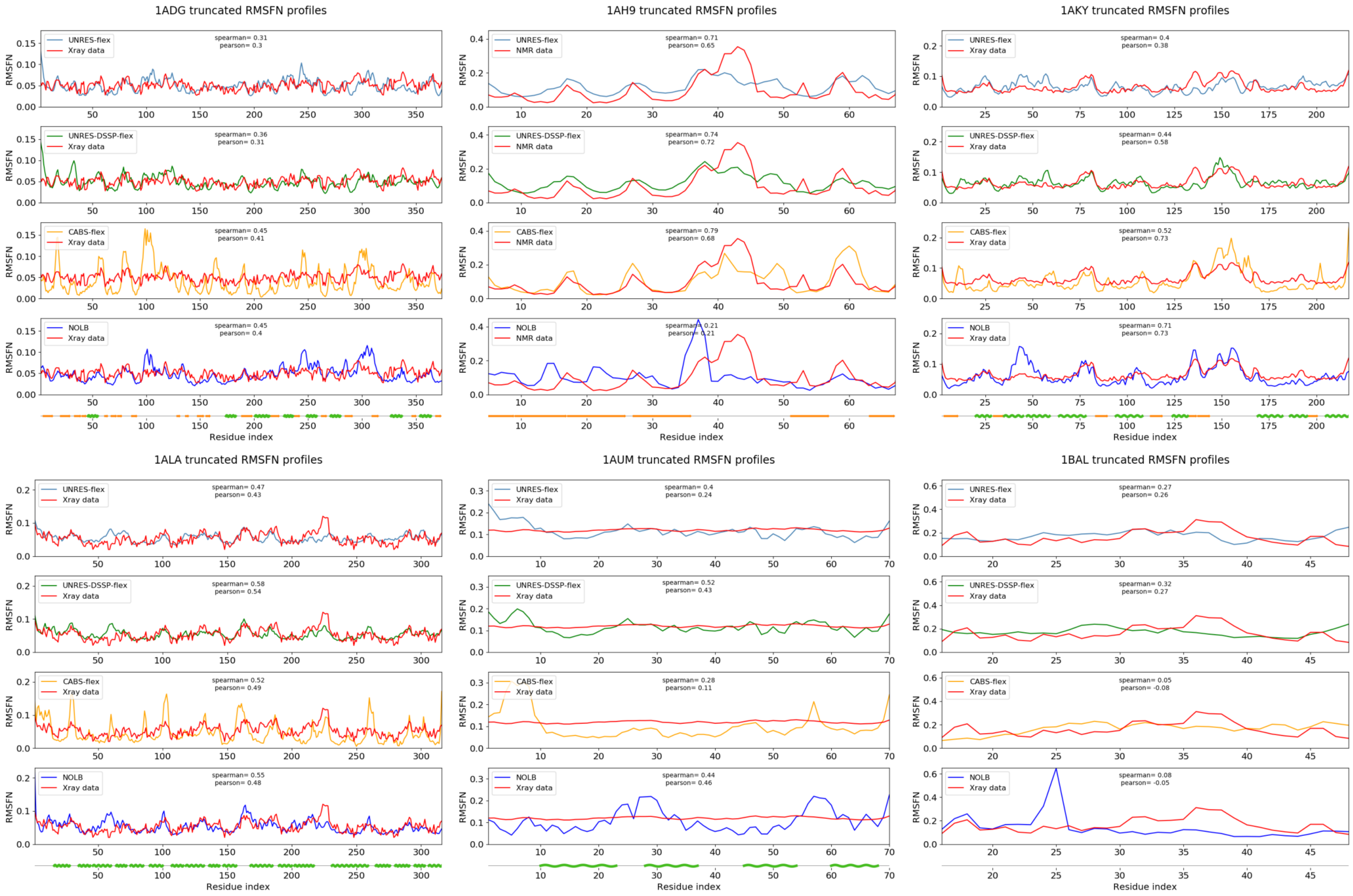

1ADG truncated RMSFN profiles
UNRES-flex
Xray data
spearman= 0.31
pearson= 0.3
UNRES-DSSP-flex
Xray data
spearman= 0.36
pearson= 0.31
CABS-flex
Xray data
spearman= 0.45
pearson= 0.41
NOLB
Xray data
spearman= 0.45
pearson= 0.4
RMSFN
Residue index
1AH9 truncated RMSFN profiles
UNRES-flex
NMR data
spearman= 0.71
pearson= 0.65
UNRES-DSSP-flex
NMR data
spearman= 0.74
pearson= 0.72
CABS-flex
NMR data
spearman= 0.79
pearson= 0.68
NOLB
NMR data
spearman= 0.21
pearson= 0.21
1AKY truncated RMSFN profiles
UNRES-flex
Xray data
spearman= 0.4
pearson= 0.38
UNRES-DSSP-flex
Xray data
spearman= 0.44
pearson= 0.58
CABS-flex
Xray data
spearman= 0.52
pearson= 0.73
NOLB
Xray data
spearman= 0.71
pearson= 0.73
1ALA truncated RMSFN profiles
UNRES-flex
Xray data
spearman= 0.47
pearson= 0.43
UNRES-DSSP-flex
Xray data
spearman= 0.58
pearson= 0.54
CABS-flex
Xray data
spearman= 0.52
pearson= 0.49
NOLB
Xray data
spearman= 0.55
pearson= 0.48
1AUM truncated RMSFN profiles
UNRES-flex
Xray data
spearman= 0.4
pearson= 0.24
UNRES-DSSP-flex
Xray data
spearman= 0.52
pearson= 0.43
CABS-flex
Xray data
spearman= 0.28
pearson= 0.11
NOLB
Xray data
spearman= 0.44
pearson= 0.46
1BAL truncated RMSFN profiles
UNRES-flex
Xray data
spearman= 0.27
pearson= 0.26
UNRES-DSSP-flex
Xray data
spearman= 0.32
pearson= 0.27
CABS-flex
Xray data
spearman= 0.05
pearson= -0.08
NOLB
Xray data
spearman= 0.08
pearson= -0.05

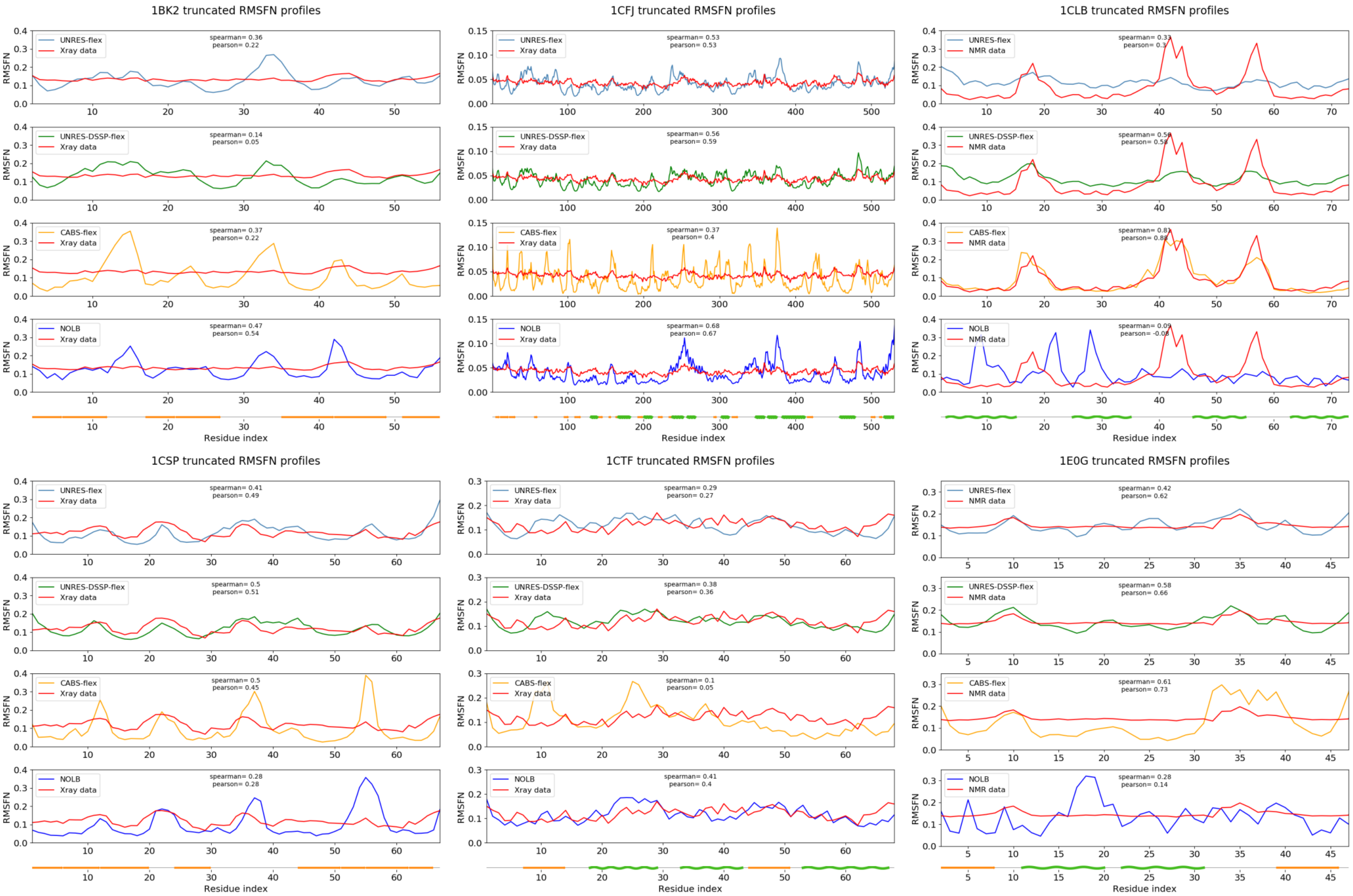
1BK2 truncated RMSFN profiles
UNRES-flex
Xray data
spearman= 0.36
pearson= 0.22
UNRES-DSSP-flex
spearman= 0.14
pearson= 0.05
CABS-flex
spearman= 0.37
pearson= 0.22
NOLB
spearman= 0.47
pearson= 0.54
1CFJ truncated RMSFN profiles
spearman= 0.53
pearson= 0.53
spearman= 0.56
pearson= 0.59
spearman= 0.37
pearson= 0.4
spearman= 0.68
pearson= 0.67
1CLB truncated RMSFN profiles
NMR data
1CSP truncated RMSFN profiles
spearman= 0.41
pearson= 0.49
spearman= 0.5
pearson= 0.51
spearman= 0.5
pearson= 0.45
spearman= 0.28
pearson= 0.28
1CTF truncated RMSFN profiles
spearman= 0.29
pearson= 0.27
spearman= 0.38
pearson= 0.36
spearman= 0.1
pearson= 0.05
spearman= 0.41
pearson= 0.4
1E0G truncated RMSFN profiles
spearman= 0.42
pearson= 0.62
spearman= 0.58
pearson= 0.66
spearman= 0.61
pearson= 0.73
spearman= 0.28
pearson= 0.14
RMSFN
Residue index

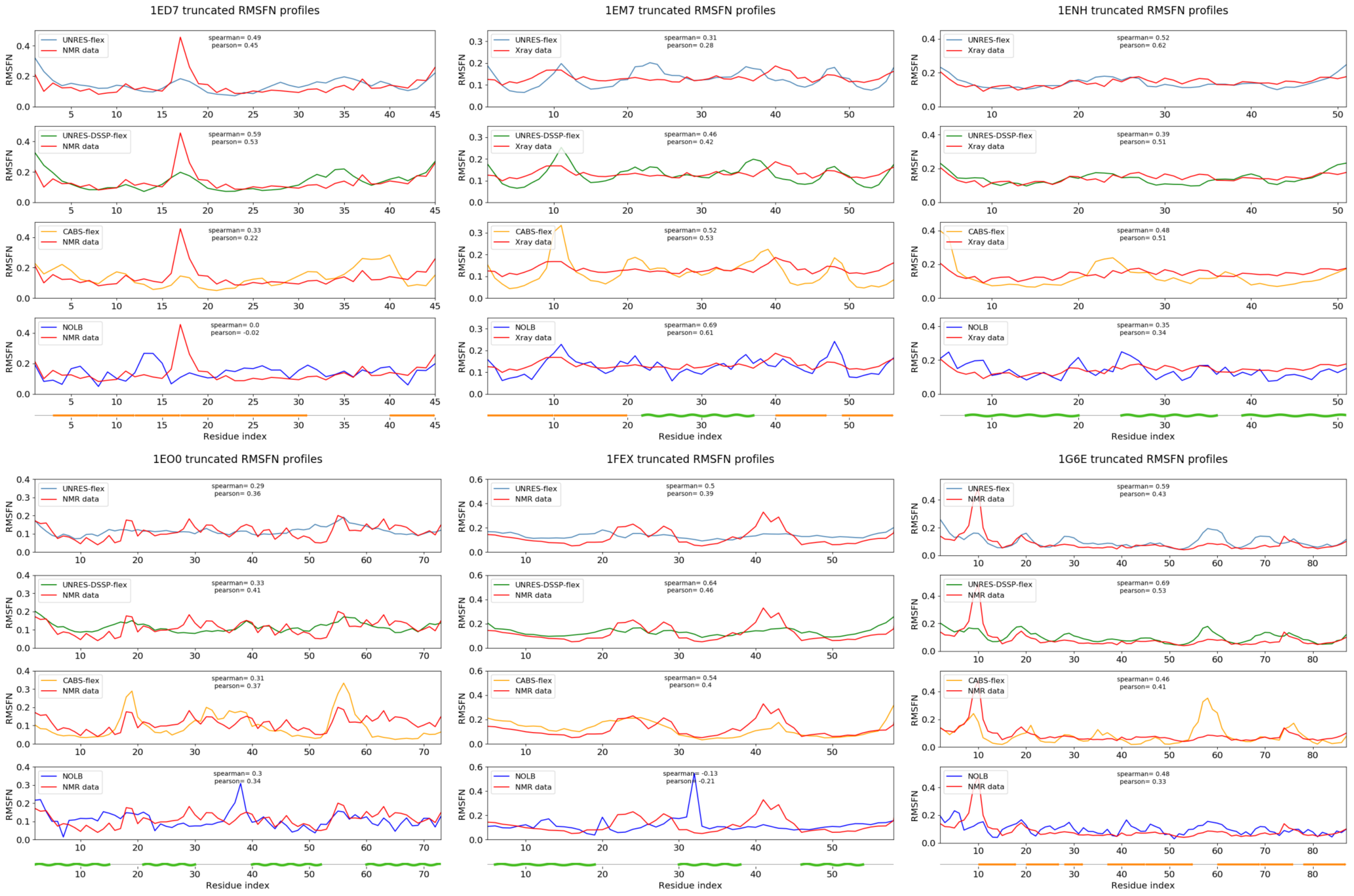
1ED7 truncated RMSFN profiles
UNRES-flex
NMR data
spearman= 0.49
pearson= 0.45
UNRES-DSSP-flex
NMR data
spearman= 0.59
pearson= 0.53
CABS-flex
NMR data
spearman= 0.33
pearson= 0.22
NOLB
NMR data
spearman= 0.0
pearson= -0.02
RMSFN
Residue index
1EM7 truncated RMSFN profiles
UNRES-flex
Xray data
spearman= 0.31
pearson= 0.28
UNRES-DSSP-flex
Xray data
spearman= 0.46
pearson= 0.42
CABS-flex
Xray data
spearman= 0.52
pearson= 0.53
NOLB
Xray data
spearman= 0.69
pearson= 0.61
RMSFN
Residue index
1ENH truncated RMSFN profiles
UNRES-flex
Xray data
spearman= 0.52
pearson= 0.62
UNRES-DSSP-flex
Xray data
spearman= 0.39
pearson= 0.51
CABS-flex
Xray data
spearman= 0.48
pearson= 0.51
NOLB
Xray data
spearman= 0.35
pearson= 0.34
RMSFN
Residue index
1EO0 truncated RMSFN profiles
UNRES-flex
NMR data
spearman= 0.29
pearson= 0.36
UNRES-DSSP-flex
NMR data
spearman= 0.33
pearson= 0.41
CABS-flex
NMR data
spearman= 0.31
pearson= 0.37
NOLB
NMR data
spearman= 0.3
pearson= 0.34
RMSFN
Residue index
1FEX truncated RMSFN profiles
UNRES-flex
NMR data
spearman= 0.5
pearson= 0.39
UNRES-DSSP-flex
NMR data
spearman= 0.64
pearson= 0.46
CABS-flex
NMR data
spearman= 0.54
pearson= 0.4
NOLB
NMR data
spearman= -0.13
pearson= -0.21
RMSFN
Residue index
1G6E truncated RMSFN profiles
UNRES-flex
NMR data
spearman= 0.59
pearson= 0.43
UNRES-DSSP-flex
NMR data
spearman= 0.69
pearson= 0.53
CABS-flex
NMR data
spearman= 0.46
pearson= 0.41
NOLB
NMR data
spearman= 0.48
pearson= 0.33
RMSFN
Residue index

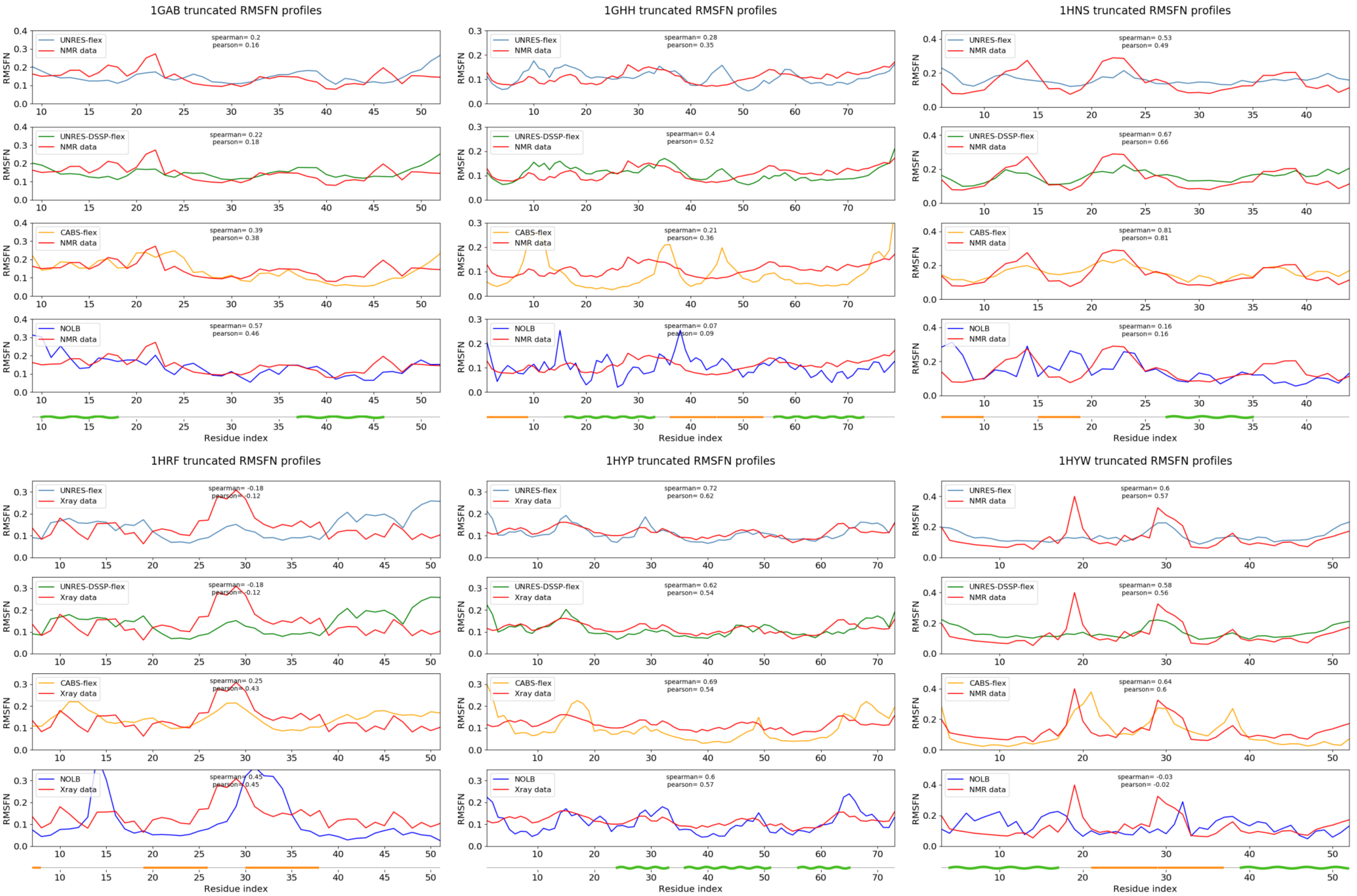
1GAB truncated RMSFN profiles
UNRES-flex
NMR data
spearman= 0.2
pearson= 0.16
UNRES-DSSP-flex
NMR data
spearman= 0.22
pearson= 0.18
CABS-flex
NMR data
spearman= 0.39
pearson= 0.38
NOLB
NMR data
spearman= 0.57
pearson= 0.46
RMSFN
Residue index
1GHH truncated RMSFN profiles
UNRES-flex
NMR data
spearman= 0.28
pearson= 0.35
UNRES-DSSP-flex
NMR data
spearman= 0.4
pearson= 0.52
CABS-flex
NMR data
spearman= 0.21
pearson= 0.36
NOLB
NMR data
spearman= 0.07
pearson= 0.09
1HNS truncated RMSFN profiles
UNRES-flex
NMR data
spearman= 0.53
pearson= 0.49
UNRES-DSSP-flex
NMR data
spearman= 0.67
pearson= 0.66
CABS-flex
NMR data
spearman= 0.81
pearson= 0.81
NOLB
NMR data
spearman= 0.16
pearson= 0.16
1HRF truncated RMSFN profiles
UNRES-flex
Xray data
spearman= -0.18
pearson= -0.12
UNRES-DSSP-flex
Xray data
spearman= -0.18
pearson= -0.12
CABS-flex
Xray data
spearman= 0.25
pearson= 0.43
NOLB
Xray data
spearman= 0.45
pearson= 0.45
1HYP truncated RMSFN profiles
UNRES-flex
Xray data
spearman= 0.72
pearson= 0.62
UNRES-DSSP-flex
Xray data
spearman= 0.62
pearson= 0.54
CABS-flex
Xray data
spearman= 0.69
pearson= 0.54
NOLB
Xray data
spearman= 0.6
pearson= 0.57
1HYW truncated RMSFN profiles
UNRES-flex
NMR data
spearman= 0.6
pearson= 0.57
UNRES-DSSP-flex
NMR data
spearman= 0.58
pearson= 0.56
CABS-flex
NMR data
spearman= 0.64
pearson= 0.6
NOLB
NMR data
spearman= -0.03
pearson= -0.02

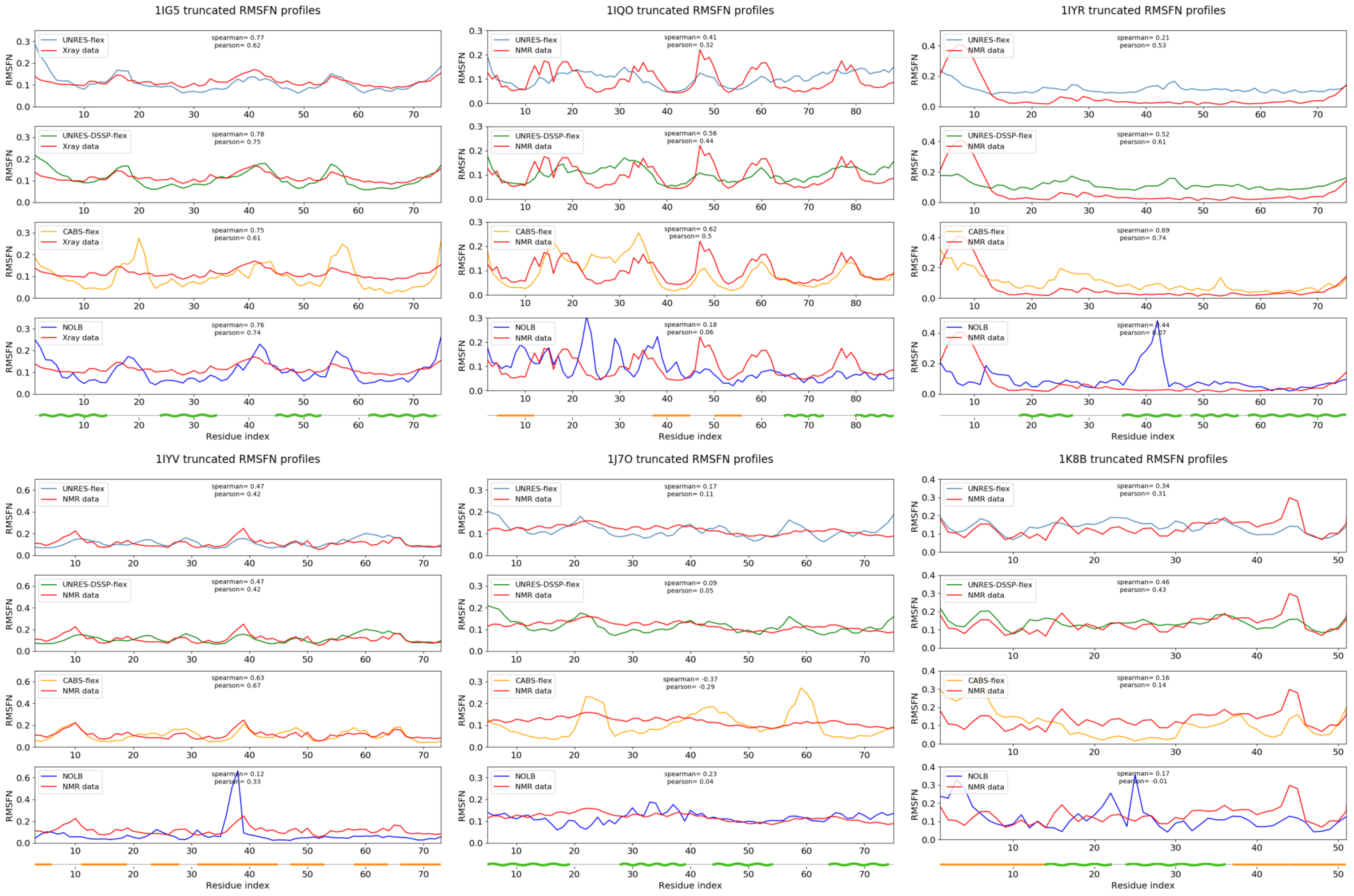
1IG5 truncated RMSFN profiles
UNRES-flex
Xray data
spearman= 0.77
pearson= 0.62
UNRES-DSSP-flex
Xray data
spearman= 0.78
pearson= 0.75
CABS-flex
Xray data
spearman= 0.75
pearson= 0.61
NOLB
Xray data
spearman= 0.76
pearson= 0.74
1IQO truncated RMSFN profiles
UNRES-flex
NMR data
spearman= 0.41
pearson= 0.32
UNRES-DSSP-flex
NMR data
spearman= 0.56
pearson= 0.44
CABS-flex
NMR data
spearman= 0.62
pearson= 0.5
NOLB
NMR data
spearman= 0.18
pearson= 0.06
1IYR truncated RMSFN profiles
UNRES-flex
NMR data
spearman= 0.21
pearson= 0.53
UNRES-DSSP-flex
NMR data
spearman= 0.52
pearson= 0.61
CABS-flex
NMR data
spearman= 0.69
pearson= 0.74
NOLB
NMR data
spearman= 0.44
pearson= 0.07
1IYV truncated RMSFN profiles
UNRES-flex
NMR data
spearman= 0.47
pearson= 0.42
UNRES-DSSP-flex
NMR data
spearman= 0.47
pearson= 0.42
CABS-flex
NMR data
spearman= 0.63
pearson= 0.67
NOLB
NMR data
spearman= 0.12
pearson= 0.33
1J7O truncated RMSFN profiles
UNRES-flex
NMR data
spearman= 0.17
pearson= 0.11
UNRES-DSSP-flex
NMR data
spearman= 0.09
pearson= 0.05
CABS-flex
NMR data
spearman= -0.37
pearson= -0.29
NOLB
NMR data
spearman= 0.23
pearson= 0.04
1K8B truncated RMSFN profiles
UNRES-flex
NMR data
spearman= 0.34
pearson= 0.31
UNRES-DSSP-flex
NMR data
spearman= 0.46
pearson= 0.43
CABS-flex
NMR data
spearman= 0.16
pearson= 0.14
NOLB
NMR data
spearman= 0.17
pearson= -0.01
RMSFN
Residue index

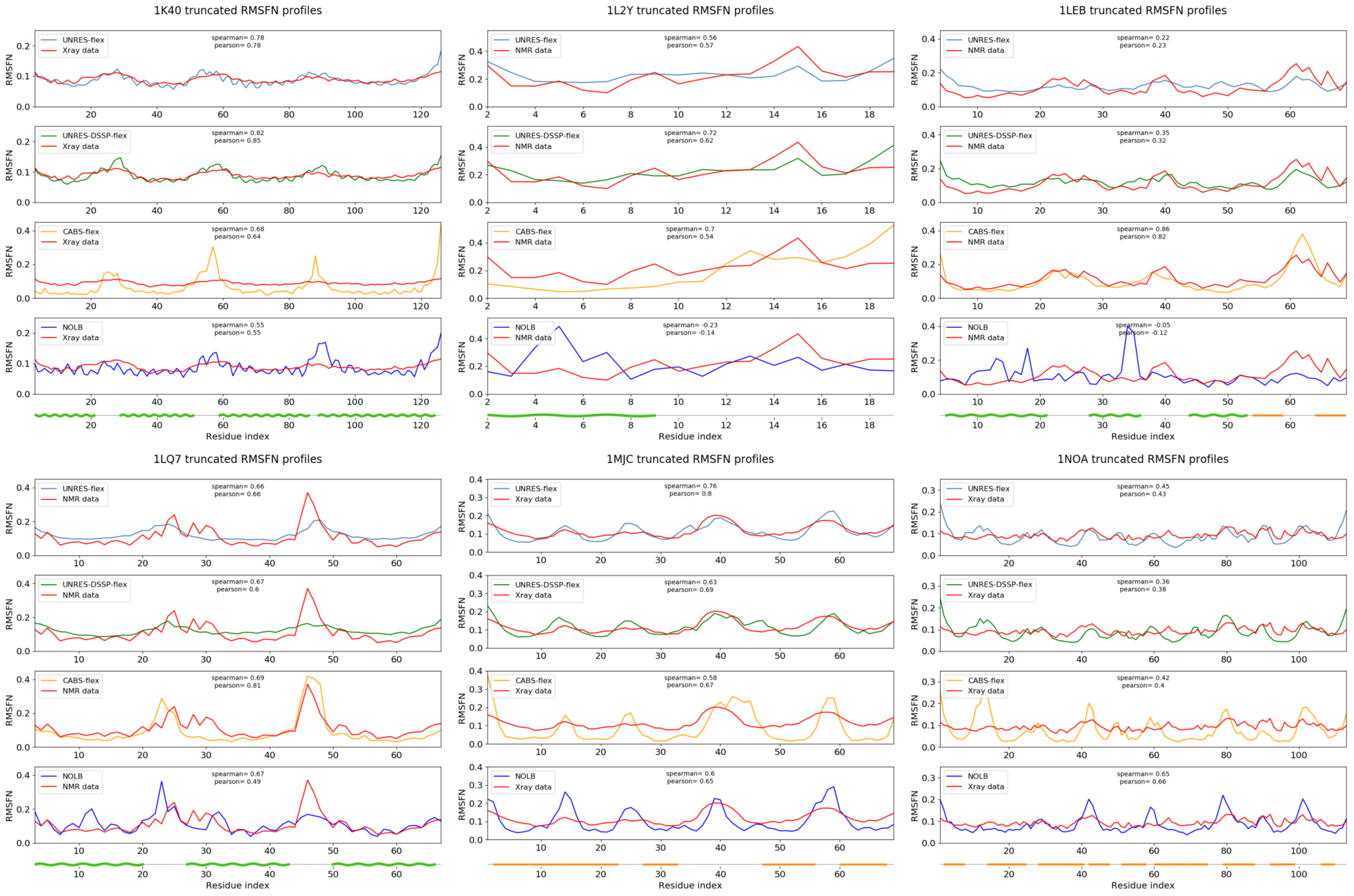
1K40 truncated RMSFN profiles
UNRES-flex
Xray data
spearman= 0.78
pearson= 0.78
UNRES-DSSP-flex
Xray data
spearman= 0.82
pearson= 0.85
CABS-flex
Xray data
spearman= 0.68
pearson= 0.64
NOLB
Xray data
spearman= 0.55
pearson= 0.55
1L2Y truncated RMSFN profiles
UNRES-flex
NMR data
spearman= 0.56
pearson= 0.57
UNRES-DSSP-flex
NMR data
spearman= 0.72
pearson= 0.62
CABS-flex
NMR data
spearman= 0.7
pearson= 0.54
NOLB
NMR data
spearman= -0.23
pearson= -0.14
1LEB truncated RMSFN profiles
UNRES-flex
NMR data
spearman= 0.22
pearson= 0.23
UNRES-DSSP-flex
NMR data
spearman= 0.35
pearson= 0.32
CABS-flex
NMR data
spearman= 0.86
pearson= 0.82
NOLB
NMR data
spearman= -0.05
pearson= -0.12
1LQ7 truncated RMSFN profiles
UNRES-flex
NMR data
spearman= 0.66
pearson= 0.66
UNRES-DSSP-flex
NMR data
spearman= 0.67
pearson= 0.6
CABS-flex
NMR data
spearman= 0.69
pearson= 0.81
NOLB
NMR data
spearman= 0.67
pearson= 0.49
1MJC truncated RMSFN profiles
UNRES-flex
Xray data
spearman= 0.76
pearson= 0.8
UNRES-DSSP-flex
Xray data
spearman= 0.63
pearson= 0.69
CABS-flex
Xray data
spearman= 0.58
pearson= 0.67
NOLB
Xray data
spearman= 0.6
pearson= 0.65
1NOA truncated RMSFN profiles
UNRES-flex
Xray data
spearman= 0.45
pearson= 0.43
UNRES-DSSP-flex
Xray data
spearman= 0.36
pearson= 0.38
CABS-flex
Xray data
spearman= 0.42
pearson= 0.4
NOLB
Xray data
spearman= 0.65
pearson= 0.66
RMSFN
Residue index

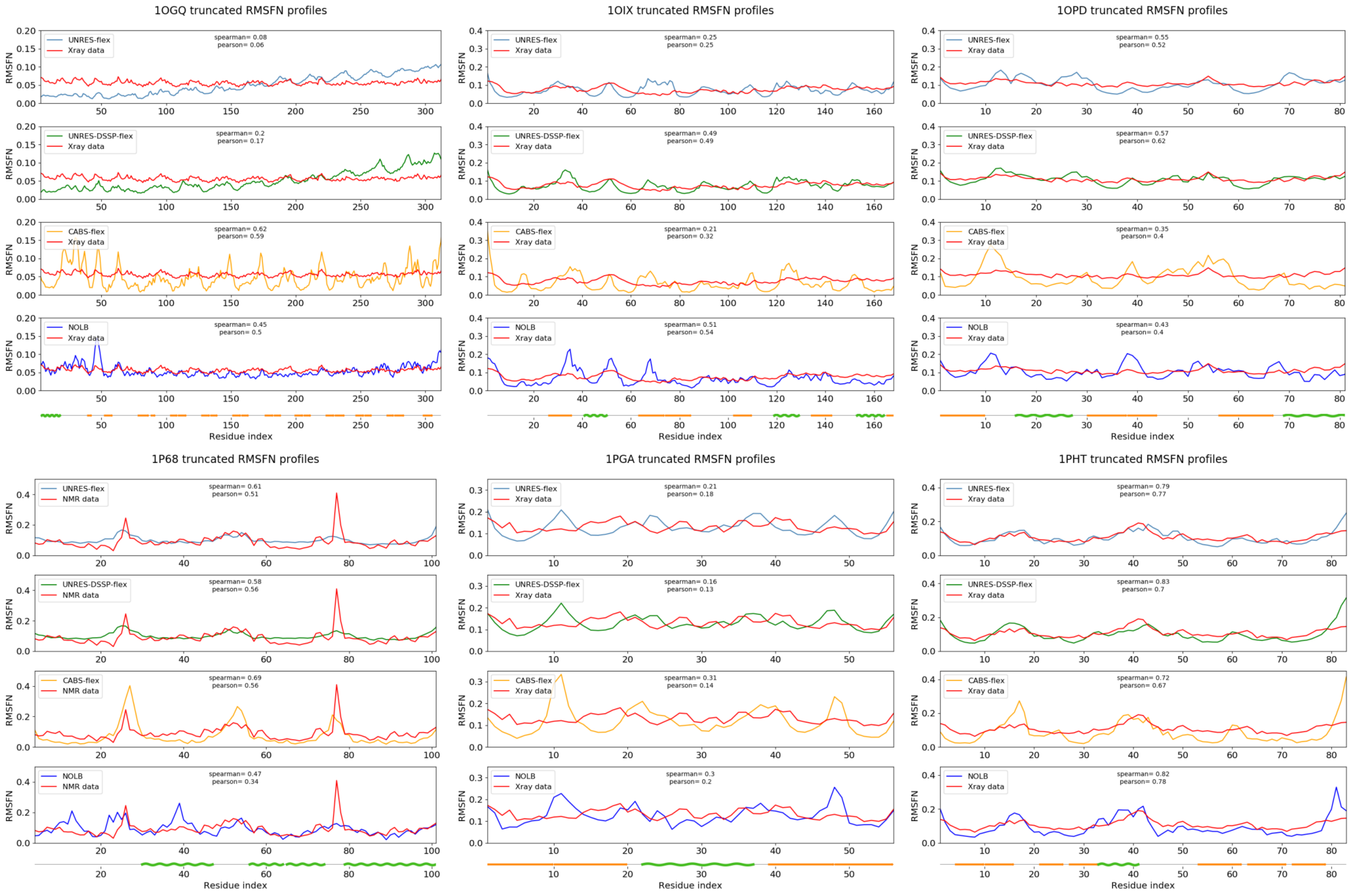

1OGQ truncated RMSFN profiles
UNRES-flex
Xray data
spearman= 0.08
pearson= 0.06
UNRES-DSSP-flex
Xray data
spearman= 0.2
pearson= 0.17
CABS-flex
Xray data
spearman= 0.62
pearson= 0.59
NOLB
Xray data
spearman= 0.45
pearson= 0.5
RMSFN
Residue index
1OIX truncated RMSFN profiles
UNRES-flex
Xray data
spearman= 0.25
pearson= 0.25
UNRES-DSSP-flex
Xray data
spearman= 0.49
pearson= 0.49
CABS-flex
Xray data
spearman= 0.21
pearson= 0.32
NOLB
Xray data
spearman= 0.51
pearson= 0.54
RMSFN
Residue index
1OPD truncated RMSFN profiles
UNRES-flex
Xray data
spearman= 0.55
pearson= 0.52
UNRES-DSSP-flex
Xray data
spearman= 0.57
pearson= 0.62
CABS-flex
Xray data
spearman= 0.35
pearson= 0.4
NOLB
Xray data
spearman= 0.43
pearson= 0.4
RMSFN
Residue index
1P68 truncated RMSFN profiles
UNRES-flex
NMR data
spearman= 0.61
pearson= 0.51
UNRES-DSSP-flex
NMR data
spearman= 0.58
pearson= 0.56
CABS-flex
NMR data
spearman= 0.69
pearson= 0.56
NOLB
NMR data
spearman= 0.47
pearson= 0.34
RMSFN
Residue index
1PGA truncated RMSFN profiles
UNRES-flex
Xray data
spearman= 0.21
pearson= 0.18
UNRES-DSSP-flex
Xray data
spearman= 0.16
pearson= 0.13
CABS-flex
Xray data
spearman= 0.31
pearson= 0.14
NOLB
Xray data
spearman= 0.3
pearson= 0.2
RMSFN
Residue index
1PHT truncated RMSFN profiles
UNRES-flex
Xray data
spearman= 0.79
pearson= 0.77
UNRES-DSSP-flex
Xray data
spearman= 0.83
pearson= 0.7
CABS-flex
Xray data
spearman= 0.72
pearson= 0.67
NOLB
Xray data
spearman= 0.82
pearson= 0.78
RMSFN
Residue index

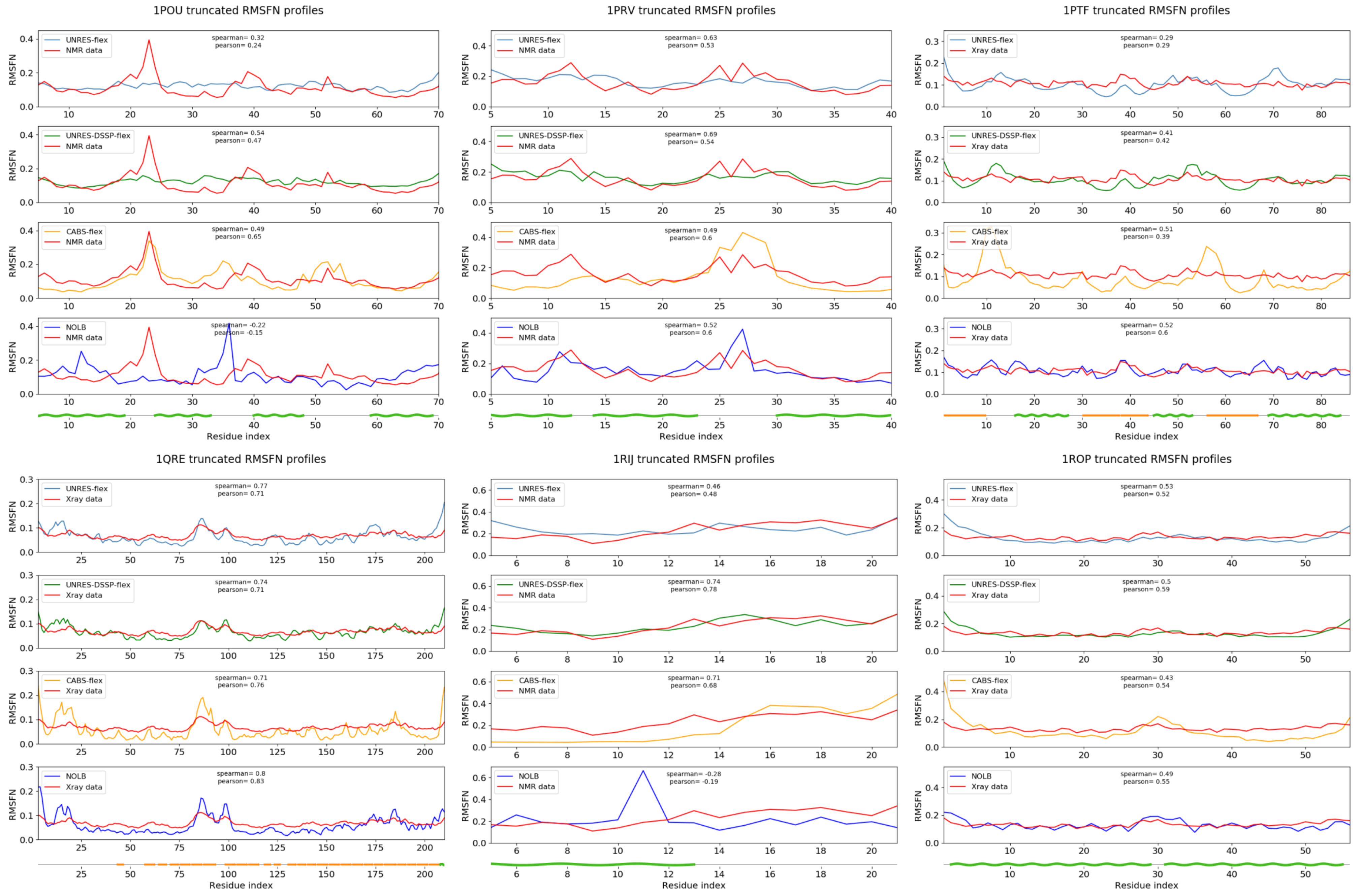
1POU truncated RMSFN profiles
UNRES-flex
NMR data
spearman= 0.32
pearson= 0.24
UNRES-DSSP-flex
NMR data
spearman= 0.54
pearson= 0.47
CABS-flex
NMR data
spearman= 0.49
pearson= 0.65
NOLB
NMR data
spearman= -0.22
pearson= -0.15
1PRV truncated RMSFN profiles
UNRES-flex
NMR data
spearman= 0.63
pearson= 0.53
UNRES-DSSP-flex
NMR data
spearman= 0.69
pearson= 0.54
CABS-flex
NMR data
spearman= 0.49
pearson= 0.6
NOLB
NMR data
spearman= 0.52
pearson= 0.6
1PTF truncated RMSFN profiles
UNRES-flex
Xray data
spearman= 0.29
pearson= 0.29
UNRES-DSSP-flex
Xray data
spearman= 0.41
pearson= 0.42
CABS-flex
Xray data
spearman= 0.51
pearson= 0.39
NOLB
Xray data
spearman= 0.52
pearson= 0.6
1QRE truncated RMSFN profiles
UNRES-flex
Xray data
spearman= 0.77
pearson= 0.71
UNRES-DSSP-flex
Xray data
spearman= 0.74
pearson= 0.71
CABS-flex
Xray data
spearman= 0.71
pearson= 0.76
NOLB
Xray data
spearman= 0.8
pearson= 0.83
1RIJ truncated RMSFN profiles
UNRES-flex
NMR data
spearman= 0.46
pearson= 0.48
UNRES-DSSP-flex
NMR data
spearman= 0.74
pearson= 0.78
CABS-flex
NMR data
spearman= 0.71
pearson= 0.68
NOLB
NMR data
spearman= -0.28
pearson= -0.19
1ROP truncated RMSFN profiles
UNRES-flex
Xray data
spearman= 0.53
pearson= 0.52
UNRES-DSSP-flex
Xray data
spearman= 0.5
pearson= 0.59
CABS-flex
Xray data
spearman= 0.43
pearson= 0.54
NOLB
Xray data
spearman= 0.49
pearson= 0.55
RMSFN
Residue index

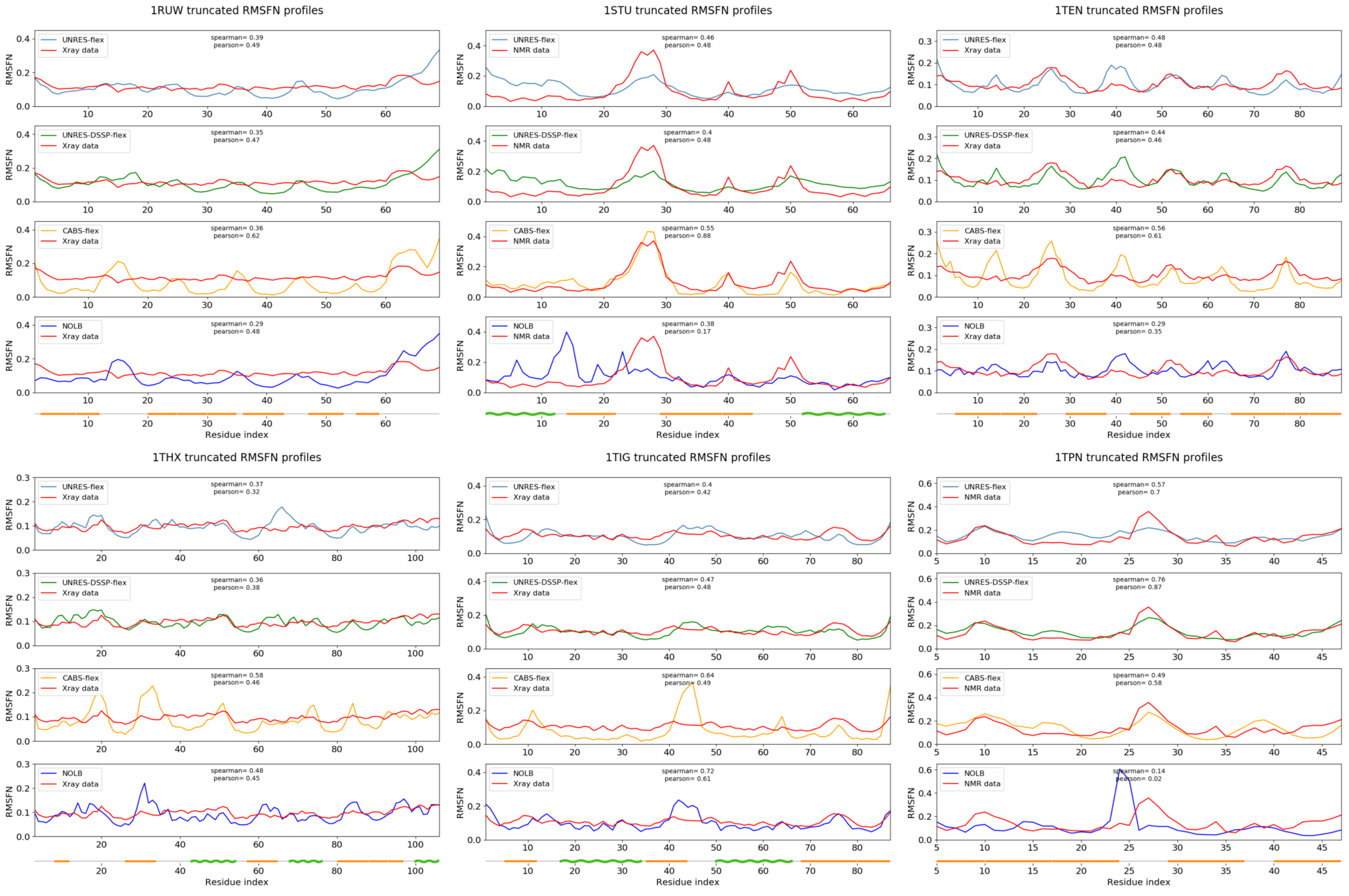

1RUW truncated RMSFN profiles
UNRES-flex
Xray data
spearman= 0.39
pearson= 0.49
UNRES-DSSP-flex
Xray data
spearman= 0.35
pearson= 0.47
CABS-flex
Xray data
spearman= 0.36
pearson= 0.62
NOLB
Xray data
spearman= 0.29
pearson= 0.48
1STU truncated RMSFN profiles
UNRES-flex
NMR data
spearman= 0.46
pearson= 0.48
UNRES-DSSP-flex
NMR data
spearman= 0.4
pearson= 0.48
CABS-flex
NMR data
spearman= 0.55
pearson= 0.88
NOLB
NMR data
spearman= 0.38
pearson= 0.17
1TEN truncated RMSFN profiles
UNRES-flex
Xray data
spearman= 0.48
pearson= 0.48
UNRES-DSSP-flex
Xray data
spearman= 0.44
pearson= 0.46
CABS-flex
Xray data
spearman= 0.56
pearson= 0.61
NOLB
Xray data
spearman= 0.29
pearson= 0.35
1THX truncated RMSFN profiles
UNRES-flex
Xray data
spearman= 0.37
pearson= 0.32
UNRES-DSSP-flex
Xray data
spearman= 0.36
pearson= 0.38
CABS-flex
Xray data
spearman= 0.58
pearson= 0.46
NOLB
Xray data
spearman= 0.48
pearson= 0.45
1TIG truncated RMSFN profiles
UNRES-flex
Xray data
spearman= 0.4
pearson= 0.42
UNRES-DSSP-flex
Xray data
spearman= 0.47
pearson= 0.48
CABS-flex
Xray data
spearman= 0.64
pearson= 0.49
NOLB
Xray data
spearman= 0.72
pearson= 0.61
1TPN truncated RMSFN profiles
UNRES-flex
NMR data
spearman= 0.57
pearson= 0.7
UNRES-DSSP-flex
NMR data
spearman= 0.76
pearson= 0.87
CABS-flex
NMR data
spearman= 0.49
pearson= 0.58
NOLB
NMR data
spearman= 0.14
pearson= 0.02
RMSFN
Residue index

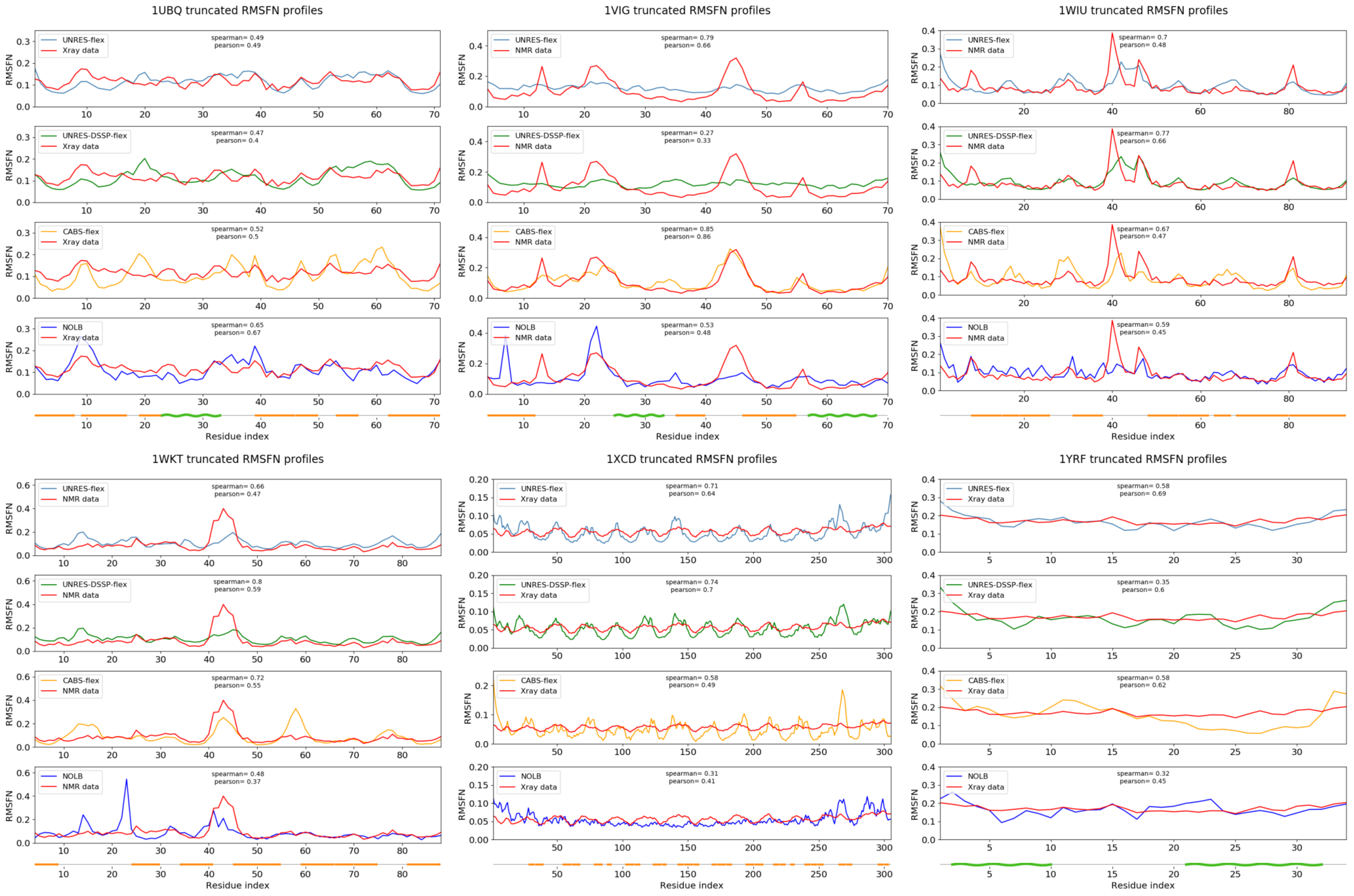
1UBQ truncated RMSFN profiles
UNRES-flex
Xray data
spearman= 0.49
pearson= 0.49
UNRES-DSSP-flex
Xray data
spearman= 0.47
pearson= 0.4
CABS-flex
Xray data
spearman= 0.52
pearson= 0.5
NOLB
Xray data
spearman= 0.65
pearson= 0.67
1VIG truncated RMSFN profiles
UNRES-flex
NMR data
spearman= 0.79
pearson= 0.66
UNRES-DSSP-flex
NMR data
spearman= 0.27
pearson= 0.33
CABS-flex
NMR data
spearman= 0.85
pearson= 0.86
NOLB
NMR data
spearman= 0.53
pearson= 0.48
1WIU truncated RMSFN profiles
UNRES-flex
NMR data
spearman= 0.7
pearson= 0.48
UNRES-DSSP-flex
NMR data
spearman= 0.77
pearson= 0.66
CABS-flex
NMR data
spearman= 0.67
pearson= 0.47
NOLB
NMR data
spearman= 0.59
pearson= 0.45
1WKT truncated RMSFN profiles
UNRES-flex
NMR data
spearman= 0.66
pearson= 0.47
UNRES-DSSP-flex
NMR data
spearman= 0.8
pearson= 0.59
CABS-flex
NMR data
spearman= 0.72
pearson= 0.55
NOLB
NMR data
spearman= 0.48
pearson= 0.37
1XCD truncated RMSFN profiles
UNRES-flex
Xray data
spearman= 0.71
pearson= 0.64
UNRES-DSSP-flex
Xray data
spearman= 0.74
pearson= 0.7
CABS-flex
Xray data
spearman= 0.58
pearson= 0.49
NOLB
Xray data
spearman= 0.31
pearson= 0.41
1YRF truncated RMSFN profiles
UNRES-flex
Xray data
spearman= 0.58
pearson= 0.69
UNRES-DSSP-flex
Xray data
spearman= 0.35
pearson= 0.6
CABS-flex
Xray data
spearman= 0.58
pearson= 0.62
NOLB
Xray data
spearman= 0.32
pearson= 0.45
RMSFN
Residue index

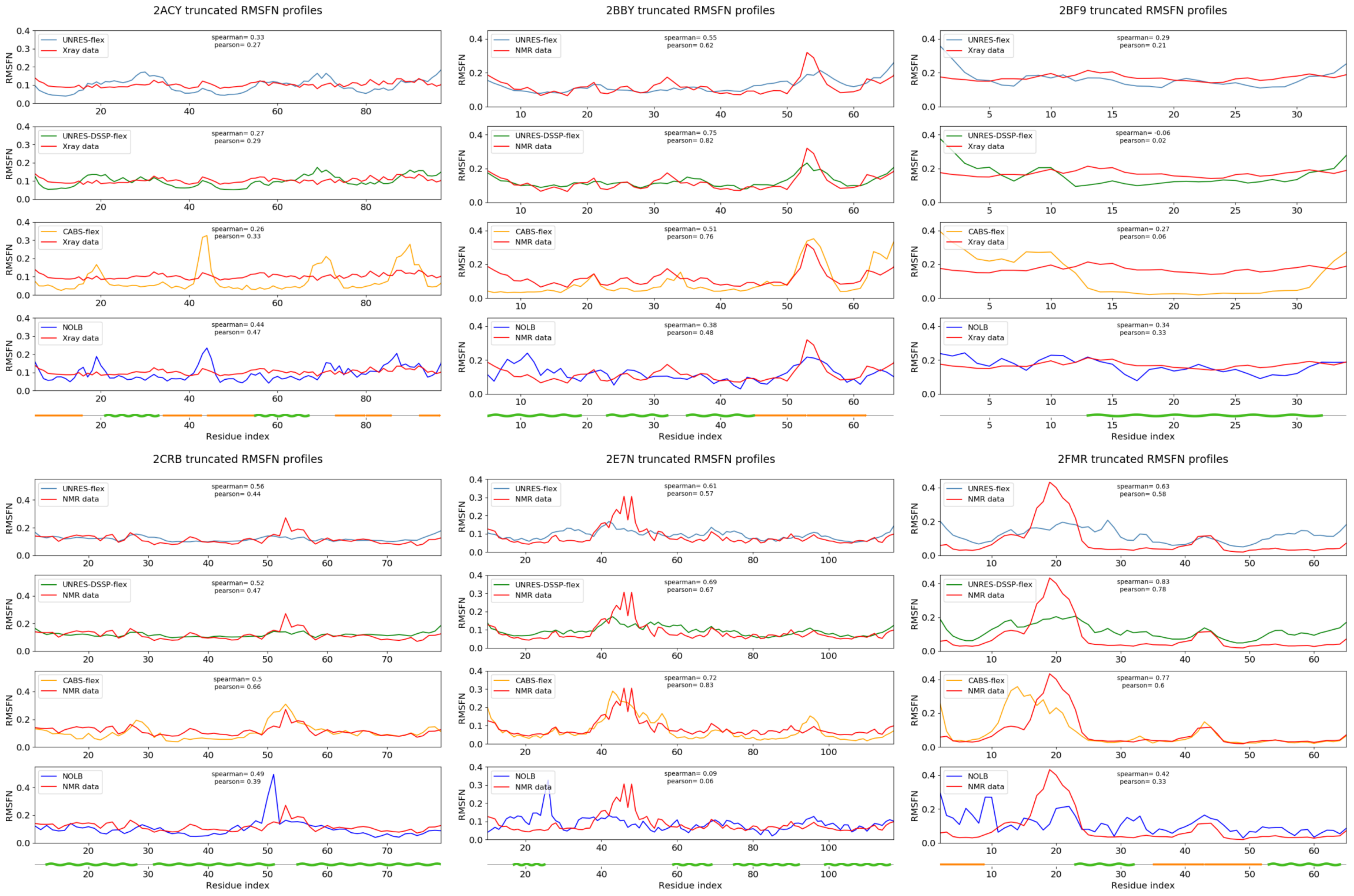

2ACY truncated RMSFN profiles
UNRES-flex
Xray data
spearman= 0.33
pearson= 0.27
UNRES-DSSP-flex
spearman= 0.27
pearson= 0.29
CABS-flex
spearman= 0.26
pearson= 0.33
NOLB
spearman= 0.44
pearson= 0.47
2BBY truncated RMSFN profiles
NMR data
spearman= 0.55
pearson= 0.62
spearman= 0.75
pearson= 0.82
spearman= 0.51
pearson= 0.76
spearman= 0.38
pearson= 0.48
2BF9 truncated RMSFN profiles
spearman= 0.29
pearson= 0.21
spearman= -0.06
pearson= 0.02
spearman= 0.27
pearson= 0.06
spearman= 0.34
pearson= 0.33
2CRB truncated RMSFN profiles
spearman= 0.56
pearson= 0.44
spearman= 0.52
pearson= 0.47
spearman= 0.5
pearson= 0.66
spearman= 0.49
pearson= 0.39
2E7N truncated RMSFN profiles
spearman= 0.61
pearson= 0.57
spearman= 0.69
pearson= 0.67
spearman= 0.72
pearson= 0.83
spearman= 0.09
pearson= 0.06
2FMR truncated RMSFN profiles
spearman= 0.63
pearson= 0.58
spearman= 0.83
pearson= 0.78
spearman= 0.77
pearson= 0.6
spearman= 0.42
pearson= 0.33
RMSFN
Residue index

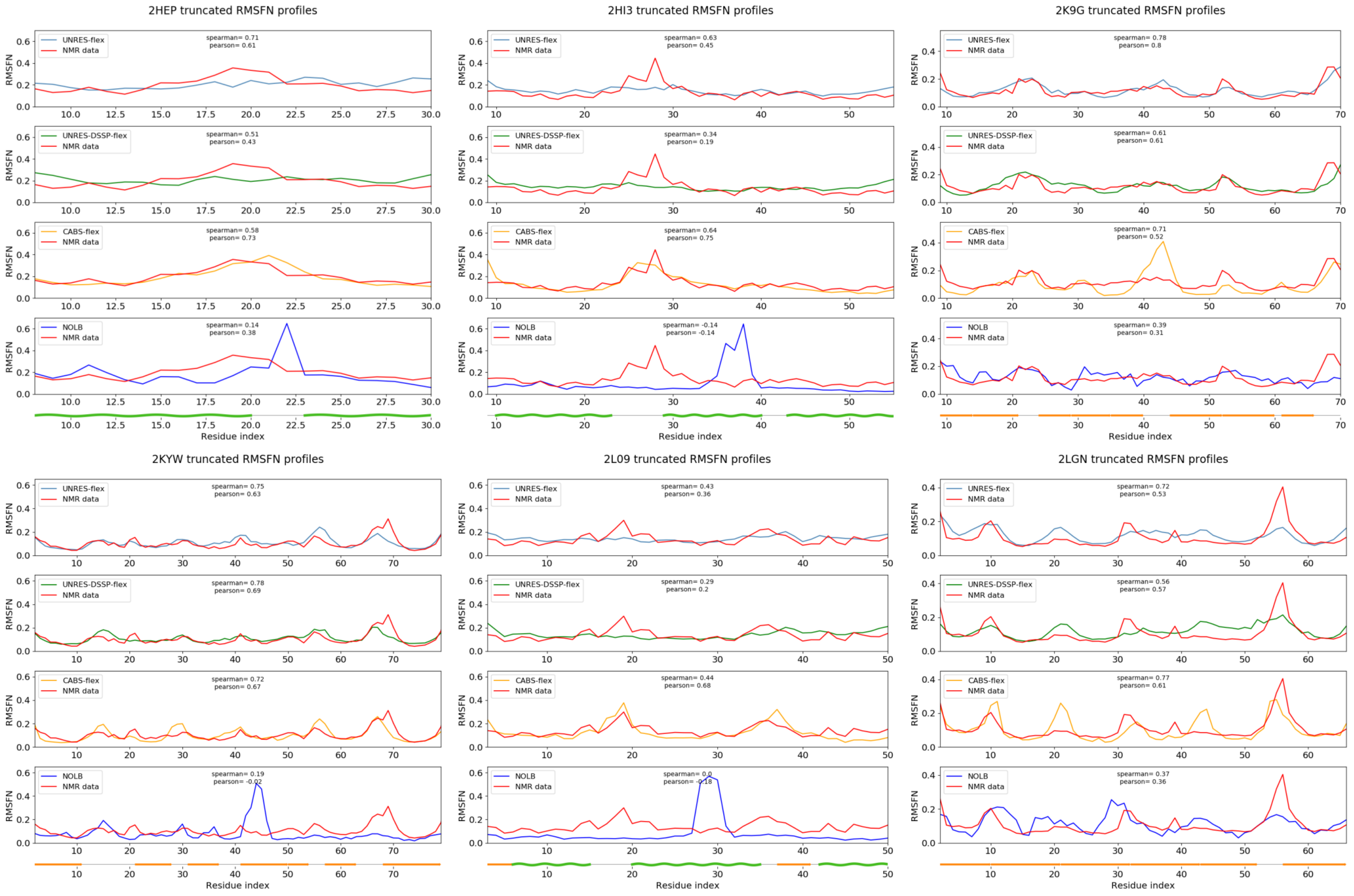

2HEP truncated RMSFN profiles
UNRES-flex
NMR data
spearman= 0.71
pearson= 0.61
UNRES-DSSP-flex
NMR data
spearman= 0.51
pearson= 0.43
CABS-flex
NMR data
spearman= 0.58
pearson= 0.73
NOLB
NMR data
spearman= 0.14
pearson= 0.38
2HI3 truncated RMSFN profiles
UNRES-flex
NMR data
spearman= 0.63
pearson= 0.45
UNRES-DSSP-flex
NMR data
spearman= 0.34
pearson= 0.19
CABS-flex
NMR data
spearman= 0.64
pearson= 0.75
NOLB
NMR data
spearman= -0.14
pearson= -0.14
2K9G truncated RMSFN profiles
UNRES-flex
NMR data
spearman= 0.78
pearson= 0.8
UNRES-DSSP-flex
NMR data
spearman= 0.61
pearson= 0.61
CABS-flex
NMR data
spearman= 0.71
pearson= 0.52
NOLB
NMR data
spearman= 0.39
pearson= 0.31
2KYW truncated RMSFN profiles
UNRES-flex
NMR data
spearman= 0.75
pearson= 0.63
UNRES-DSSP-flex
NMR data
spearman= 0.78
pearson= 0.69
CABS-flex
NMR data
spearman= 0.72
pearson= 0.67
NOLB
NMR data
spearman= 0.19
pearson= -0.02
2L09 truncated RMSFN profiles
UNRES-flex
NMR data
spearman= 0.43
pearson= 0.36
UNRES-DSSP-flex
NMR data
spearman= 0.29
pearson= 0.2
CABS-flex
NMR data
spearman= 0.44
pearson= 0.68
NOLB
NMR data
spearman= 0.0
pearson= -0.18
2LGN truncated RMSFN profiles
UNRES-flex
NMR data
spearman= 0.72
pearson= 0.53
UNRES-DSSP-flex
NMR data
spearman= 0.56
pearson= 0.57
CABS-flex
NMR data
spearman= 0.77
pearson= 0.61
NOLB
NMR data
spearman= 0.37
pearson= 0.36
RMSFN
Residue index

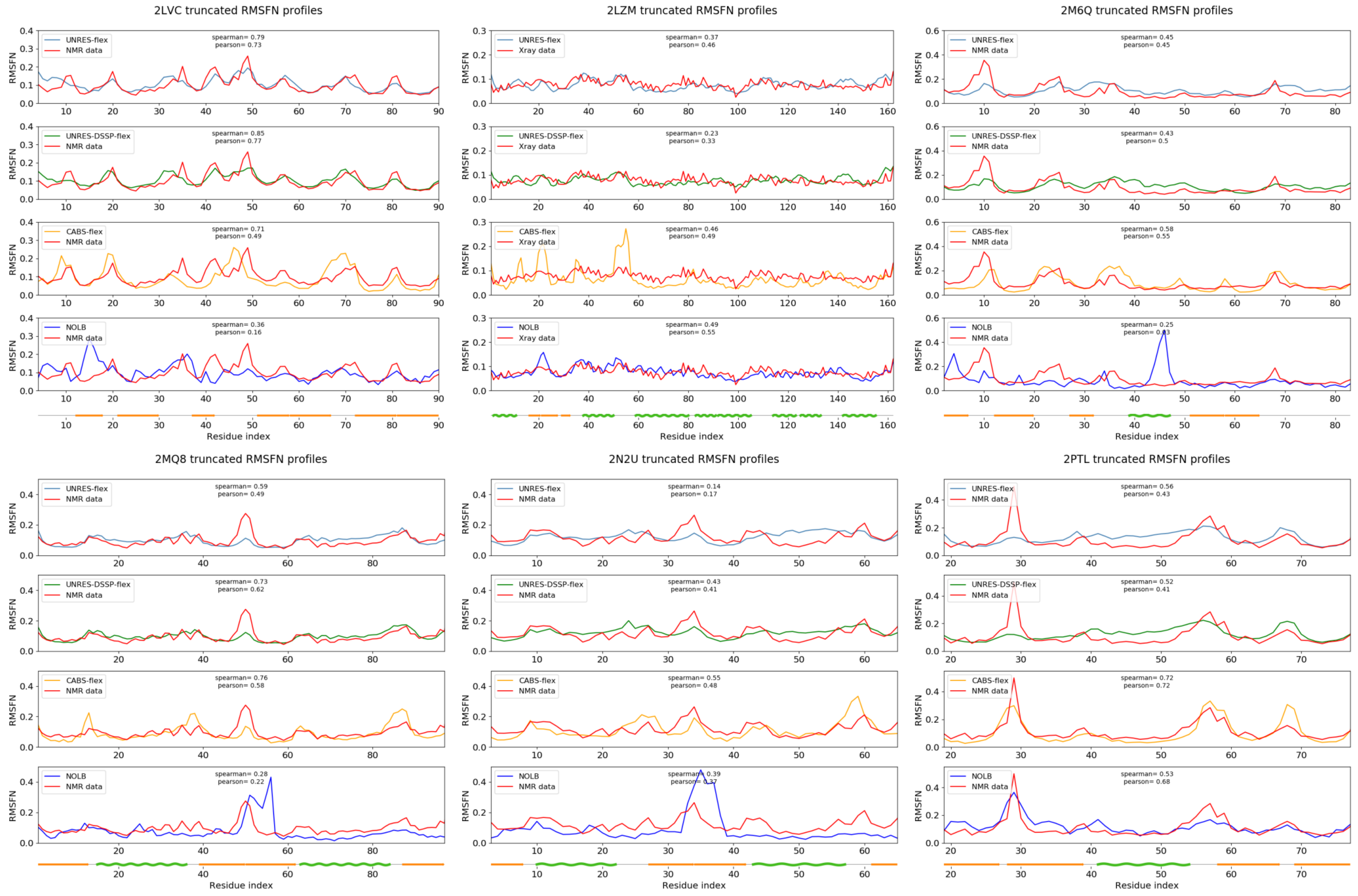

2LVC truncated RMSFN profiles
UNRES-flex
NMR data
spearman= 0.79
pearson= 0.73
UNRES-DSSP-flex
NMR data
spearman= 0.85
pearson= 0.77
CABS-flex
NMR data
spearman= 0.71
pearson= 0.49
NOLB
NMR data
spearman= 0.36
pearson= 0.16
2LZM truncated RMSFN profiles
UNRES-flex
Xray data
spearman= 0.37
pearson= 0.46
UNRES-DSSP-flex
Xray data
spearman= 0.23
pearson= 0.33
CABS-flex
Xray data
spearman= 0.46
pearson= 0.49
NOLB
Xray data
spearman= 0.49
pearson= 0.55
2M6Q truncated RMSFN profiles
UNRES-flex
NMR data
spearman= 0.45
pearson= 0.45
UNRES-DSSP-flex
NMR data
spearman= 0.43
pearson= 0.5
CABS-flex
NMR data
spearman= 0.58
pearson= 0.55
NOLB
NMR data
spearman= 0.25
2MQ8 truncated RMSFN profiles
UNRES-flex
NMR data
spearman= 0.59
pearson= 0.49
UNRES-DSSP-flex
NMR data
spearman= 0.73
pearson= 0.62
CABS-flex
NMR data
spearman= 0.76
pearson= 0.58
NOLB
NMR data
spearman= 0.28
pearson= 0.22
2N2U truncated RMSFN profiles
UNRES-flex
NMR data
spearman= 0.14
pearson= 0.17
UNRES-DSSP-flex
NMR data
spearman= 0.43
pearson= 0.41
CABS-flex
NMR data
spearman= 0.55
pearson= 0.48
NOLB
NMR data
2PTL truncated RMSFN profiles
UNRES-flex
NMR data
spearman= 0.56
pearson= 0.43
UNRES-DSSP-flex
NMR data
spearman= 0.52
pearson= 0.41
CABS-flex
NMR data
spearman= 0.72
pearson= 0.72
NOLB
NMR data
spearman= 0.53
pearson= 0.68
RMSFN
Residue index

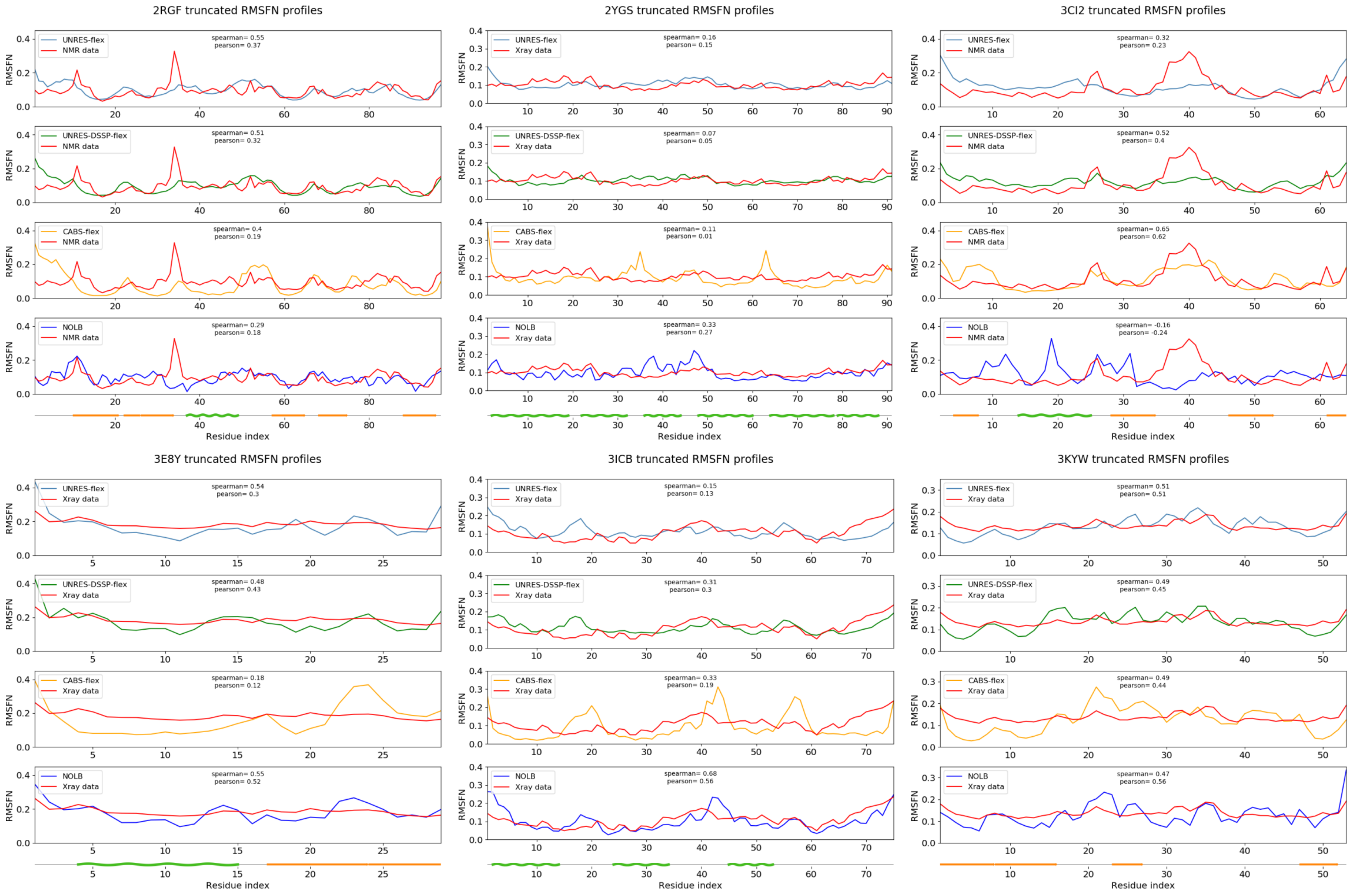

2RGF truncated RMSFN profiles
UNRES-flex
NMR data
spearman= 0.55
pearson= 0.37
UNRES-DSSP-flex
NMR data
spearman= 0.51
pearson= 0.32
CABS-flex
NMR data
spearman= 0.4
pearson= 0.19
NOLB
NMR data
spearman= 0.29
pearson= 0.18
2YGS truncated RMSFN profiles
UNRES-flex
Xray data
spearman= 0.16
pearson= 0.15
UNRES-DSSP-flex
Xray data
spearman= 0.07
pearson= 0.05
CABS-flex
Xray data
spearman= 0.11
pearson= 0.01
NOLB
Xray data
spearman= 0.33
pearson= 0.27
3CI2 truncated RMSFN profiles
UNRES-flex
NMR data
spearman= 0.32
pearson= 0.23
UNRES-DSSP-flex
NMR data
spearman= 0.52
pearson= 0.4
CABS-flex
NMR data
spearman= 0.65
pearson= 0.62
NOLB
NMR data
spearman= -0.16
pearson= -0.24
3E8Y truncated RMSFN profiles
UNRES-flex
Xray data
spearman= 0.54
pearson= 0.3
UNRES-DSSP-flex
Xray data
spearman= 0.48
pearson= 0.43
CABS-flex
Xray data
spearman= 0.18
pearson= 0.12
NOLB
Xray data
spearman= 0.55
pearson= 0.52
3ICB truncated RMSFN profiles
UNRES-flex
Xray data
spearman= 0.15
pearson= 0.13
UNRES-DSSP-flex
Xray data
spearman= 0.31
pearson= 0.3
CABS-flex
Xray data
spearman= 0.33
pearson= 0.19
NOLB
Xray data
spearman= 0.68
pearson= 0.56
3KYW truncated RMSFN profiles
UNRES-flex
Xray data
spearman= 0.51
pearson= 0.51
UNRES-DSSP-flex
Xray data
spearman= 0.49
pearson= 0.45
CABS-flex
Xray data
spearman= 0.49
pearson= 0.44
NOLB
Xray data
spearman= 0.47
pearson= 0.56
RMSFN
Residue index

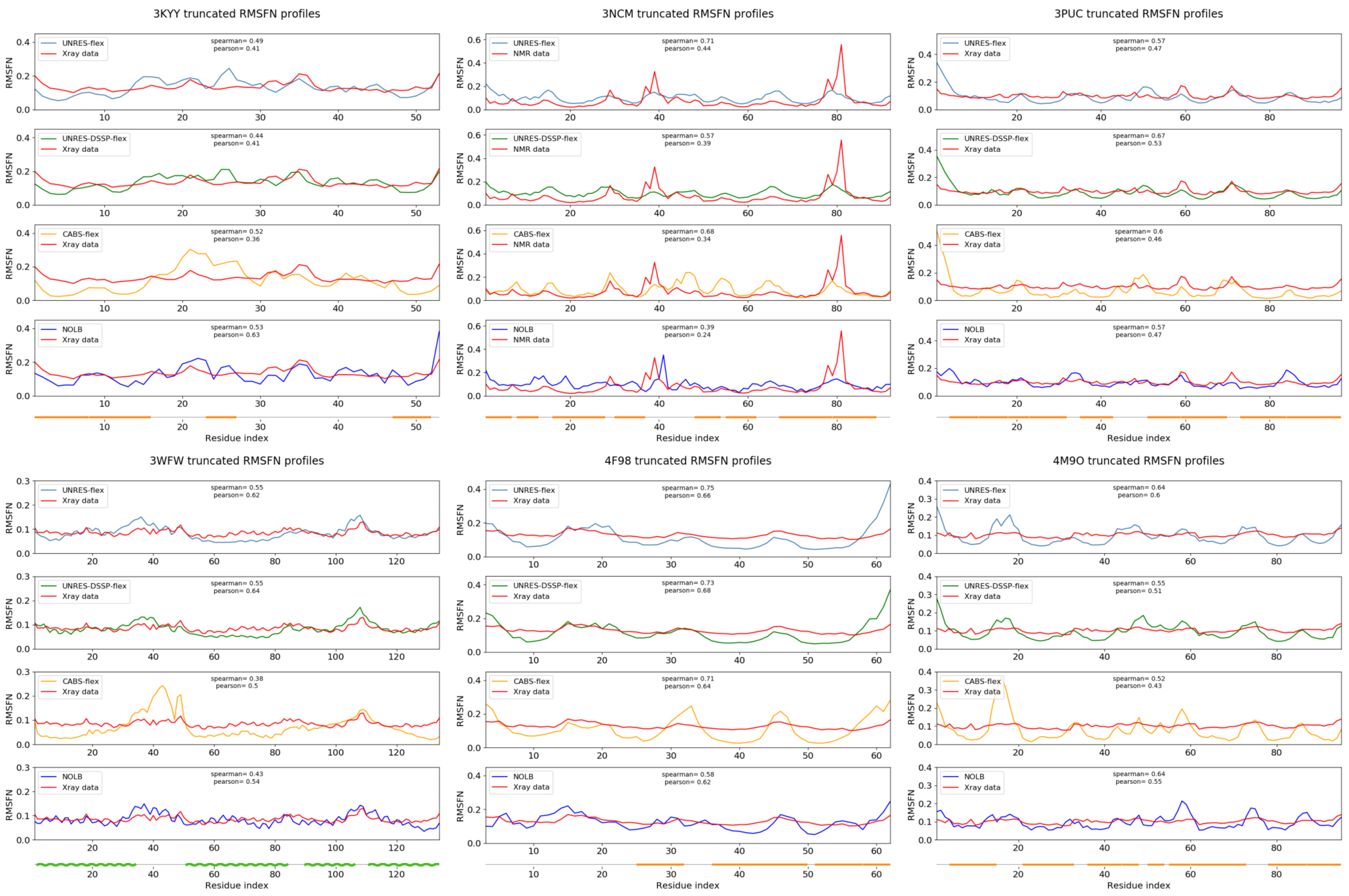
3KYY truncated RMSFN profiles
UNRES-flex
Xray data
spearman= 0.49
pearson= 0.41
UNRES-DSSP-flex
Xray data
spearman= 0.44
pearson= 0.41
CABS-flex
Xray data
spearman= 0.52
pearson= 0.36
NOLB
Xray data
spearman= 0.53
pearson= 0.63
3NCM truncated RMSFN profiles
UNRES-flex
NMR data
spearman= 0.71
pearson= 0.44
UNRES-DSSP-flex
NMR data
spearman= 0.57
pearson= 0.39
CABS-flex
NMR data
spearman= 0.68
pearson= 0.34
NOLB
NMR data
spearman= 0.39
pearson= 0.24
3PUC truncated RMSFN profiles
UNRES-flex
Xray data
spearman= 0.57
pearson= 0.47
UNRES-DSSP-flex
Xray data
spearman= 0.67
pearson= 0.53
CABS-flex
Xray data
spearman= 0.6
pearson= 0.46
NOLB
Xray data
spearman= 0.57
pearson= 0.47
3WFW truncated RMSFN profiles
UNRES-flex
Xray data
spearman= 0.55
pearson= 0.62
UNRES-DSSP-flex
Xray data
spearman= 0.55
pearson= 0.64
CABS-flex
Xray data
spearman= 0.38
pearson= 0.5
NOLB
Xray data
spearman= 0.43
pearson= 0.54
4F98 truncated RMSFN profiles
UNRES-flex
Xray data
spearman= 0.75
pearson= 0.66
UNRES-DSSP-flex
Xray data
spearman= 0.73
pearson= 0.68
CABS-flex
Xray data
spearman= 0.71
pearson= 0.64
NOLB
Xray data
spearman= 0.58
pearson= 0.62
4M9O truncated RMSFN profiles
UNRES-flex
Xray data
spearman= 0.64
pearson= 0.6
UNRES-DSSP-flex
Xray data
spearman= 0.55
pearson= 0.51
CABS-flex
Xray data
spearman= 0.52
pearson= 0.43
NOLB
Xray data
spearman= 0.64
pearson= 0.55
RMSFN
Residue index

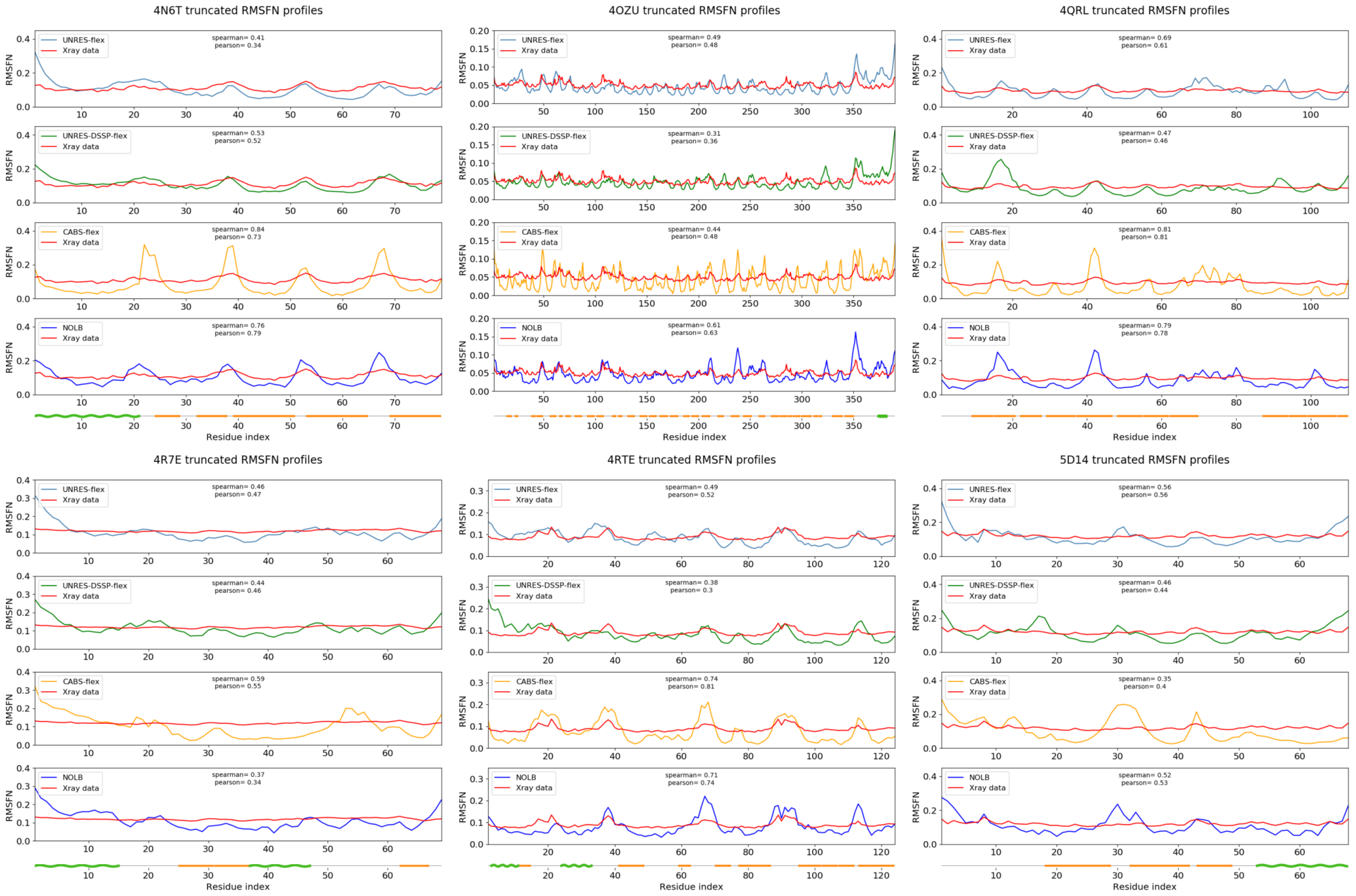
4N6T truncated RMSFN profiles
UNRES-flex
Xray data
spearman= 0.41
pearson= 0.34
UNRES-DSSP-flex
spearman= 0.53
pearson= 0.52
CABS-flex
spearman= 0.84
pearson= 0.73
NOLB
spearman= 0.76
pearson= 0.79
4OZU truncated RMSFN profiles
spearman= 0.49
pearson= 0.48
spearman= 0.31
pearson= 0.36
spearman= 0.44
pearson= 0.48
spearman= 0.61
pearson= 0.63
4QRL truncated RMSFN profiles
spearman= 0.69
pearson= 0.61
spearman= 0.47
pearson= 0.46
spearman= 0.81
pearson= 0.81
spearman= 0.79
pearson= 0.78
4R7E truncated RMSFN profiles
spearman= 0.46
pearson= 0.47
spearman= 0.44
pearson= 0.46
spearman= 0.59
pearson= 0.55
spearman= 0.37
pearson= 0.34
4RTE truncated RMSFN profiles
spearman= 0.49
pearson= 0.52
spearman= 0.38
pearson= 0.3
spearman= 0.74
pearson= 0.81
spearman= 0.71
pearson= 0.74
5D14 truncated RMSFN profiles
spearman= 0.56
pearson= 0.56
spearman= 0.46
pearson= 0.44
spearman= 0.35
pearson= 0.4
spearman= 0.52
pearson= 0.53
RMSFN
Residue index

6DNB truncated RMSFN profiles

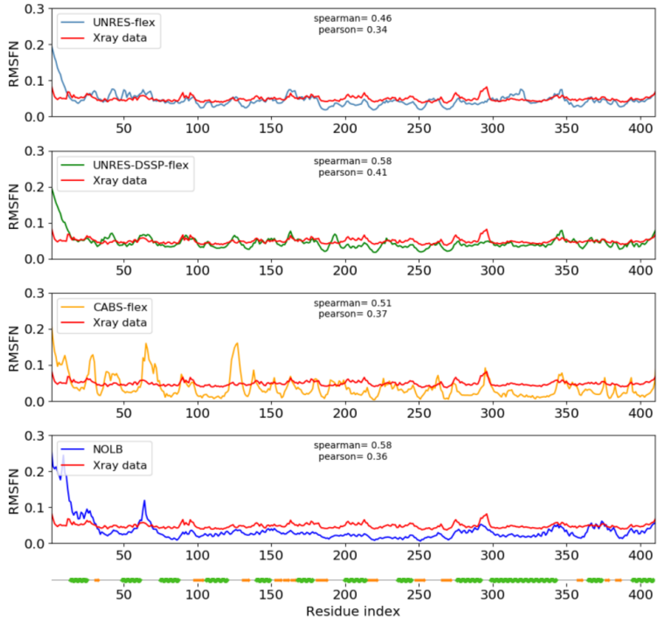

**Figure S2.** Normalized fluctuation profiles (RMSFN, dimensionless) calculated from NMR ensembles or X-ray B-factors (red line) and the corresponding profiled predicted by UNRES-flex (ligh-blue line), UNRES-DSSP-flex (green line), CABS-flex (yellow line), and NOLB (blue line). Except for NOLB, the fluctuation profiles are averaged over three simulations. The Pearson ($r_p$) and Spearman ($r_s$) coefficients for the correlation between the experimental and predicted profiles are shown in each panel. The solid orange and wave green lines below the abscissa of each panel indicate the β-sheet and α-helical structure, respectively. The analyses were carried out for full structures.

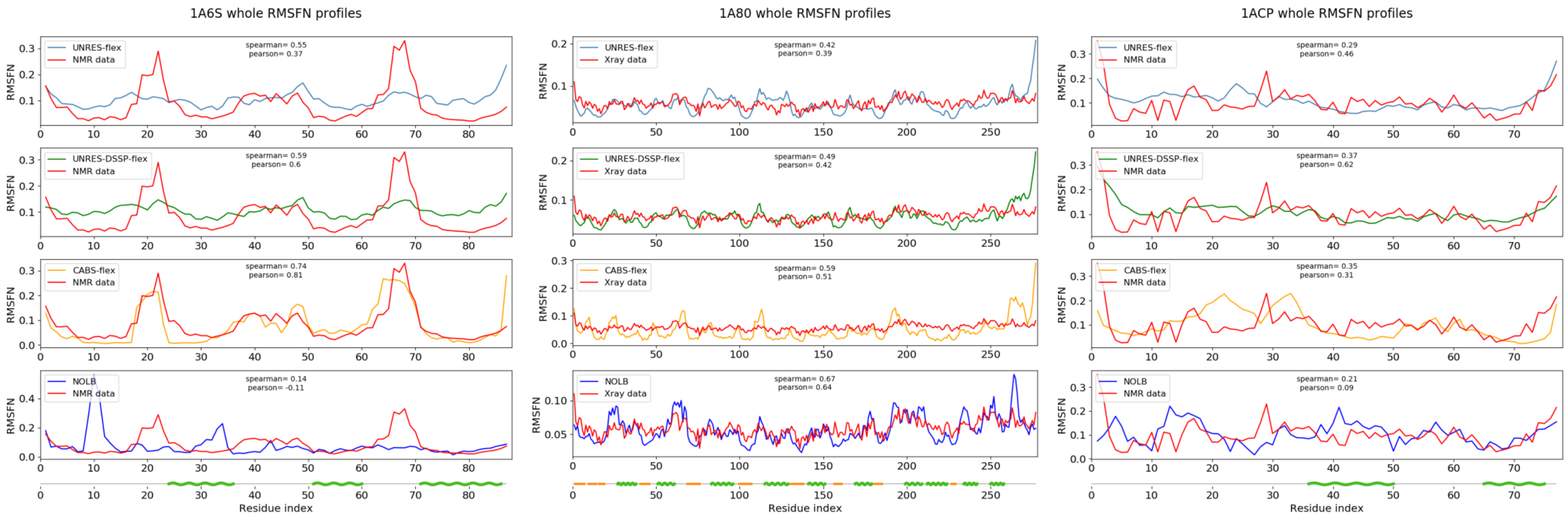

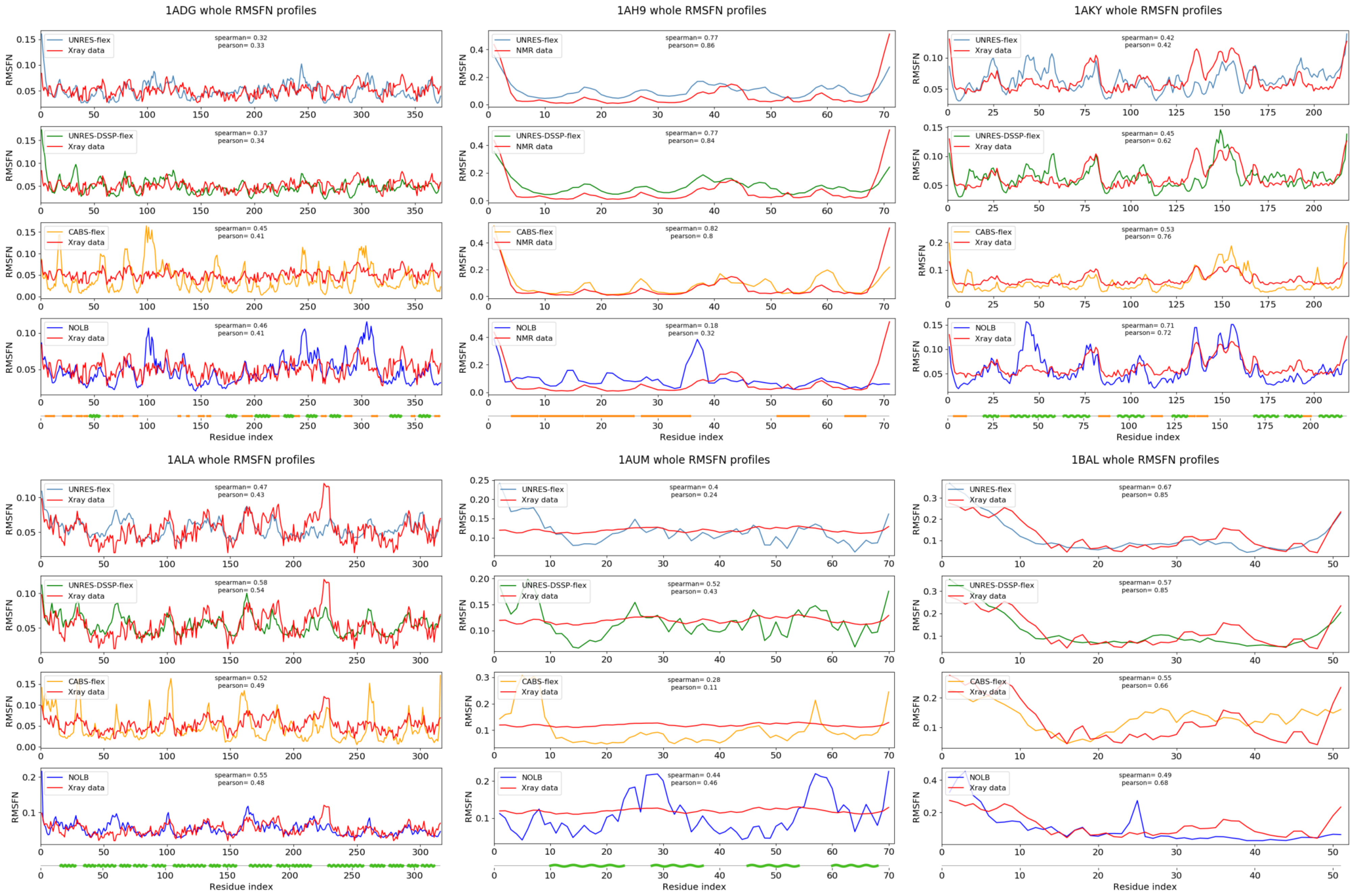

1ADG whole RMSFN profiles
UNRES-flex
Xray data
spearman= 0.32
pearson= 0.33
UNRES-DSSP-flex
Xray data
spearman= 0.37
pearson= 0.34
CABS-flex
Xray data
spearman= 0.45
pearson= 0.41
NOLB
Xray data
spearman= 0.46
pearson= 0.41
RMSFN
Residue index
1AH9 whole RMSFN profiles
UNRES-flex
NMR data
spearman= 0.77
pearson= 0.86
UNRES-DSSP-flex
NMR data
spearman= 0.77
pearson= 0.84
CABS-flex
NMR data
spearman= 0.82
pearson= 0.8
NOLB
NMR data
spearman= 0.18
pearson= 0.32
RMSFN
Residue index
1AKY whole RMSFN profiles
UNRES-flex
Xray data
spearman= 0.42
pearson= 0.42
UNRES-DSSP-flex
Xray data
spearman= 0.45
pearson= 0.62
CABS-flex
Xray data
spearman= 0.53
pearson= 0.76
NOLB
Xray data
spearman= 0.71
pearson= 0.72
RMSFN
Residue index
1ALA whole RMSFN profiles
UNRES-flex
Xray data
spearman= 0.47
pearson= 0.43
UNRES-DSSP-flex
Xray data
spearman= 0.58
pearson= 0.54
CABS-flex
Xray data
spearman= 0.52
pearson= 0.49
NOLB
Xray data
spearman= 0.55
pearson= 0.48
RMSFN
Residue index
1AUM whole RMSFN profiles
UNRES-flex
Xray data
spearman= 0.4
pearson= 0.24
UNRES-DSSP-flex
Xray data
spearman= 0.52
pearson= 0.43
CABS-flex
Xray data
spearman= 0.28
pearson= 0.11
NOLB
Xray data
spearman= 0.44
pearson= 0.46
RMSFN
Residue index
1BAL whole RMSFN profiles
UNRES-flex
Xray data
spearman= 0.67
pearson= 0.85
UNRES-DSSP-flex
Xray data
spearman= 0.57
pearson= 0.85
CABS-flex
Xray data
spearman= 0.55
pearson= 0.66
NOLB
Xray data
spearman= 0.49
pearson= 0.68
RMSFN
Residue index

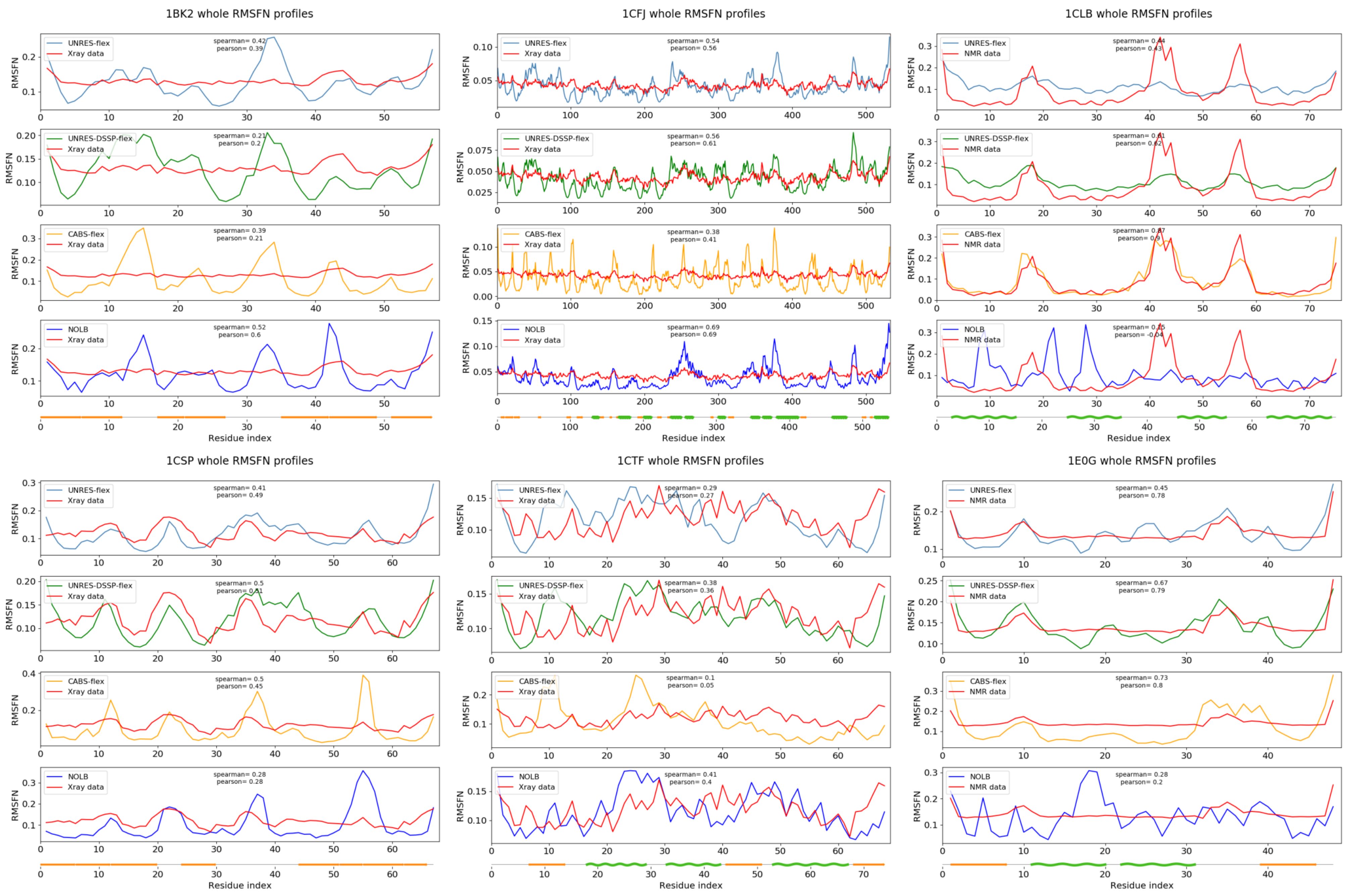
1BK2 whole RMSFN profiles
UNRES-flex
Xray data
spearman= 0.42
pearson= 0.39
UNRES-DSSP-flex
Xray data
spearman= 0.21
pearson= 0.2
CABS-flex
Xray data
spearman= 0.39
pearson= 0.21
NOLB
Xray data
spearman= 0.52
pearson= 0.6
1CFJ whole RMSFN profiles
UNRES-flex
Xray data
spearman= 0.54
pearson= 0.56
UNRES-DSSP-flex
Xray data
spearman= 0.56
pearson= 0.61
CABS-flex
Xray data
spearman= 0.38
pearson= 0.41
NOLB
Xray data
spearman= 0.69
pearson= 0.69
1CLB whole RMSFN profiles
UNRES-flex
NMR data
spearman= 0.44
pearson= 0.43
UNRES-DSSP-flex
NMR data
spearman= 0.61
pearson= 0.62
CABS-flex
NMR data
spearman= 0.87
pearson= 0.9
NOLB
NMR data
spearman= 0.15
pearson= -0.04
1CSP whole RMSFN profiles
UNRES-flex
Xray data
spearman= 0.41
pearson= 0.49
UNRES-DSSP-flex
Xray data
spearman= 0.5
pearson= 0.51
CABS-flex
Xray data
spearman= 0.5
pearson= 0.45
NOLB
Xray data
spearman= 0.28
pearson= 0.28
1CTF whole RMSFN profiles
UNRES-flex
Xray data
spearman= 0.29
pearson= 0.27
UNRES-DSSP-flex
Xray data
spearman= 0.38
pearson= 0.36
CABS-flex
Xray data
spearman= 0.1
pearson= 0.05
NOLB
Xray data
spearman= 0.41
pearson= 0.4
1E0G whole RMSFN profiles
UNRES-flex
NMR data
spearman= 0.45
pearson= 0.78
UNRES-DSSP-flex
NMR data
spearman= 0.67
pearson= 0.79
CABS-flex
NMR data
spearman= 0.73
pearson= 0.8
NOLB
NMR data
spearman= 0.28
pearson= 0.2
RMSFN
Residue index

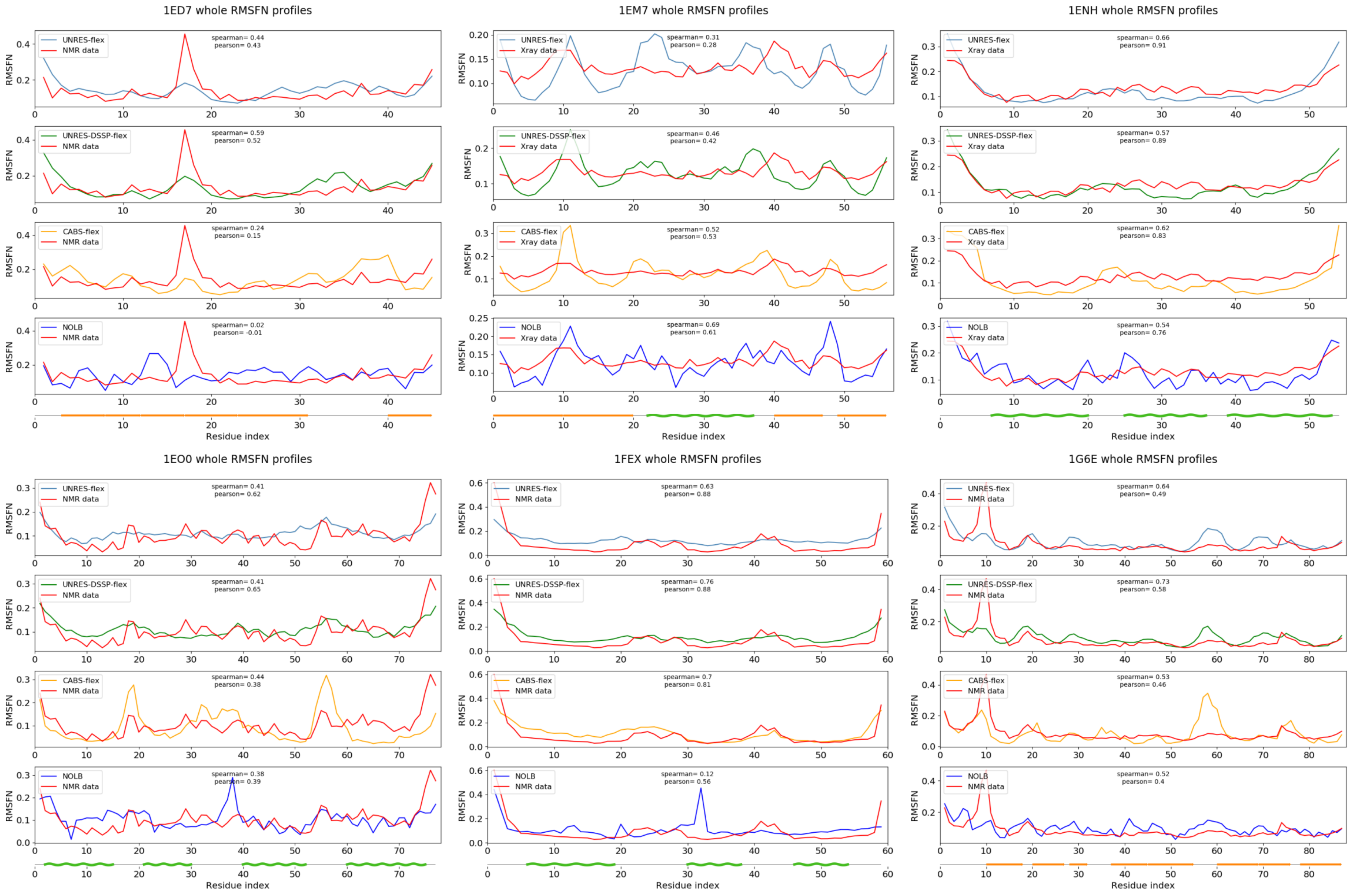
1ED7 whole RMSFN profiles
UNRES-flex
NMR data
spearman= 0.44
pearson= 0.43
UNRES-DSSP-flex
NMR data
spearman= 0.59
pearson= 0.52
CABS-flex
NMR data
spearman= 0.24
pearson= 0.15
NOLB
NMR data
spearman= 0.02
pearson= -0.01
Residue index
1EM7 whole RMSFN profiles
UNRES-flex
Xray data
spearman= 0.31
pearson= 0.28
UNRES-DSSP-flex
Xray data
spearman= 0.46
pearson= 0.42
CABS-flex
Xray data
spearman= 0.52
pearson= 0.53
NOLB
Xray data
spearman= 0.69
pearson= 0.61
Residue index
1ENH whole RMSFN profiles
UNRES-flex
Xray data
spearman= 0.66
pearson= 0.91
UNRES-DSSP-flex
Xray data
spearman= 0.57
pearson= 0.89
CABS-flex
Xray data
spearman= 0.62
pearson= 0.83
NOLB
Xray data
spearman= 0.54
pearson= 0.76
Residue index
1EO0 whole RMSFN profiles
UNRES-flex
NMR data
spearman= 0.41
pearson= 0.62
UNRES-DSSP-flex
NMR data
spearman= 0.41
pearson= 0.65
CABS-flex
NMR data
spearman= 0.44
pearson= 0.38
NOLB
NMR data
spearman= 0.38
pearson= 0.39
Residue index
1FEX whole RMSFN profiles
UNRES-flex
NMR data
spearman= 0.63
pearson= 0.88
UNRES-DSSP-flex
NMR data
spearman= 0.76
pearson= 0.88
CABS-flex
NMR data
spearman= 0.7
pearson= 0.81
NOLB
NMR data
spearman= 0.12
pearson= 0.56
Residue index
1G6E whole RMSFN profiles
UNRES-flex
NMR data
spearman= 0.64
pearson= 0.49
UNRES-DSSP-flex
NMR data
spearman= 0.73
pearson= 0.58
CABS-flex
NMR data
spearman= 0.53
pearson= 0.46
NOLB
NMR data
spearman= 0.52
pearson= 0.4
Residue index
RMSFN

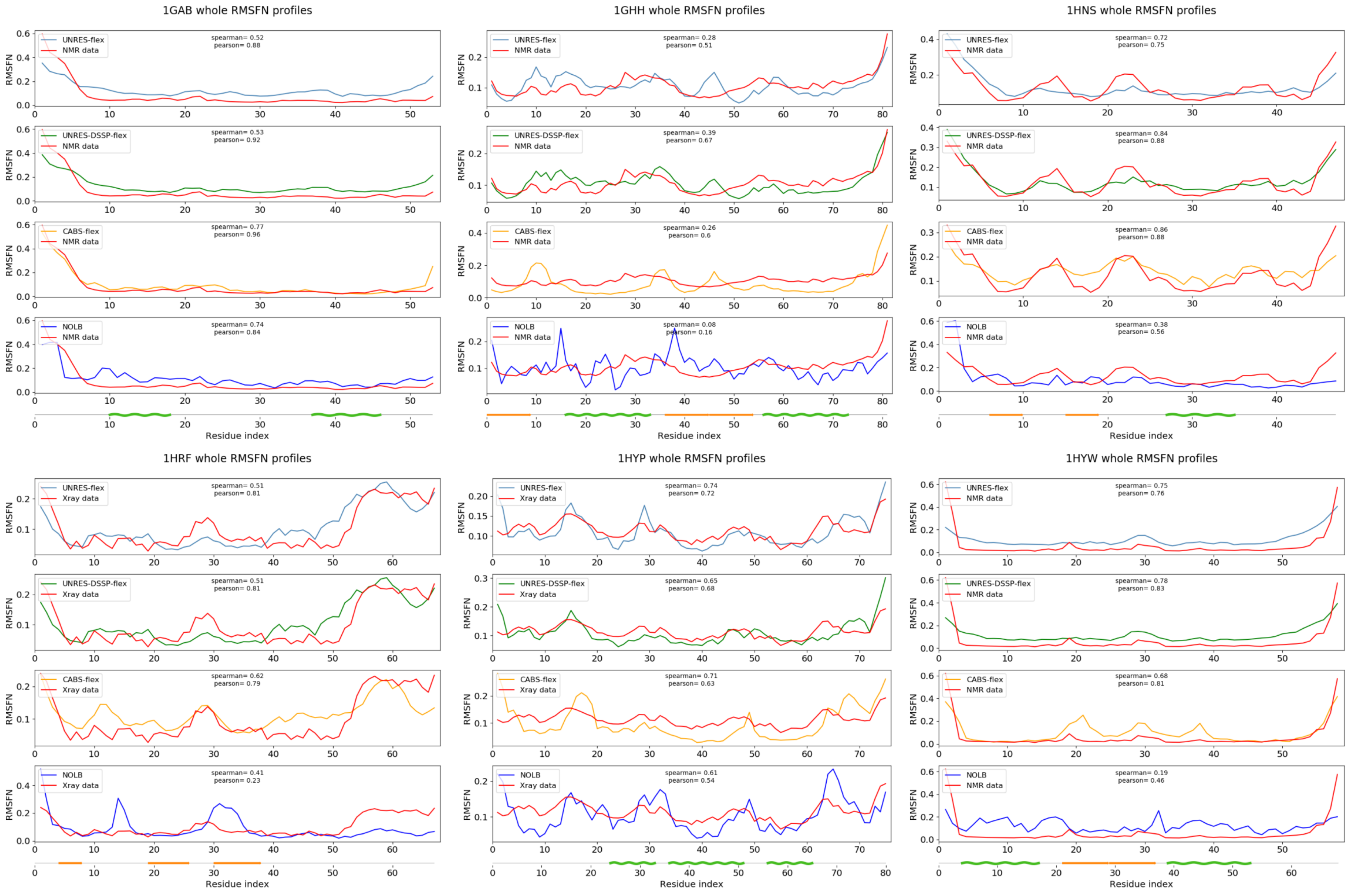

1GAB whole RMSFN profiles
UNRES-flex
NMR data
spearman= 0.52
pearson= 0.88
UNRES-DSSP-flex
NMR data
spearman= 0.53
pearson= 0.92
CABS-flex
NMR data
spearman= 0.77
pearson= 0.96
NOLB
NMR data
spearman= 0.74
pearson= 0.84
1GHH whole RMSFN profiles
UNRES-flex
NMR data
spearman= 0.28
pearson= 0.51
UNRES-DSSP-flex
NMR data
spearman= 0.39
pearson= 0.67
CABS-flex
NMR data
spearman= 0.26
pearson= 0.6
NOLB
NMR data
spearman= 0.08
pearson= 0.16
1HNS whole RMSFN profiles
UNRES-flex
NMR data
spearman= 0.72
pearson= 0.75
UNRES-DSSP-flex
NMR data
spearman= 0.84
pearson= 0.88
CABS-flex
NMR data
spearman= 0.86
pearson= 0.88
NOLB
NMR data
spearman= 0.38
pearson= 0.56
1HRF whole RMSFN profiles
UNRES-flex
Xray data
spearman= 0.51
pearson= 0.81
UNRES-DSSP-flex
Xray data
spearman= 0.51
pearson= 0.81
CABS-flex
Xray data
spearman= 0.62
pearson= 0.79
NOLB
Xray data
spearman= 0.41
pearson= 0.23
1HYP whole RMSFN profiles
UNRES-flex
Xray data
spearman= 0.74
pearson= 0.72
UNRES-DSSP-flex
Xray data
spearman= 0.65
pearson= 0.68
CABS-flex
Xray data
spearman= 0.71
pearson= 0.63
NOLB
Xray data
spearman= 0.61
pearson= 0.54
1HYW whole RMSFN profiles
UNRES-flex
NMR data
spearman= 0.75
pearson= 0.76
UNRES-DSSP-flex
NMR data
spearman= 0.78
pearson= 0.83
CABS-flex
NMR data
spearman= 0.68
pearson= 0.81
NOLB
NMR data
spearman= 0.19
pearson= 0.46
RMSFN
Residue index

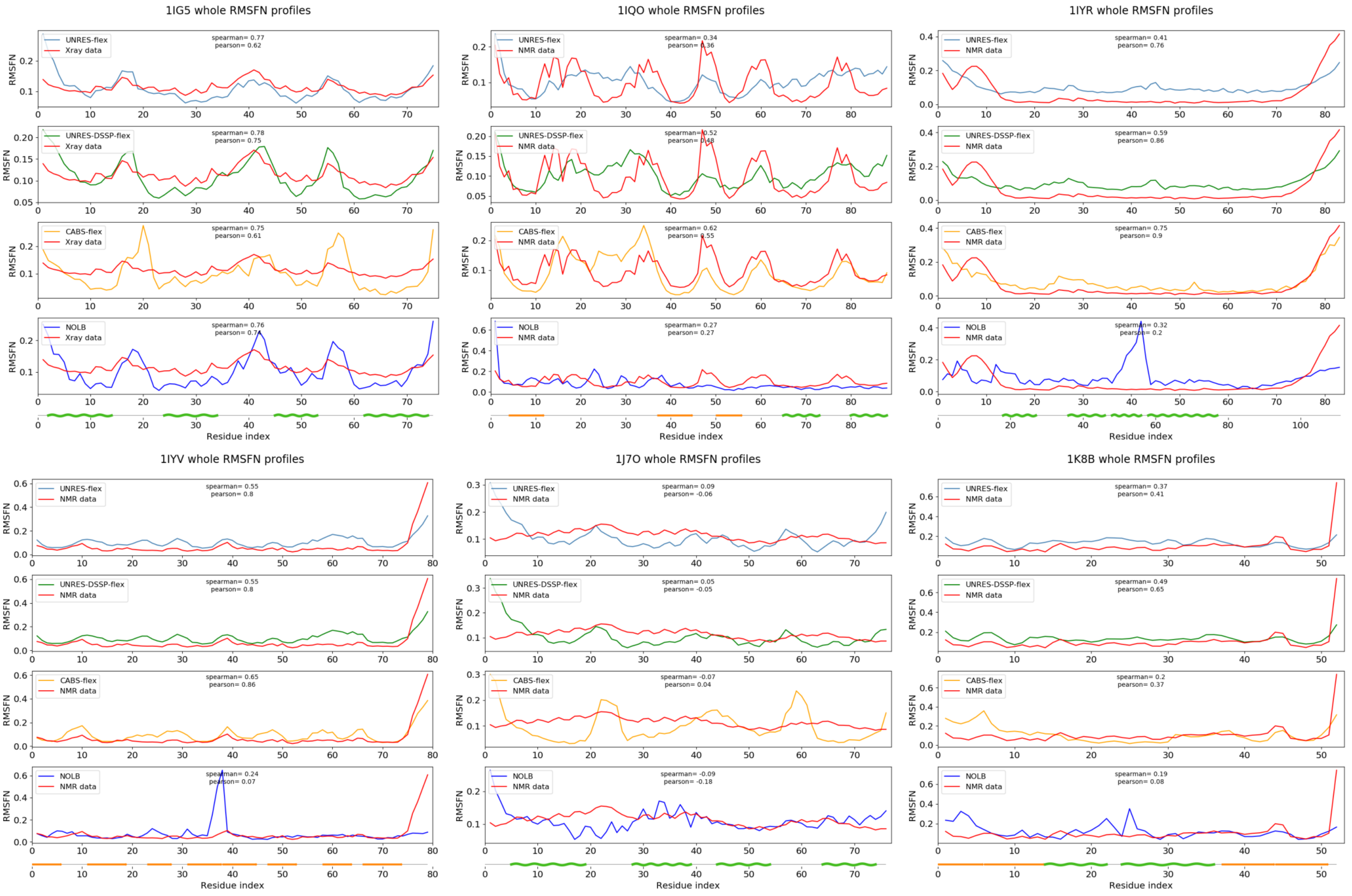

1IG5 whole RMSFN profiles
UNRES-flex
Xray data
spearman= 0.77
pearson= 0.62
UNRES-DSSP-flex
Xray data
spearman= 0.78
pearson= 0.75
CABS-flex
Xray data
spearman= 0.75
pearson= 0.61
NOLB
Xray data
spearman= 0.76
1IQO whole RMSFN profiles
UNRES-flex
NMR data
spearman= 0.34
UNRES-DSSP-flex
NMR data
CABS-flex
NMR data
spearman= 0.62
NOLB
NMR data
spearman= 0.27
pearson= 0.27
1IYR whole RMSFN profiles
UNRES-flex
NMR data
spearman= 0.41
pearson= 0.76
UNRES-DSSP-flex
NMR data
spearman= 0.59
pearson= 0.86
CABS-flex
NMR data
spearman= 0.75
pearson= 0.9
NOLB
NMR data
spearman= 0.32
1IYV whole RMSFN profiles
UNRES-flex
NMR data
spearman= 0.55
pearson= 0.8
UNRES-DSSP-flex
NMR data
spearman= 0.55
pearson= 0.8
CABS-flex
NMR data
spearman= 0.65
pearson= 0.86
NOLB
NMR data
1J7O whole RMSFN profiles
UNRES-flex
NMR data
spearman= 0.09
pearson= -0.06
UNRES-DSSP-flex
NMR data
spearman= 0.05
pearson= -0.05
CABS-flex
NMR data
spearman= -0.07
pearson= 0.04
NOLB
NMR data
spearman= -0.09
pearson= -0.18
1K8B whole RMSFN profiles
UNRES-flex
NMR data
spearman= 0.37
pearson= 0.41
UNRES-DSSP-flex
NMR data
spearman= 0.49
pearson= 0.65
CABS-flex
NMR data
spearman= 0.2
pearson= 0.37
NOLB
NMR data
spearman= 0.19
pearson= 0.08
RMSFN
Residue index

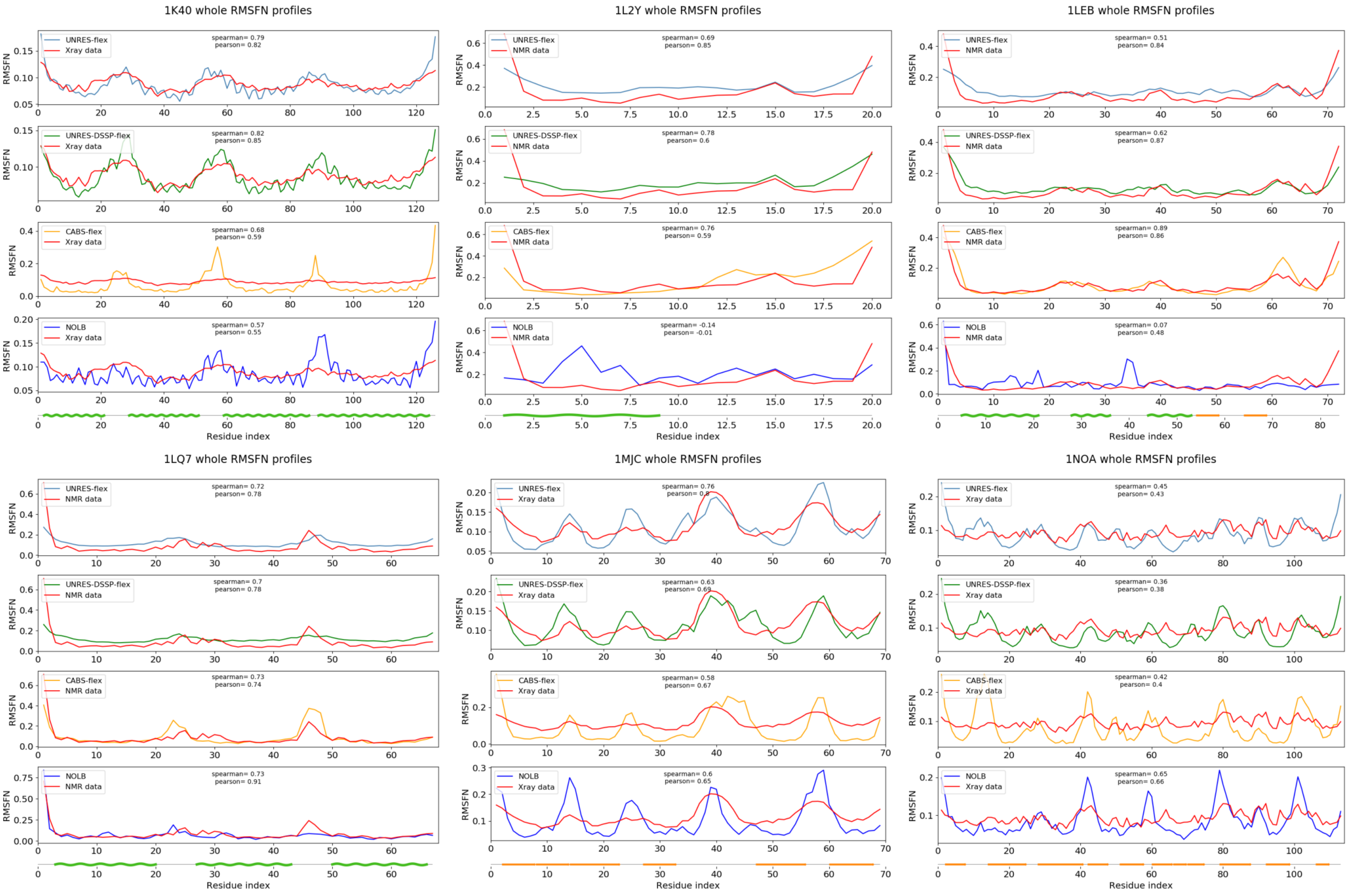

1K40 whole RMSFN profiles
1L2Y whole RMSFN profiles
1LEB whole RMSFN profiles
UNRES-flex
Xray data
spearman= 0.79
pearson= 0.82
UNRES-DSSP-flex
spearman= 0.82
pearson= 0.85
CABS-flex
spearman= 0.68
pearson= 0.59
NOLB
spearman= 0.57
pearson= 0.55
NMR data
spearman= 0.69
pearson= 0.85
spearman= 0.78
pearson= 0.6
spearman= 0.76
pearson= 0.59
spearman= -0.14
pearson= -0.01
spearman= 0.51
pearson= 0.84
spearman= 0.62
pearson= 0.87
spearman= 0.89
pearson= 0.86
spearman= 0.07
pearson= 0.48
RMSFN
Residue index
1LQ7 whole RMSFN profiles
1MJC whole RMSFN profiles
1NOA whole RMSFN profiles
spearman= 0.72
pearson= 0.78
spearman= 0.7
pearson= 0.78
spearman= 0.73
pearson= 0.74
spearman= 0.73
pearson= 0.91
spearman= 0.76
pearson= 0.8
spearman= 0.63
pearson= 0.69
spearman= 0.58
pearson= 0.67
spearman= 0.6
pearson= 0.65
spearman= 0.45
pearson= 0.43
spearman= 0.36
pearson= 0.38
spearman= 0.42
pearson= 0.4
spearman= 0.65
pearson= 0.66

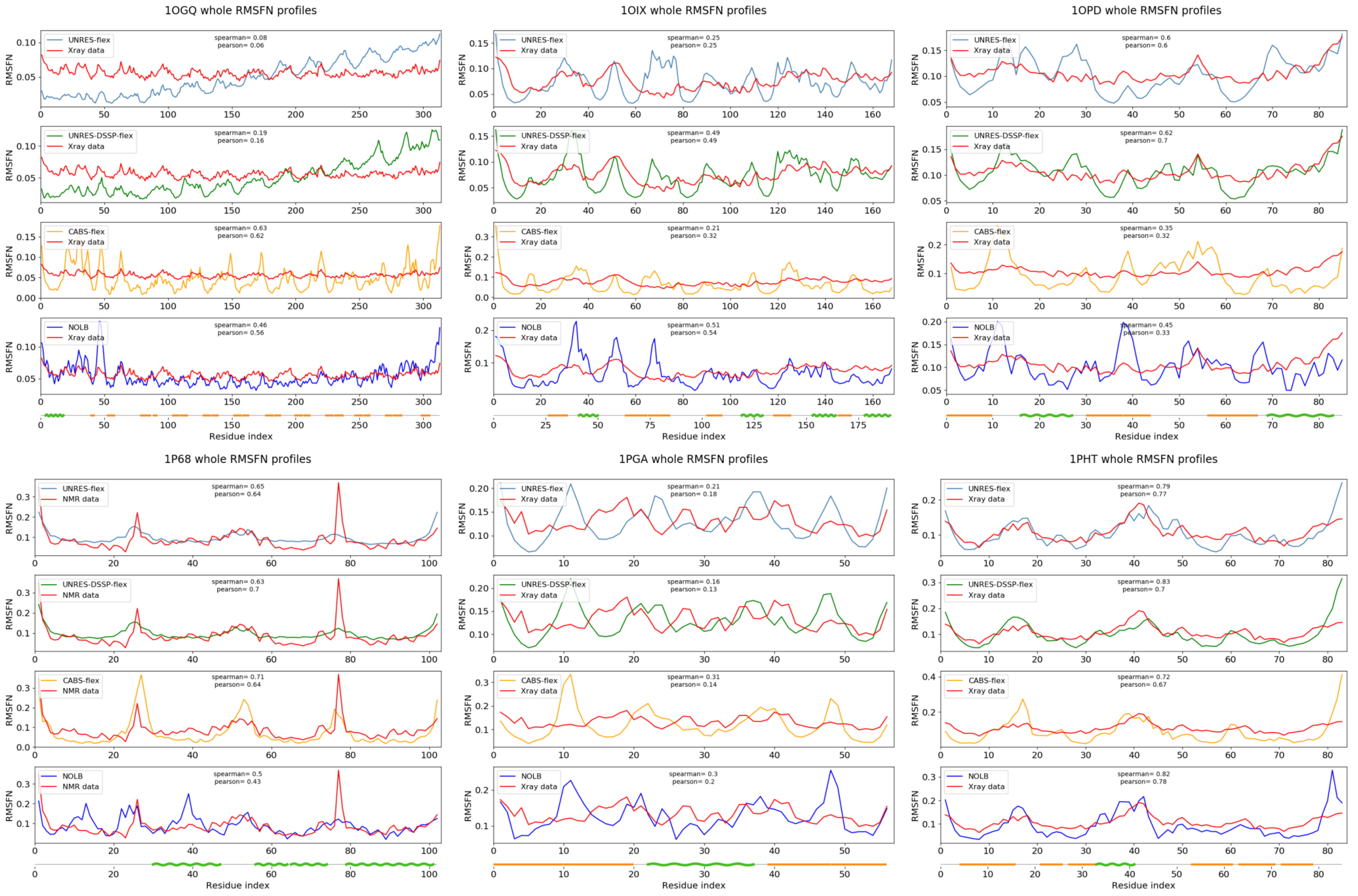
1OGQ whole RMSFN profiles
UNRES-flex
Xray data
spearman= 0.08
pearson= 0.06
UNRES-DSSP-flex
Xray data
spearman= 0.19
pearson= 0.16
CABS-flex
Xray data
spearman= 0.63
pearson= 0.62
NOLB
Xray data
spearman= 0.46
pearson= 0.56
1OIX whole RMSFN profiles
UNRES-flex
Xray data
spearman= 0.25
pearson= 0.25
UNRES-DSSP-flex
Xray data
spearman= 0.49
pearson= 0.49
CABS-flex
Xray data
spearman= 0.21
pearson= 0.32
NOLB
Xray data
spearman= 0.51
pearson= 0.54
1OPD whole RMSFN profiles
UNRES-flex
Xray data
spearman= 0.6
pearson= 0.6
UNRES-DSSP-flex
Xray data
spearman= 0.62
pearson= 0.7
CABS-flex
Xray data
spearman= 0.35
pearson= 0.32
NOLB
Xray data
spearman= 0.45
pearson= 0.33
1P68 whole RMSFN profiles
UNRES-flex
NMR data
spearman= 0.65
pearson= 0.64
UNRES-DSSP-flex
NMR data
spearman= 0.63
pearson= 0.7
CABS-flex
NMR data
spearman= 0.71
pearson= 0.64
NOLB
NMR data
spearman= 0.5
pearson= 0.43
1PGA whole RMSFN profiles
UNRES-flex
Xray data
spearman= 0.21
pearson= 0.18
UNRES-DSSP-flex
Xray data
spearman= 0.16
pearson= 0.13
CABS-flex
Xray data
spearman= 0.31
pearson= 0.14
NOLB
Xray data
spearman= 0.3
pearson= 0.2
1PHT whole RMSFN profiles
UNRES-flex
Xray data
spearman= 0.79
pearson= 0.77
UNRES-DSSP-flex
Xray data
spearman= 0.83
pearson= 0.7
CABS-flex
Xray data
spearman= 0.72
pearson= 0.67
NOLB
Xray data
spearman= 0.82
pearson= 0.78
RMSFN
Residue index

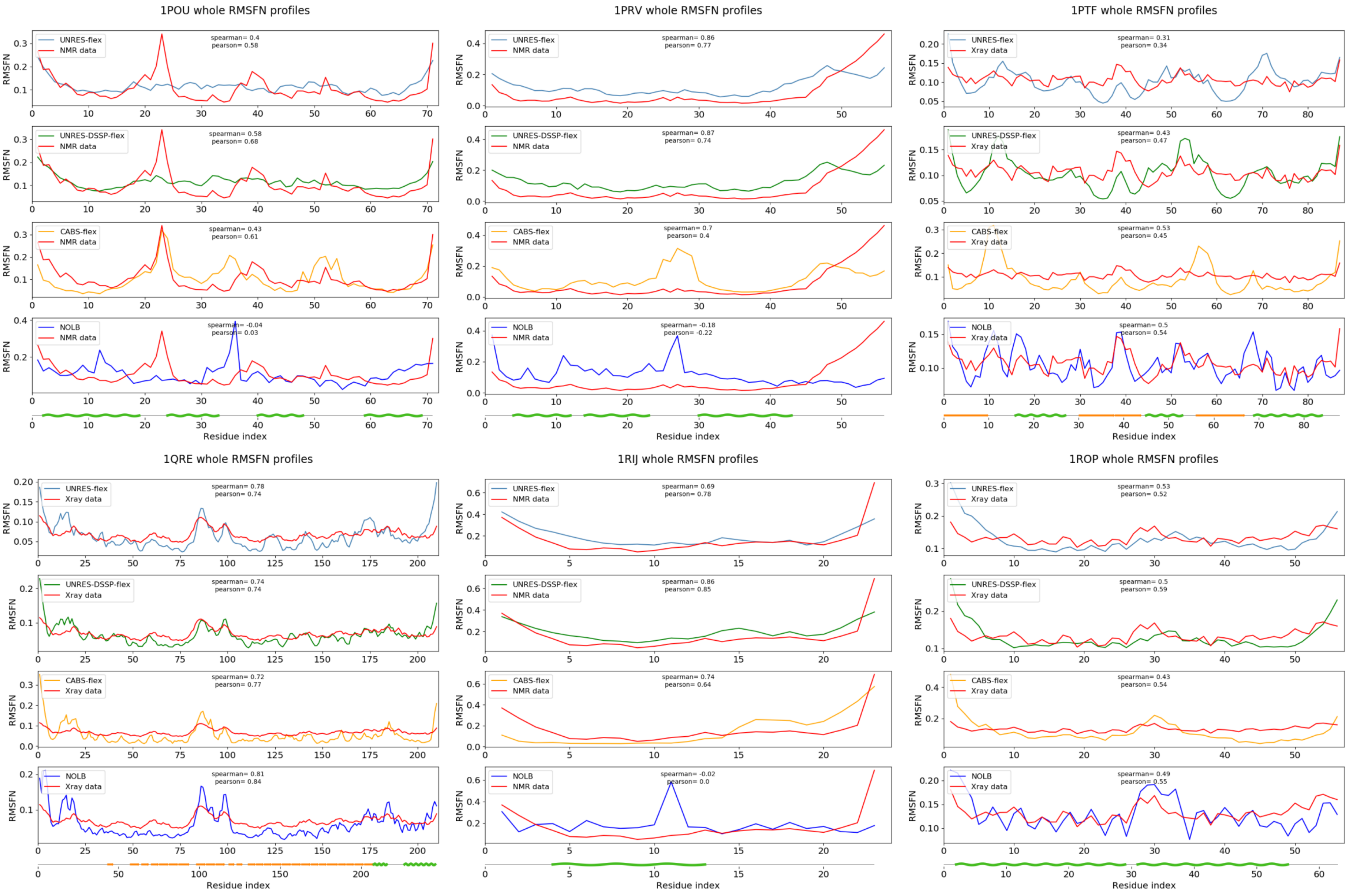
1POU whole RMSFN profiles
UNRES-flex
NMR data
spearman= 0.4
pearson= 0.58
UNRES-DSSP-flex
NMR data
spearman= 0.58
pearson= 0.68
CABS-flex
NMR data
spearman= 0.43
pearson= 0.61
NOLB
NMR data
spearman= -0.04
pearson= 0.03
1PRV whole RMSFN profiles
UNRES-flex
NMR data
spearman= 0.86
pearson= 0.77
UNRES-DSSP-flex
NMR data
spearman= 0.87
pearson= 0.74
CABS-flex
NMR data
spearman= 0.7
pearson= 0.4
NOLB
NMR data
spearman= -0.18
pearson= -0.22
1PTF whole RMSFN profiles
UNRES-flex
Xray data
spearman= 0.31
pearson= 0.34
UNRES-DSSP-flex
Xray data
spearman= 0.43
pearson= 0.47
CABS-flex
Xray data
spearman= 0.53
pearson= 0.45
NOLB
Xray data
spearman= 0.5
pearson= 0.54
1QRE whole RMSFN profiles
UNRES-flex
Xray data
spearman= 0.78
pearson= 0.74
UNRES-DSSP-flex
Xray data
spearman= 0.74
pearson= 0.74
CABS-flex
Xray data
spearman= 0.72
pearson= 0.77
NOLB
Xray data
spearman= 0.81
pearson= 0.84
1RIJ whole RMSFN profiles
UNRES-flex
NMR data
spearman= 0.69
pearson= 0.78
UNRES-DSSP-flex
NMR data
spearman= 0.86
pearson= 0.85
CABS-flex
NMR data
spearman= 0.74
pearson= 0.64
NOLB
NMR data
spearman= -0.02
pearson= 0.0
1ROP whole RMSFN profiles
UNRES-flex
Xray data
spearman= 0.53
pearson= 0.52
UNRES-DSSP-flex
Xray data
spearman= 0.5
pearson= 0.59
CABS-flex
Xray data
spearman= 0.43
pearson= 0.54
NOLB
Xray data
spearman= 0.49
pearson= 0.55
RMSFN
Residue index

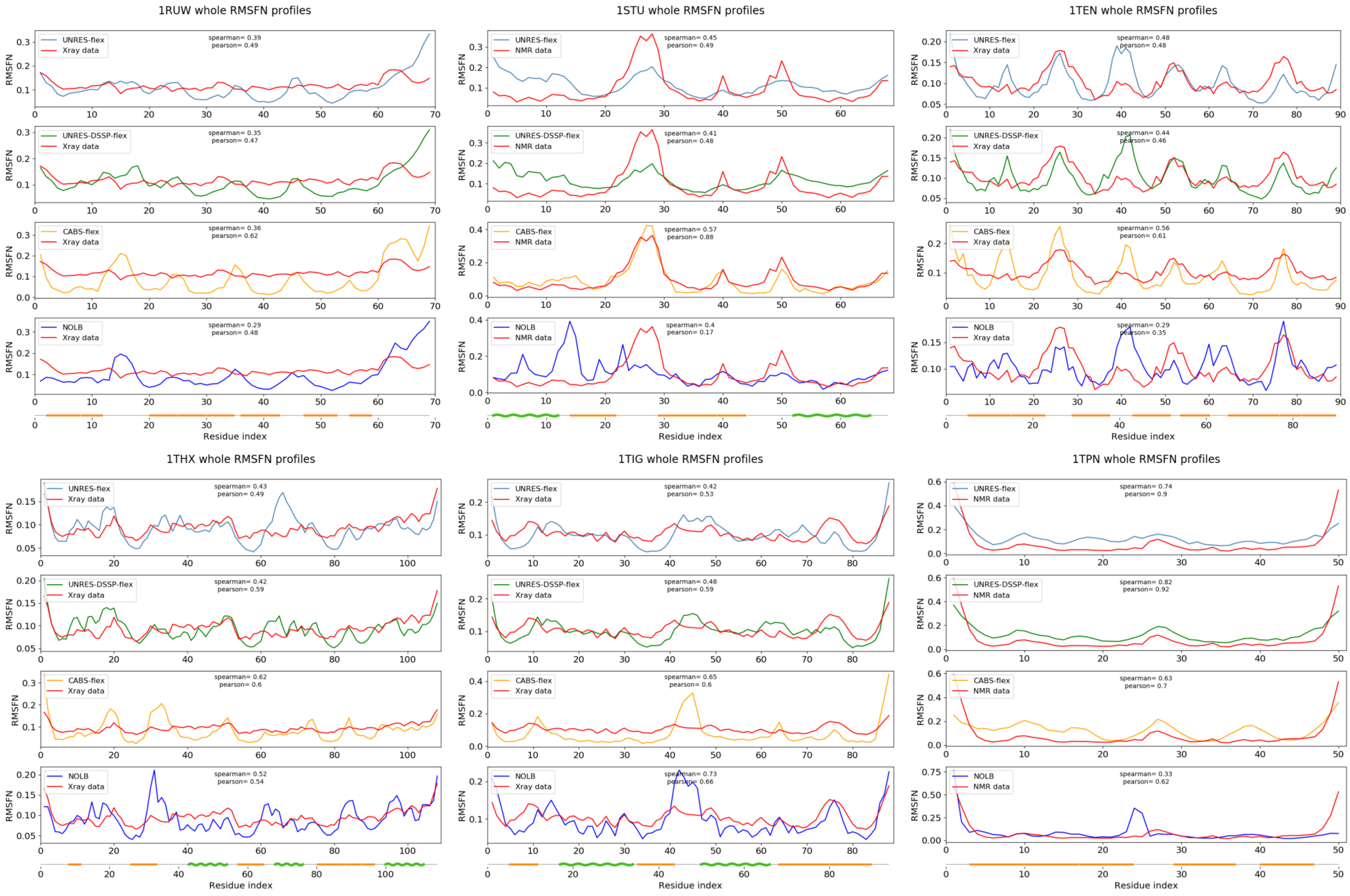
1RUW whole RMSFN profiles
UNRES-flex
Xray data
spearman= 0.39
pearson= 0.49
UNRES-DSSP-flex
Xray data
spearman= 0.35
pearson= 0.47
CABS-flex
Xray data
spearman= 0.36
pearson= 0.62
NOLB
Xray data
spearman= 0.29
pearson= 0.48
RMSFN
Residue index
1STU whole RMSFN profiles
UNRES-flex
NMR data
spearman= 0.45
pearson= 0.49
UNRES-DSSP-flex
NMR data
spearman= 0.41
pearson= 0.48
CABS-flex
NMR data
spearman= 0.57
pearson= 0.88
NOLB
NMR data
spearman= 0.4
pearson= 0.17
1TEN whole RMSFN profiles
UNRES-flex
Xray data
spearman= 0.48
pearson= 0.48
UNRES-DSSP-flex
Xray data
spearman= 0.44
pearson= 0.46
CABS-flex
Xray data
spearman= 0.56
pearson= 0.61
NOLB
Xray data
spearman= 0.29
pearson= 0.35
1THX whole RMSFN profiles
UNRES-flex
Xray data
spearman= 0.43
pearson= 0.49
UNRES-DSSP-flex
Xray data
spearman= 0.42
pearson= 0.59
CABS-flex
Xray data
spearman= 0.62
pearson= 0.6
NOLB
Xray data
spearman= 0.52
pearson= 0.54
1TIG whole RMSFN profiles
UNRES-flex
Xray data
spearman= 0.42
pearson= 0.53
UNRES-DSSP-flex
Xray data
spearman= 0.48
pearson= 0.59
CABS-flex
Xray data
spearman= 0.65
pearson= 0.6
NOLB
Xray data
spearman= 0.73
pearson= 0.66
1TPN whole RMSFN profiles
UNRES-flex
NMR data
spearman= 0.74
pearson= 0.9
UNRES-DSSP-flex
NMR data
spearman= 0.82
pearson= 0.92
CABS-flex
NMR data
spearman= 0.63
pearson= 0.7
NOLB
NMR data
spearman= 0.33
pearson= 0.62

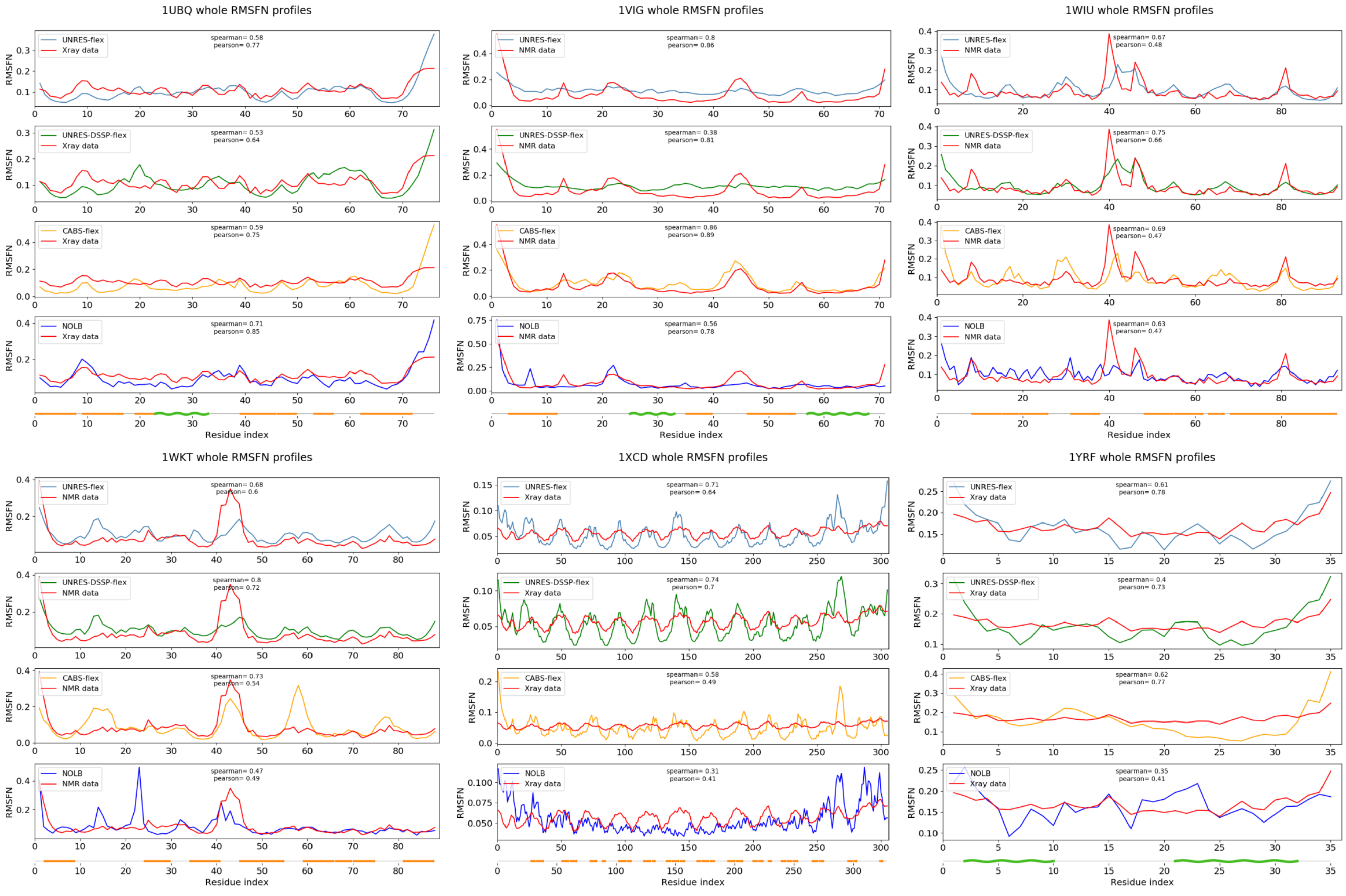
1UBQ whole RMSFN profiles
UNRES-flex
Xray data
spearman= 0.58
pearson= 0.77
UNRES-DSSP-flex
Xray data
spearman= 0.53
pearson= 0.64
CABS-flex
Xray data
spearman= 0.59
pearson= 0.75
NOLB
Xray data
spearman= 0.71
pearson= 0.85
1VIG whole RMSFN profiles
UNRES-flex
NMR data
spearman= 0.8
pearson= 0.86
UNRES-DSSP-flex
NMR data
spearman= 0.38
pearson= 0.81
CABS-flex
NMR data
spearman= 0.86
pearson= 0.89
NOLB
NMR data
spearman= 0.56
pearson= 0.78
1WIU whole RMSFN profiles
UNRES-flex
NMR data
spearman= 0.67
pearson= 0.48
UNRES-DSSP-flex
NMR data
spearman= 0.75
pearson= 0.66
CABS-flex
NMR data
spearman= 0.69
pearson= 0.47
NOLB
NMR data
spearman= 0.63
pearson= 0.47
1WKT whole RMSFN profiles
UNRES-flex
NMR data
spearman= 0.68
pearson= 0.6
UNRES-DSSP-flex
NMR data
spearman= 0.8
pearson= 0.72
CABS-flex
NMR data
spearman= 0.73
pearson= 0.54
NOLB
NMR data
spearman= 0.47
pearson= 0.49
1XCD whole RMSFN profiles
UNRES-flex
Xray data
spearman= 0.71
pearson= 0.64
UNRES-DSSP-flex
Xray data
spearman= 0.74
pearson= 0.7
CABS-flex
Xray data
spearman= 0.58
pearson= 0.49
NOLB
Xray data
spearman= 0.31
pearson= 0.41
1YRF whole RMSFN profiles
UNRES-flex
Xray data
spearman= 0.61
pearson= 0.78
UNRES-DSSP-flex
Xray data
spearman= 0.4
pearson= 0.73
CABS-flex
Xray data
spearman= 0.62
pearson= 0.77
NOLB
Xray data
spearman= 0.35
pearson= 0.41
RMSFN
Residue index

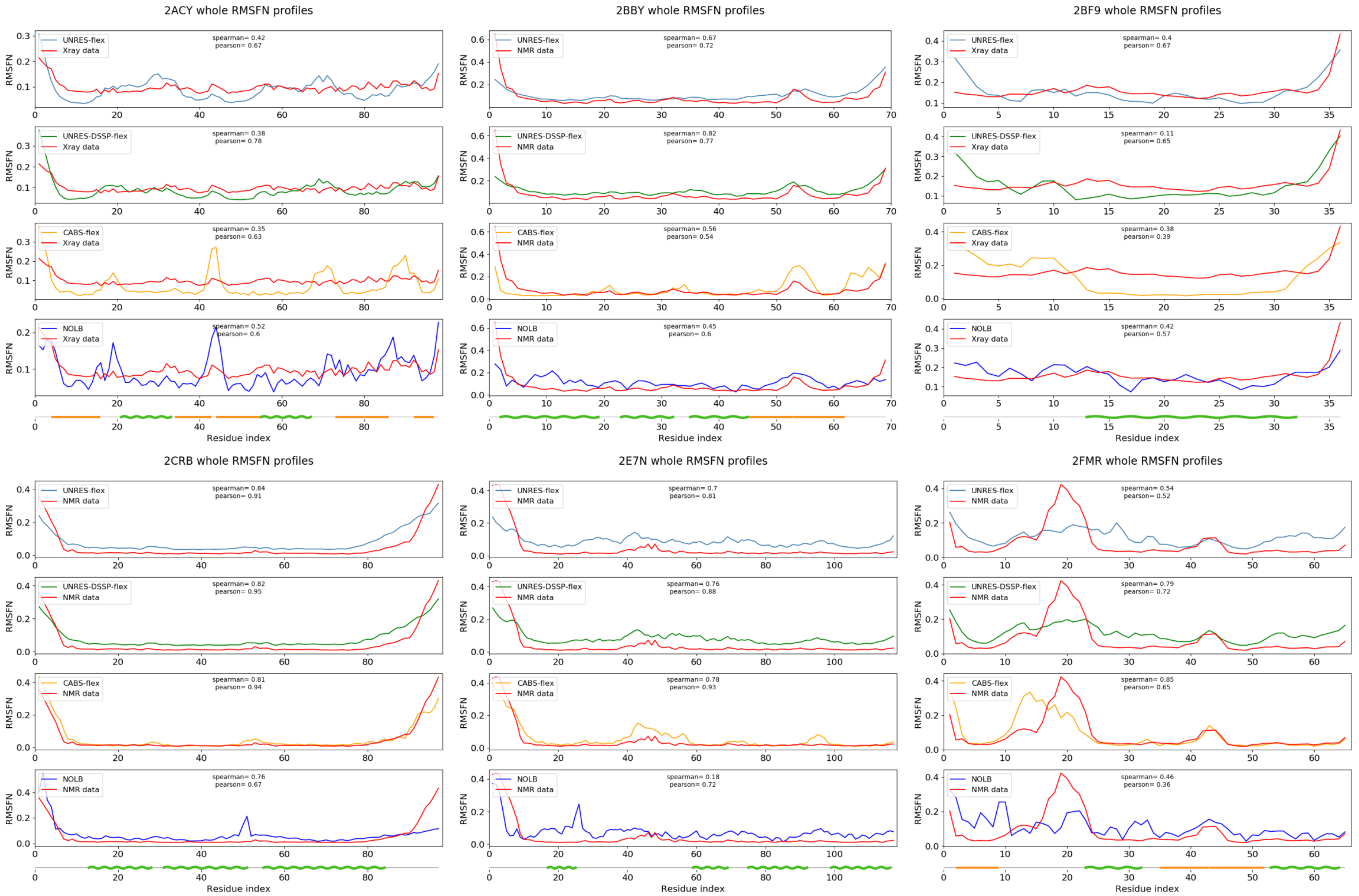
2ACY whole RMSFN profiles
UNRES-flex
Xray data
spearman= 0.42
pearson= 0.67
UNRES-DSSP-flex
Xray data
spearman= 0.38
pearson= 0.78
CABS-flex
Xray data
spearman= 0.35
pearson= 0.63
NOLB
Xray data
spearman= 0.52
pearson= 0.6
2BBY whole RMSFN profiles
UNRES-flex
NMR data
spearman= 0.67
pearson= 0.72
UNRES-DSSP-flex
NMR data
spearman= 0.82
pearson= 0.77
CABS-flex
NMR data
spearman= 0.56
pearson= 0.54
NOLB
NMR data
spearman= 0.45
pearson= 0.6
2BF9 whole RMSFN profiles
UNRES-flex
Xray data
spearman= 0.4
pearson= 0.67
UNRES-DSSP-flex
Xray data
spearman= 0.11
pearson= 0.65
CABS-flex
Xray data
spearman= 0.38
pearson= 0.39
NOLB
Xray data
spearman= 0.42
pearson= 0.57
2CRB whole RMSFN profiles
UNRES-flex
NMR data
spearman= 0.84
pearson= 0.91
UNRES-DSSP-flex
NMR data
spearman= 0.82
pearson= 0.95
CABS-flex
NMR data
spearman= 0.81
pearson= 0.94
NOLB
NMR data
spearman= 0.76
pearson= 0.67
2E7N whole RMSFN profiles
UNRES-flex
NMR data
spearman= 0.7
pearson= 0.81
UNRES-DSSP-flex
NMR data
spearman= 0.76
pearson= 0.88
CABS-flex
NMR data
spearman= 0.78
pearson= 0.93
NOLB
NMR data
spearman= 0.18
pearson= 0.72
2FMR whole RMSFN profiles
UNRES-flex
NMR data
spearman= 0.54
pearson= 0.52
UNRES-DSSP-flex
NMR data
spearman= 0.79
pearson= 0.72
CABS-flex
NMR data
spearman= 0.85
pearson= 0.65
NOLB
NMR data
spearman= 0.46
pearson= 0.36
RMSFN
Residue index

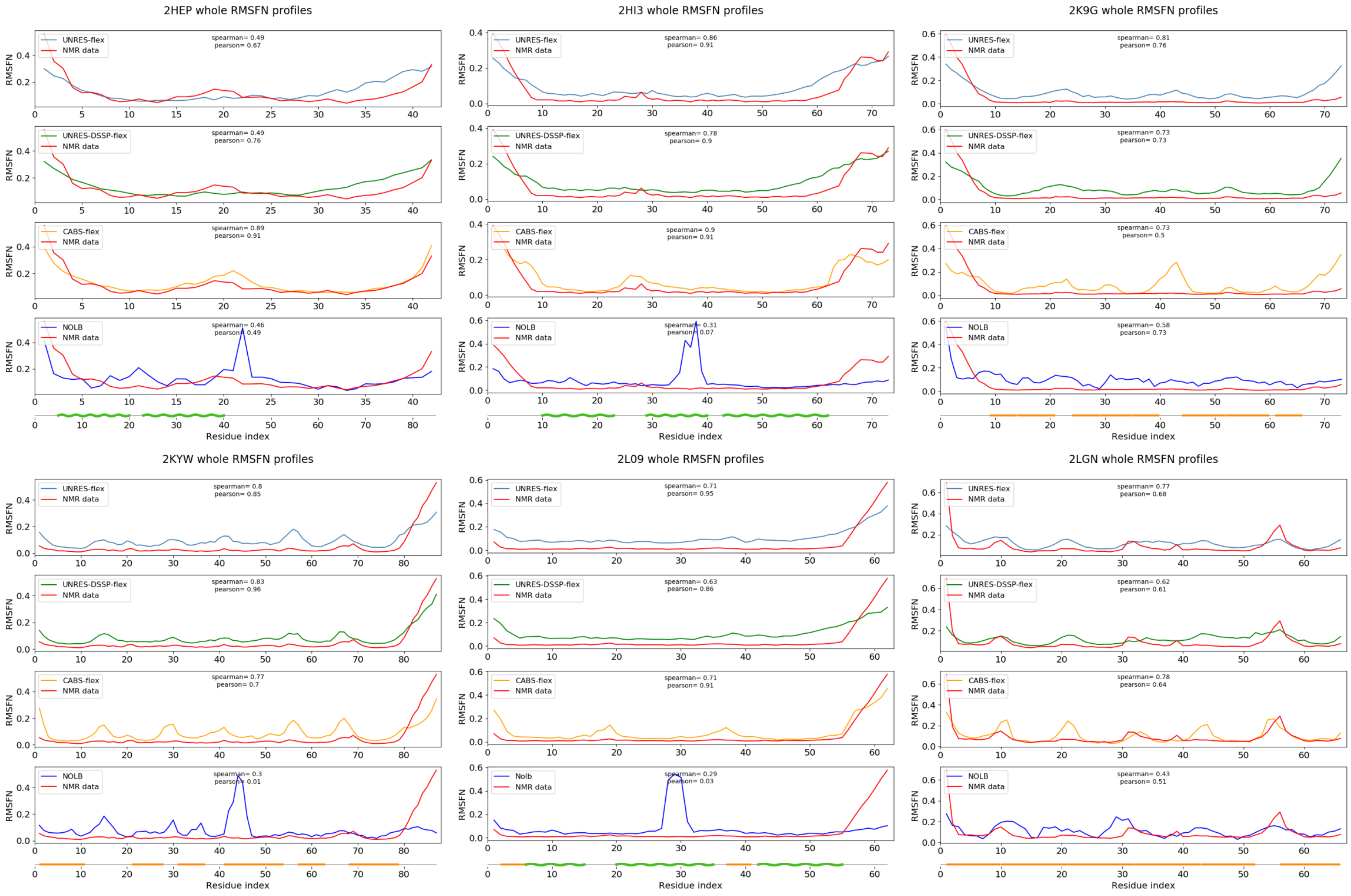

2HEP whole RMSFN profiles
UNRES-flex
NMR data
spearman= 0.49
pearson= 0.67
UNRES-DSSP-flex
spearman= 0.49
pearson= 0.76
CABS-flex
spearman= 0.89
pearson= 0.91
NOLB
spearman= 0.46
pearson= 0.49
2HI3 whole RMSFN profiles
spearman= 0.86
pearson= 0.91
spearman= 0.78
pearson= 0.9
spearman= 0.9
pearson= 0.91
spearman= 0.31
pearson= 0.07
2K9G whole RMSFN profiles
spearman= 0.81
pearson= 0.76
spearman= 0.73
pearson= 0.73
spearman= 0.73
pearson= 0.5
spearman= 0.58
pearson= 0.73
2KYW whole RMSFN profiles
spearman= 0.8
pearson= 0.85
spearman= 0.83
pearson= 0.96
spearman= 0.77
pearson= 0.7
spearman= 0.3
pearson= 0.01
2L09 whole RMSFN profiles
Nolb
spearman= 0.71
pearson= 0.95
spearman= 0.63
pearson= 0.86
spearman= 0.71
pearson= 0.91
spearman= 0.29
pearson= 0.03
2LGN whole RMSFN profiles
spearman= 0.77
pearson= 0.68
spearman= 0.62
pearson= 0.61
spearman= 0.78
pearson= 0.64
spearman= 0.43
pearson= 0.51
RMSFN
Residue index

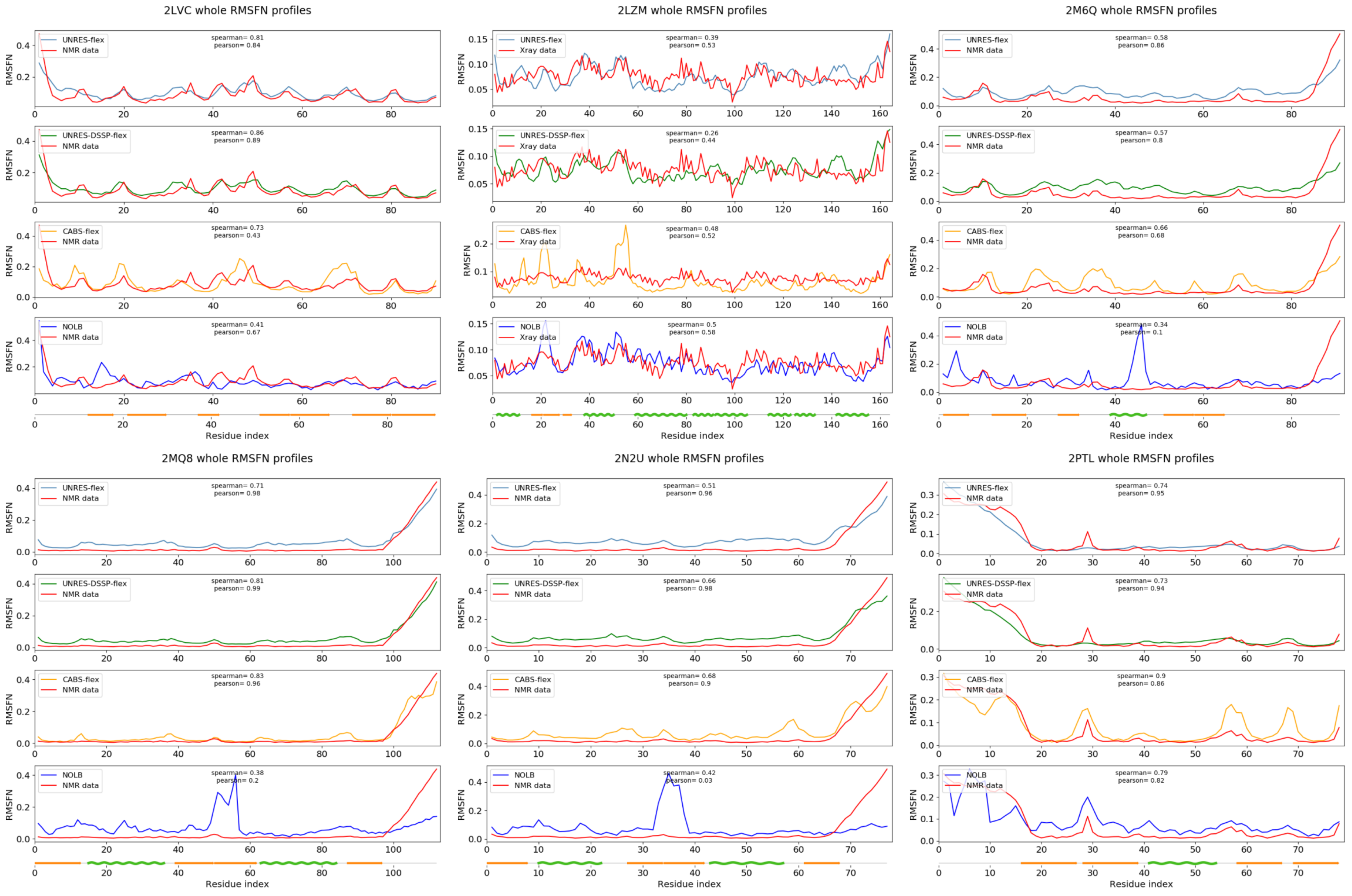
2LVC whole RMSFN profiles
UNRES-flex
NMR data
spearman= 0.81
pearson= 0.84
UNRES-DSSP-flex
NMR data
spearman= 0.86
pearson= 0.89
CABS-flex
NMR data
spearman= 0.73
pearson= 0.43
NOLB
NMR data
spearman= 0.41
pearson= 0.67
2LZM whole RMSFN profiles
UNRES-flex
Xray data
spearman= 0.39
pearson= 0.53
UNRES-DSSP-flex
Xray data
spearman= 0.26
pearson= 0.44
CABS-flex
Xray data
spearman= 0.48
pearson= 0.52
NOLB
Xray data
spearman= 0.5
pearson= 0.58
2M6Q whole RMSFN profiles
UNRES-flex
NMR data
spearman= 0.58
pearson= 0.86
UNRES-DSSP-flex
NMR data
spearman= 0.57
pearson= 0.8
CABS-flex
NMR data
spearman= 0.66
pearson= 0.68
NOLB
NMR data
spearman= 0.34
pearson= 0.1
2MQ8 whole RMSFN profiles
UNRES-flex
NMR data
spearman= 0.71
pearson= 0.98
UNRES-DSSP-flex
NMR data
spearman= 0.81
pearson= 0.99
CABS-flex
NMR data
spearman= 0.83
pearson= 0.96
NOLB
NMR data
spearman= 0.38
pearson= 0.2
2N2U whole RMSFN profiles
UNRES-flex
NMR data
spearman= 0.51
pearson= 0.96
UNRES-DSSP-flex
NMR data
spearman= 0.66
pearson= 0.98
CABS-flex
NMR data
spearman= 0.68
pearson= 0.9
NOLB
NMR data
spearman= 0.42
pearson= 0.03
2PTL whole RMSFN profiles
UNRES-flex
NMR data
spearman= 0.74
pearson= 0.95
UNRES-DSSP-flex
NMR data
spearman= 0.73
pearson= 0.94
CABS-flex
NMR data
spearman= 0.9
pearson= 0.86
NOLB
NMR data
spearman= 0.79
pearson= 0.82
RMSFN
Residue index

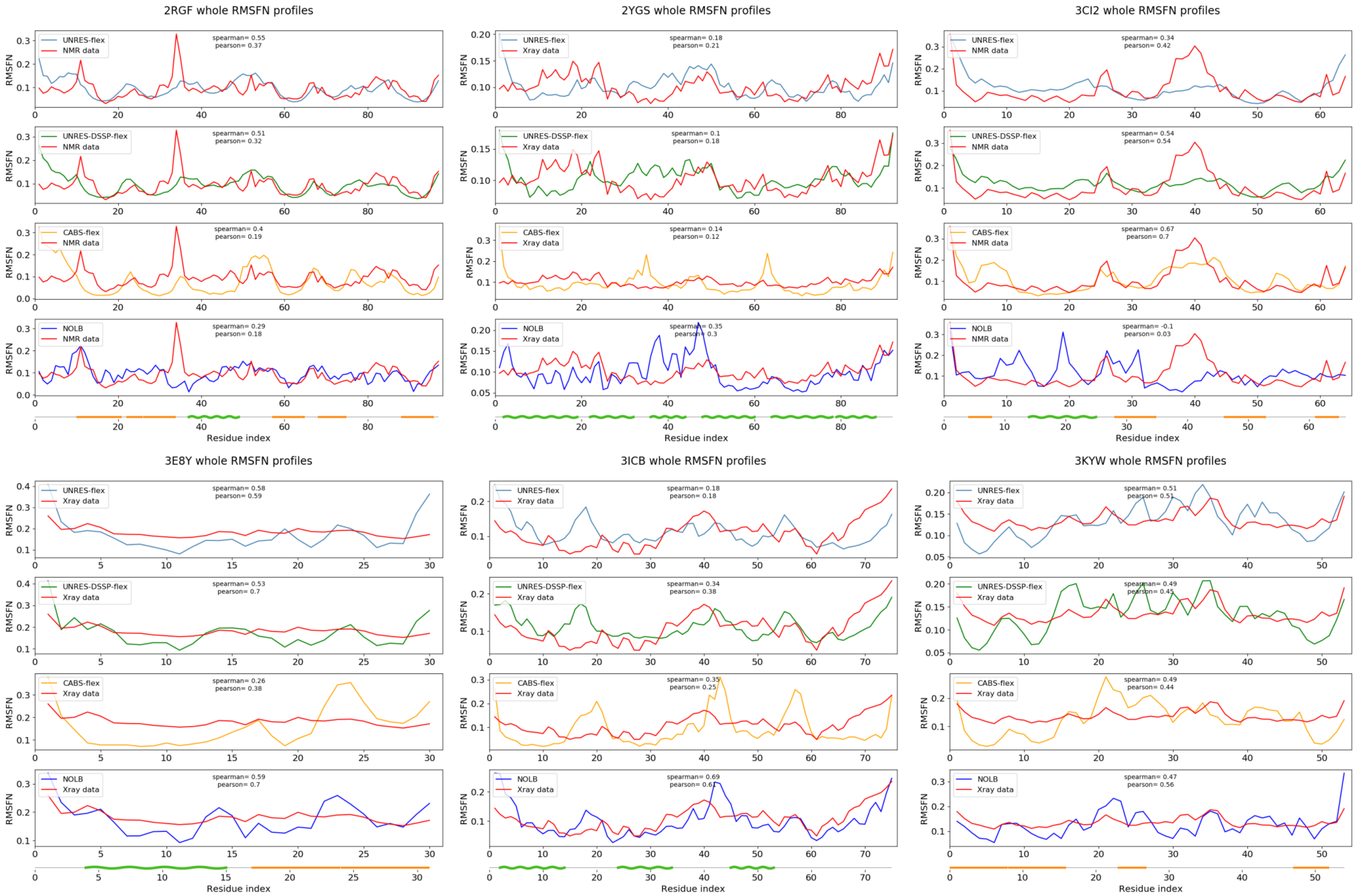
2RGF whole RMSFN profiles
UNRES-flex
NMR data
spearman= 0.55
pearson= 0.37
UNRES-DSSP-flex
NMR data
spearman= 0.51
pearson= 0.32
CABS-flex
NMR data
spearman= 0.4
pearson= 0.19
NOLB
NMR data
spearman= 0.29
pearson= 0.18
RMSFN
Residue index
2YGS whole RMSFN profiles
UNRES-flex
Xray data
spearman= 0.18
pearson= 0.21
UNRES-DSSP-flex
Xray data
spearman= 0.1
pearson= 0.18
CABS-flex
Xray data
spearman= 0.14
pearson= 0.12
NOLB
Xray data
spearman= 0.35
pearson= 0.3
3CI2 whole RMSFN profiles
UNRES-flex
NMR data
spearman= 0.34
pearson= 0.42
UNRES-DSSP-flex
NMR data
spearman= 0.54
pearson= 0.54
CABS-flex
NMR data
spearman= 0.67
pearson= 0.7
NOLB
NMR data
spearman= -0.1
pearson= 0.03
3E8Y whole RMSFN profiles
UNRES-flex
Xray data
spearman= 0.58
pearson= 0.59
UNRES-DSSP-flex
Xray data
spearman= 0.53
pearson= 0.7
CABS-flex
Xray data
spearman= 0.26
pearson= 0.38
NOLB
Xray data
spearman= 0.59
pearson= 0.7
3ICB whole RMSFN profiles
UNRES-flex
Xray data
spearman= 0.18
pearson= 0.18
UNRES-DSSP-flex
Xray data
spearman= 0.34
pearson= 0.38
CABS-flex
Xray data
spearman= 0.35
pearson= 0.25
NOLB
Xray data
spearman= 0.69
pearson= 0.61
3KYW whole RMSFN profiles
UNRES-flex
Xray data
spearman= 0.51
pearson= 0.51
UNRES-DSSP-flex
Xray data
spearman= 0.49
pearson= 0.45
CABS-flex
Xray data
spearman= 0.49
pearson= 0.44
NOLB
Xray data
spearman= 0.47
pearson= 0.56

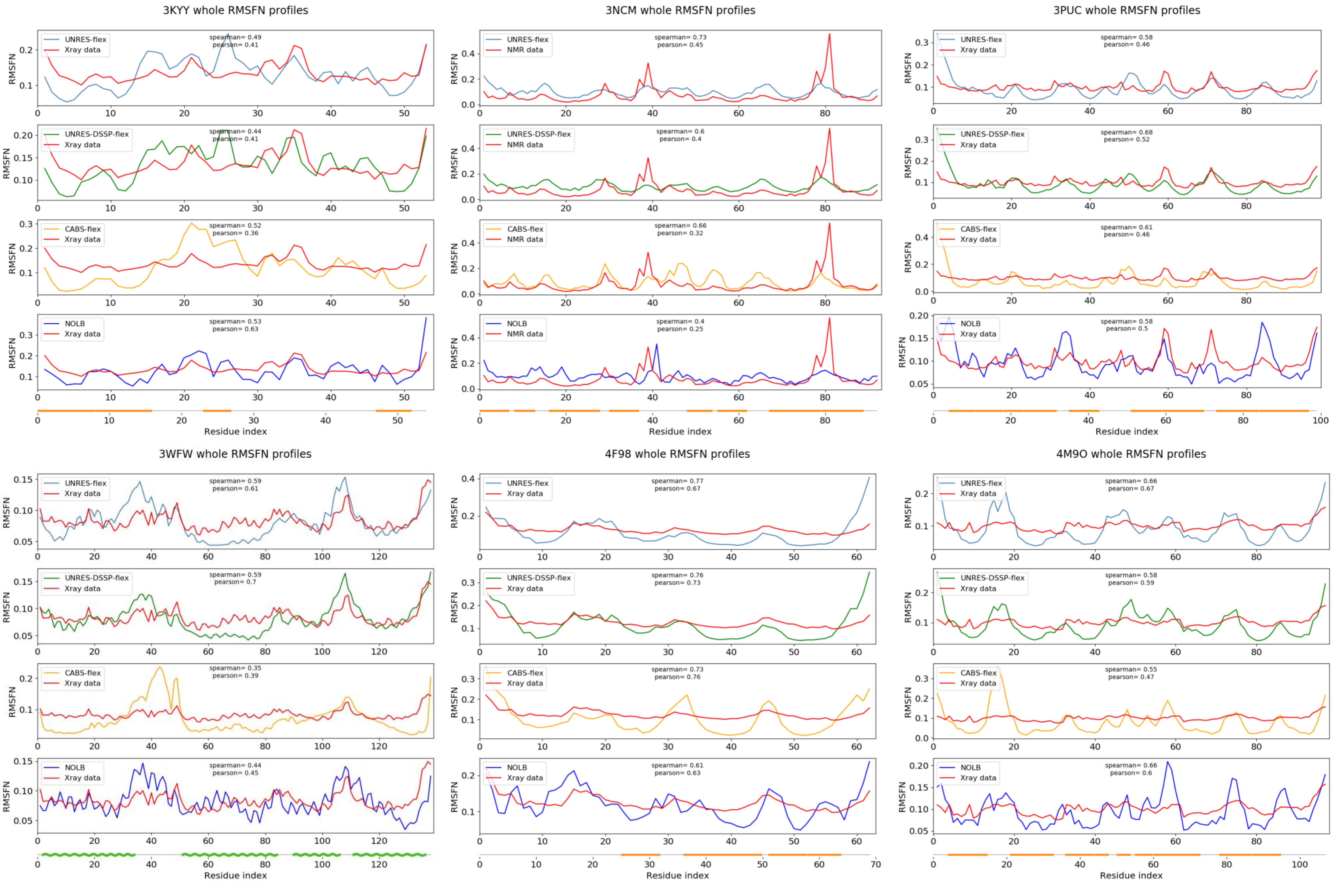
3KYY whole RMSFN profiles
UNRES-flex
Xray data
spearman= 0.49
pearson= 0.41
UNRES-DSSP-flex
Xray data
spearman= 0.44
pearson= 0.41
CABS-flex
Xray data
spearman= 0.52
pearson= 0.36
NOLB
Xray data
spearman= 0.53
pearson= 0.63
RMSFN
Residue index
3NCM whole RMSFN profiles
UNRES-flex
NMR data
spearman= 0.73
pearson= 0.45
UNRES-DSSP-flex
NMR data
spearman= 0.6
pearson= 0.4
CABS-flex
NMR data
spearman= 0.66
pearson= 0.32
NOLB
NMR data
spearman= 0.4
pearson= 0.25
RMSFN
Residue index
3PUC whole RMSFN profiles
UNRES-flex
Xray data
spearman= 0.58
pearson= 0.46
UNRES-DSSP-flex
Xray data
spearman= 0.68
pearson= 0.52
CABS-flex
Xray data
spearman= 0.61
pearson= 0.46
NOLB
Xray data
spearman= 0.58
pearson= 0.5
RMSFN
Residue index
3WFW whole RMSFN profiles
UNRES-flex
Xray data
spearman= 0.59
pearson= 0.61
UNRES-DSSP-flex
Xray data
spearman= 0.59
pearson= 0.7
CABS-flex
Xray data
spearman= 0.35
pearson= 0.39
NOLB
Xray data
spearman= 0.44
pearson= 0.45
RMSFN
Residue index
4F98 whole RMSFN profiles
UNRES-flex
Xray data
spearman= 0.77
pearson= 0.67
UNRES-DSSP-flex
Xray data
spearman= 0.76
pearson= 0.73
CABS-flex
Xray data
spearman= 0.73
pearson= 0.76
NOLB
Xray data
spearman= 0.61
pearson= 0.63
RMSFN
Residue index
4M9O whole RMSFN profiles
UNRES-flex
Xray data
spearman= 0.66
pearson= 0.67
UNRES-DSSP-flex
Xray data
spearman= 0.58
pearson= 0.59
CABS-flex
Xray data
spearman= 0.55
pearson= 0.47
NOLB
Xray data
spearman= 0.66
pearson= 0.6
RMSFN
Residue index

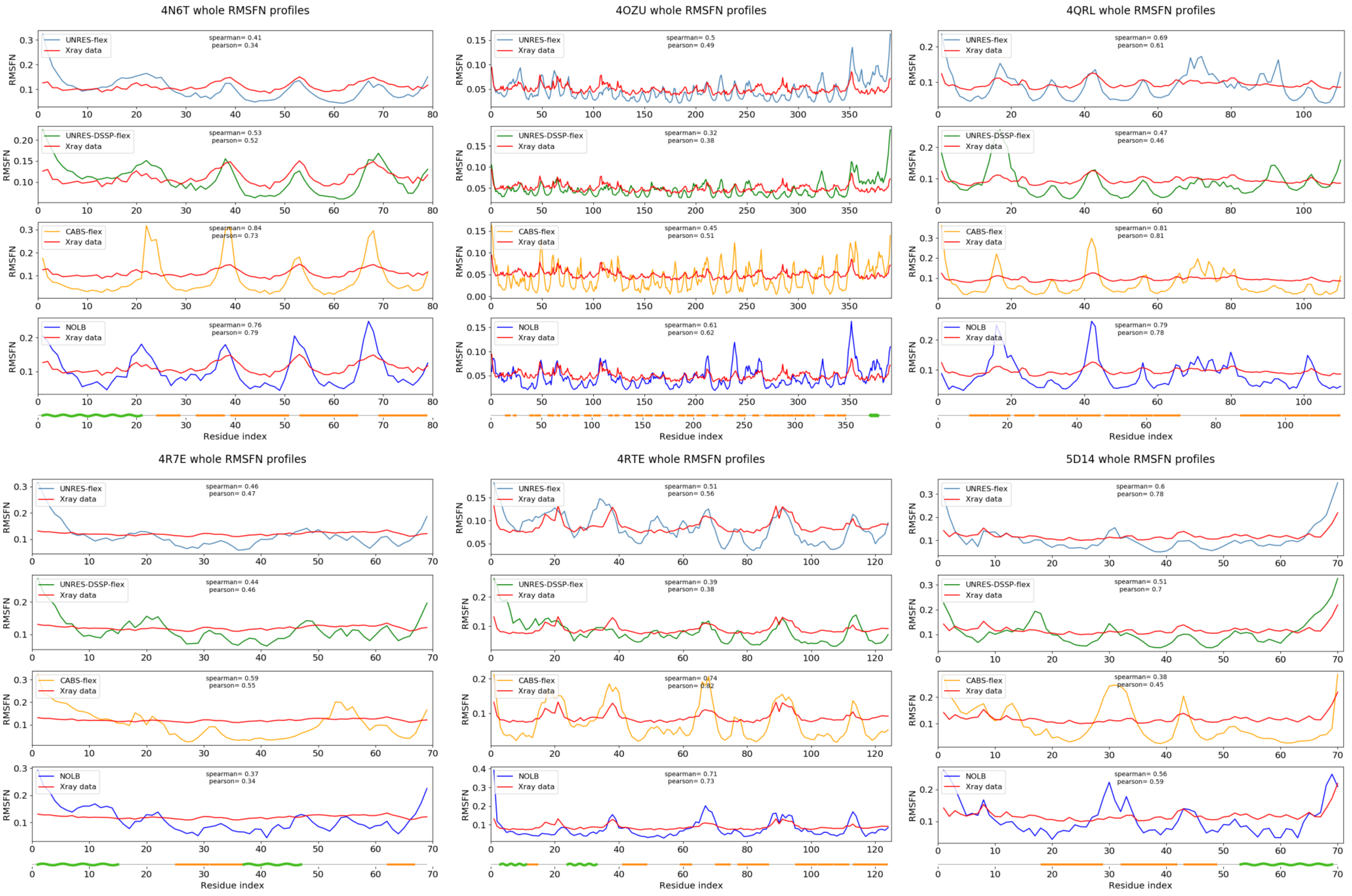
4N6T whole RMSFN profiles
UNRES-flex
Xray data
spearman= 0.41
pearson= 0.34
UNRES-DSSP-flex
Xray data
spearman= 0.53
pearson= 0.52
CABS-flex
Xray data
spearman= 0.84
pearson= 0.73
NOLB
Xray data
spearman= 0.76
pearson= 0.79
RMSFN
Residue index
4OZU whole RMSFN profiles
UNRES-flex
Xray data
spearman= 0.5
pearson= 0.49
UNRES-DSSP-flex
Xray data
spearman= 0.32
pearson= 0.38
CABS-flex
Xray data
spearman= 0.45
pearson= 0.51
NOLB
Xray data
spearman= 0.61
pearson= 0.62
RMSFN
Residue index
4QRL whole RMSFN profiles
UNRES-flex
Xray data
spearman= 0.69
pearson= 0.61
UNRES-DSSP-flex
Xray data
spearman= 0.47
pearson= 0.46
CABS-flex
Xray data
spearman= 0.81
pearson= 0.81
NOLB
Xray data
spearman= 0.79
pearson= 0.78
RMSFN
Residue index
4R7E whole RMSFN profiles
UNRES-flex
Xray data
spearman= 0.46
pearson= 0.47
UNRES-DSSP-flex
Xray data
spearman= 0.44
pearson= 0.46
CABS-flex
Xray data
spearman= 0.59
pearson= 0.55
NOLB
Xray data
spearman= 0.37
pearson= 0.34
RMSFN
Residue index
4RTE whole RMSFN profiles
UNRES-flex
Xray data
spearman= 0.51
pearson= 0.56
UNRES-DSSP-flex
Xray data
spearman= 0.39
pearson= 0.38
CABS-flex
Xray data
spearman= 0.74
pearson= 0.82
NOLB
Xray data
spearman= 0.71
pearson= 0.73
RMSFN
Residue index
5D14 whole RMSFN profiles
UNRES-flex
Xray data
spearman= 0.6
pearson= 0.78
UNRES-DSSP-flex
Xray data
spearman= 0.51
pearson= 0.7
CABS-flex
Xray data
spearman= 0.38
pearson= 0.45
NOLB
Xray data
spearman= 0.56
pearson= 0.59
RMSFN
Residue index

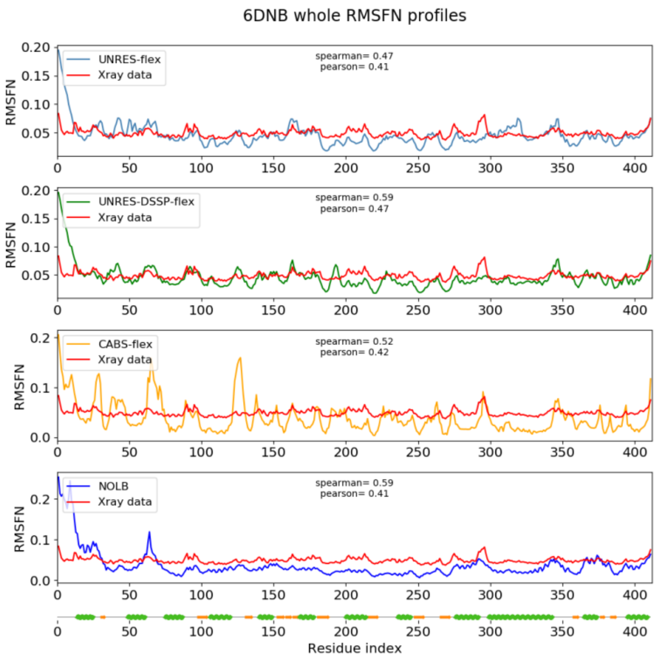


**Figure S3.** Scatter plots of the Pearson ($r_p$) and Spearman ($r_s$) coefficients between the experimental and predicted fluctuation profiles in chain length for the NMR (A-D) and X-ray (E-H) structures and the UNRES-flex (A, E), UNRES-DSSP-flex (B, F), CABS-flex (C,G), and NOLB (D, H) prediction methods.

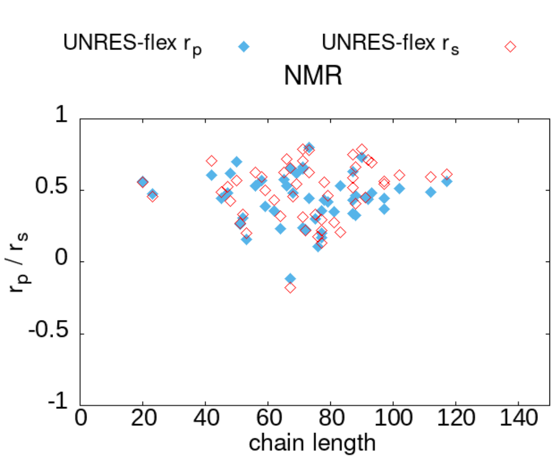


(A)

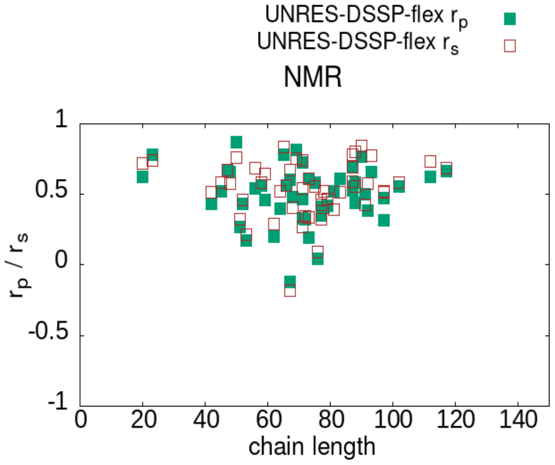


(B)

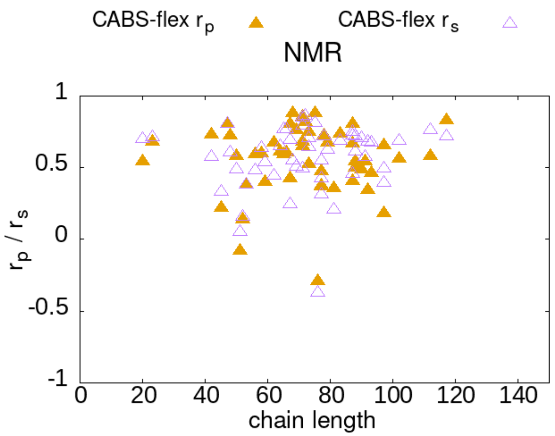
CABS-flex $r_p$
CABS-flex $r_s$
NMR
1
0.5
0
-0.5
-1
$r_p$ / $r_s$
0 20 40 60 80 100 120 140
chain length

(C)

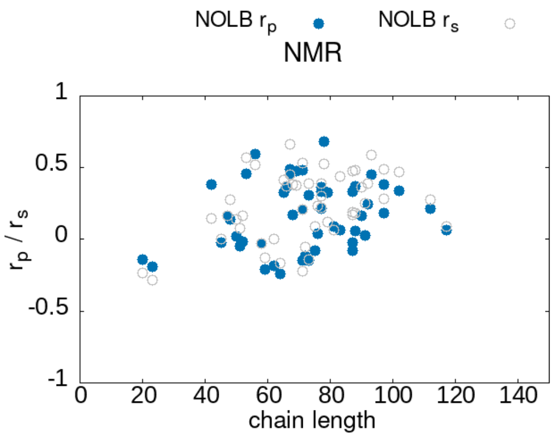
NOLB $r_p$
NOLB $r_s$
NMR
1
0.5
0
-0.5
-1
$r_p$ / $r_s$
0 20 40 60 80 100 120 140
chain length

(D)

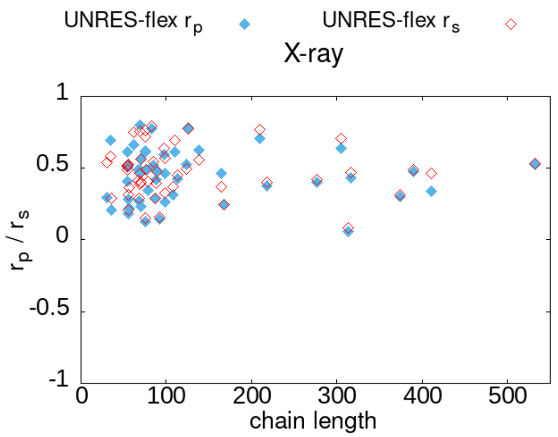
UNRES-flex $r_p$
UNRES-flex $r_s$
X-ray
1
0.5
0
-0.5
-1
$r_p$ / $r_s$
0 100 200 300 400 500
chain length

(E)

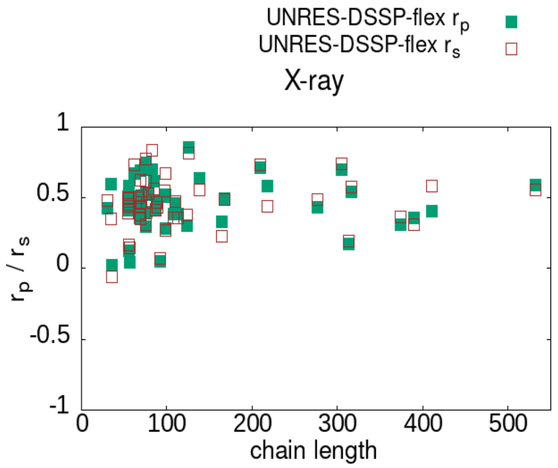
UNRES-DSSP-flex $r_p$
UNRES-DSSP-flex $r_s$
X-ray
1
0.5
0
-0.5
-1
$r_p$ / $r_s$
0 100 200 300 400 500
chain length

(F)

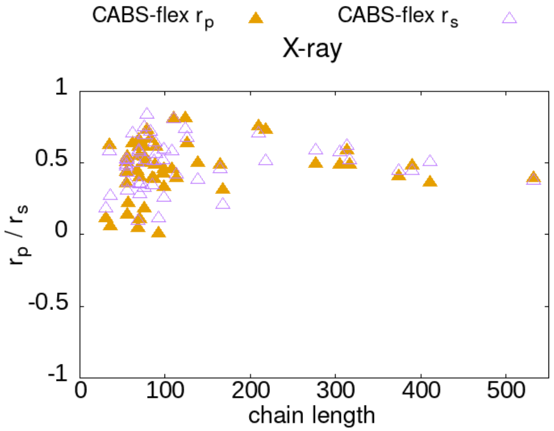
CABS-flex $r_p$
CABS-flex $r_s$
X-ray
$r_p$ / $r_s$
1
0.5
0
-0.5
-1
0
100
200
300
400
500
chain length
(G)

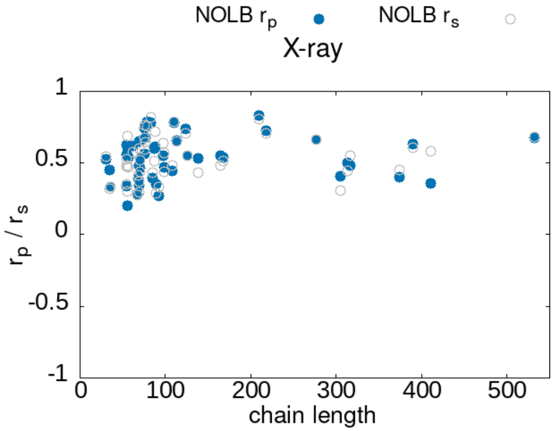
NOLB $r_p$
NOLB $r_s$
X-ray
$r_p$ / $r_s$
1
0.5
0
-0.5
-1
0
100
200
300
400
500
chain length
(H)
